# The use of the Godin-Shephard Leisure-Time Physical Activity Questionnaire in oncology research: a systematic review

**DOI:** 10.1186/s12874-015-0045-7

**Published:** 2015-08-12

**Authors:** Steve Amireault, Gaston Godin, Jason Lacombe, Catherine M. Sabiston

**Affiliations:** Department of Health and Kinesiology, Purdue University, Lambert Fieldhouse, 800 W. Stadium Ave. Room 311A, West Lafayette, IN 47907-2046, USA; Department of Psychology, Faculty of Arts and Science, Concordia University, Montreal, QC Canada; Faculty of Nursing, Université Laval, 1050 Avenue de la Médecine, Pavillon Ferdinand-Vandry, Quebec City, G1V 0A4 QC Canada

**Keywords:** Cancer, Exercise, Neoplasm, Oncology, Questionnaires, Reproducibility of results

## Abstract

**Background:**

The Godin-Shephard Leisure-Time Physical Activity Questionnaire (GSLTPAQ) is one of the most often used questionnaires in oncology research, yet modifications to the scale are done with little evidence of psychometric testing. This study aimed to (i) document the frequency of use of the questionnaire for ranking (i.e., level of activity) and classification (i.e., *active* versus *insufficiently active*) purposes, (ii) summarize how the GSLTPAQ is used in terms of item content and scoring, and (iii) evaluate the extent to which validity evidence supports the use of the scale among cancer survivors.

**Methods:**

A systematic review was conducted with evidence drawn from English-written articles published between January 1^st^ 1985 (year the GSLTPAQ was published) and December 31, 2014. A search of six databases, a scan of reference list of included articles, and a cited reference search identified articles that reported using the GSLTPAQ among cancer survivors.

**Results:**

A total of 212 articles were retrieved. The GSLTPAQ was used for classifying cancer survivors into *active* and *insufficiently active* categories in 51 % of the articles. Moreover, a modified version of the questionnaire was used in 81 % of the research studies. Three studies reported validity evidence based on the relationship between the scores on the GSLTPAQ (i.e., leisure score index, LSI) and accelerometer or pedometer-derived activity data. Validity evidence supporting the use of the GSLTPAQ for assessing changes in LSI was computed from six randomized trials.

**Conclusions:**

The use of the GSLTPAQ for classification purpose in oncology research is common. Standardization in the use and interpretation of the GSLTPAQ in oncology research is warranted. Although limited, there is support for using the original form of the GSLTPAQ and interpreting the LSI for ranking cancer survivors from the lowest to highest levels of leisure-time physical activity.

**Electronic supplementary material:**

The online version of this article (doi:10.1186/s12874-015-0045-7) contains supplementary material, which is available to authorized users.

## Background

Leisure-time physical activity (LTPA) is an important subtype of physical activity (PA) for research and behavior change intervention in oncology context. PA is defined as ‘any bodily movement produced by skeletal muscles that results in energy expenditure’ [1] (p.126), whereas LTPA refers to any ‘[…] activity undertaken in the individual’s discretionary time that increases the total energy expenditure’ [2] (p. 12). Compared to household, occupational, and commuting PA, LTPA is likely to be more volitional and performed at higher intensity [1, 3, 4], which may provide greater fitness- and health- related benefits [5, 6]. In addition, LTPA, which includes exercise training [1, 2], is safe and rewarding for both physical and mental health among cancer survivors [6–10].

The Godin-Shephard Leisure-Time Physical Activity Questionnaire (GSLTPAQ; [11–13]) is a short questionnaire that is often used to assess LTPA in oncology research [14, 15]. The GSLTPAQ is a 4-item self-administered questionnaire with the first three questions seeking information on the number of times one engages in mild, moderate and strenuous LTPA bouts of at least 15 min duration in a typical week. Examples of LTPA are provided for each intensity category (for a complete version of the questionnaire, readers are referred to Godin [12]). Scores derived from the GSLTPAQ include total weekly LTPA, called a Leisure Score Index (LSI), in which number of bouts at each intensity is multiplied by 3, 5, and 9 metabolic equivalents (METs) and summed. LSI scores can be used for ranking individuals from the lowest to highest PA levels [16]. In addition, the score obtained from moderate and strenuous LTPA can be used to classify respondents into *active* and *insufficiently active* categories according to published PA guidelines for public health [17–19] and cancer survivors [7, 9].

The GSLTPAQ is one of the potential measures of PA that the Division of Cancer Epidemiology & Genetics research program of the National Cancer Institute recommends to oncology clinicians and researchers [20]. However, the questionnaire was not reviewed or analysed by their technical Evaluation Committee, and suggested that it should be viewed “as starting points that can be adapted or improved upon as appropriate” [21]. Additionally, a limited amount and variety of validity evidence supporting the use of the GSLTPAQ among cancer survivors has been accumulated. This is an important drawback as validity evidence supporting the use of this scale among apparently healthy adults (e.g., [5, 11, 22–25]) may not generalize to cancer survivors as measurement properties may differ across populations [26, 27]. For instance, cancer survivors’ cognitive abilities (e.g., information processing, attention, concentration, memory) needed for effective recall and reporting of PA, may have been impaired by the disease itself or its treatments [9, 28]. As a result, risk of recall bias may be higher in cancer survivors than in apparently healthy individuals, especially for older and metastatic cancer survivors. Accurate reporting of PA intensity may also be more challenging for someone going through cancer treatment because the perception of PA intensity may not reflect the intensity of a given PA described in the questionnaire [26, 29]. Furthermore, many researchers have adapted the questionnaire for their own purposes without acknowledging the implications of these adaptations on its measurement quality. This is a meaningful shortcoming because the measurement quality of these adapted versions of the GSLTPAQ is unknown and using poor quality LTPA questionnaires increases the risk of misclassification and biased results. Clinically relevant associations between LTPA, assessed either as an exposure or an outcome, and any other relevant variables can be mitigated or may remain undetected when interpreting scores from poor quality questionnaires [29–31].

In order to provide safe and effective recommendations to cancer survivors, it is critical to use questionnaires that offer an optimal trade-off between quality and feasibility to best capture and understand PA among cancer survivors. Given that the quality of a measurement tool depends on its intended use and interpretation among a given population [32], the objectives of this review are (i) to document the frequency of use of the GSLTPAQ for ranking and classification purposes, (ii) to summarize how the GSLTPAQ is used in terms of item content and scoring methods, and (iii) to evaluate the extent to which validity evidence supports the use of the GSLTPAQ among cancer survivors. As the GSLTPAQ is inexpensive, does not require specific skills for completion or interpretation, and can be administered to a large number of cancer survivors quickly and efficiently, identifying validity evidence can help facilitate opportunities for data collection, patient monitoring and survivorship care planning, as well as outcomes and practice-based research. This may be particularly important for research on PA and cancer survivorship as it could represent a unique opportunity to provide valuable reliable data on PA treatment trials and at population level throughout the survivorship continuum. As such, this study provides research and practical recommendations that will facilitate the researchers’ and clinicians’ decision to use and interpret the GSLTPAQ among cancer survivor populations.

## Methods

This systematic review and reporting of results were realized in reference to the Preferred Reporting Items for Systematic Review and Meta-Analyses (PRISMA Statement; [33]).

### Search strategies and study selection

One reviewer (SA) identified published English-written articles by searching six electronic databases and scanning reference list of included articles. Additionally, a cited reference search was performed using Scopus and Web of Science in order to screen for articles that cite the primary articles for the GSLTPAQ (i.e., [11, 22]). The period covered by the literature review was from January 1st 1985 (date the GSLTPAQ was published) to December 31st 2014. Articles were restricted to those that both assessed LTPA using the original or a modified version of the GSLTPAQ and cited Godin & Shephard [11] or Godin et al. [22], and (iii) were conducted among cancer survivors. For the purpose of this study, an individual is considered a cancer survivor from the time of the diagnosis until the end of his or her life [34]. Full details of the information source and search strategy, study selection, data collection, and extraction processes can be obtained from Additional file 1.

### GSLTPAQ’s measurement purposes

Information concerning the use of the measure and purpose (s) was retained [35, 36]. Specifically, the scale may have been used for ranking and/or classification purposes. PA may be assessed using the GSLTPAQ for a number of reasons such as examining the association between PA and health outcomes, identifying correlates/determinants of PA behavior, adjusting the association between two variables by controlling in the analyses for PA, reporting and describing PA prevalence within a given population, screening participants to determine eligibility for an intervention, evaluating the effectiveness of an intervention, and documenting within or between individuals changes in PA levels over time.

### GSLTPAQ’s item content and scoring methods

The GSLTPAQ intended scoring is the LSI, which is obtained using the following formula: (frequency of mild × 3) + (frequency of moderate × 5) + (frequency of strenuous × 9). The intended cut-point values for the classification scoring are based on the North American public health PA guidelines, that are defined as follows: individuals reporting moderate-to-strenuous LSI ≥ 24 are classified as *active* whereas individuals reporting moderate-to-strenuous LSI ≤ 23 are classified as *insufficiently active* (estimated energy expenditure < 14 Kcal/kg/week) [12]. In order to evaluate the variations in the use of the GSLTPAQ, information concerning the item content (frequency items only vs. frequency and duration items), the recall period (typical or last week vs. other recall timeframes), and scoring methods (LSI, frequency/week, minutes/week, METs × hours/week, percentage meeting PA guideline, and other measurement units) were retrieved.

### Validity evidence for the use of the GSLTPAQ among cancer survivors

Validity evidence for the use of the original version of the GSLTPAQ among cancer survivors was retrieved. First, effect sizes [e.g., Pearson’s or Spearman’s correlation coefficient (*r*)] based on the relation between GSLTPAQ scores and other device-based PA measures (e.g., accelerometer, pedometer) reported in the reviewed articles were identified as convergent validity evidence [16, 37]. Second, effect sizes assessing change in the LSI were calculated. Specifically, intervention studies that randomly assigned cancer survivors to receive either a supervised and prescribed PA training program (exercise group) or a placebo/non-PA intervention (control group) were examined [38]. Within this study design, it is expected that cancer survivors participating in a supervised and prescribed PA training program would report a greater increase in LSI than cancer survivors who were not training. For both the exercise and control group, effect size (i.e., Cohen’s *d*) reflecting the average group mean change in LSI was calculated. Additional methodological criteria were extracted to assess the risk of bias of these studies [i.e., sequence generation (reporting both the method and type of randomization); blinding of study assessors to participant assignment; adequate adherence rate (≥75 %) to the PA intervention; baseline imbalance for LSI; incomplete data for LSI (intention-to-treat analysis used, attrition rate, description of withdrawals and dropouts, and strategies used for handling missing data are reported and appropriated)].

### Data extraction

Information concerning the first and second objective was extracted by one reviewer (SA). A second reviewer (JL) independently extracted data from a random sample of the included articles (*N*_articles_ = 35). An inter-rater reliability value for each item was examined and discrepancies were resolved by consensus between SA and JL. Intraclass coefficient for study sample characteristics ranged from .98 to 1.00 and kappa coefficient ranged from .03 to .79 for study design and GSLTPAQ-related items. After discussion between reviewers, items for which kappa coefficient was unsatisfactory (< .41) were extracted again from all the included articles by one of the reviewers (SA) and corrections were made as needed. All information concerning the third objective was independently extracted by two reviewers (SA and JL). Both reviewers independently evaluated the methodological quality and retrieved statistical information for all relevant studies. Discrepancies were resolved by consensus between SA and JL.

### Data analysis

For the first and second objectives, information was summarized using descriptive statistics (i.e., frequencies, percentage) using SAS version 9.3 (SAS Institute, Cary, NC, USA). For the third objective, validity estimates based on the relation between LSI and device-based PA measures were reported as correlation coefficients and corresponding 95 % confidence interval [95 % CI]. The coefficients were then classified based on van Poppel et al. [37] criteria. A correlation coefficient for the GSLTPAQ-pedometer association ≥ .30 indicates the lowest level of evidence (level 3) because pedometer assesses walking behavior and may not capture the entire range of LTPA that cancer survivors participate in daily. A correlation coefficient for the GSLTPAQ-accelerometer association between .40 and .49, and ≥ .50 indicate medium (level 2) and high (level 1) level of evidence, respectively. Any validity estimates < .30 (for pedometer) and < .40 (for accelerometer) were considered as trivial and a (−) score was given (unsatisfactory evidence). Based on Mendoza, Stafford, and Stauffer [39]’s simulation study, a sample of at least 100 is recommended for obtaining precise reliability and validity estimates. Therefore, if the sample size was < 100, a (?) score was given (uncertain evidence), whereas a (+) score was given if this sample size was ≥ 100 (satisfactory evidence).

Effect sizes for sensitivity to change validity evidence were obtained using the following formula: (mean post-intervention LSI – mean baseline LSI)/standard deviation of baseline LSI [38]. As there are few effect sizes reported for sensitivity to change validity evidence, they were qualitatively appraised according to Cohen’s *d* criteria [40]: (*d* ≤ .20) trivial; (*d* = .20) small; (*d* = .50) medium; (*d* = .80) large. Depending of the PA dose prescribed (≤2 PA sessions of at least moderate intensity/week *vs*. ≥ 3 PA sessions or at least moderate intensity/week), satisfactory validity evidence was obtained if the validity estimate for the exercise group is medium (≤2 PA session/week) or large (≥3 PA session/week), while it is trivial/small for the control group. Again, if the sample size was < 100, a (?) score will be given (uncertain evidence).

## Results

### Literature search

**Fig. 1:**
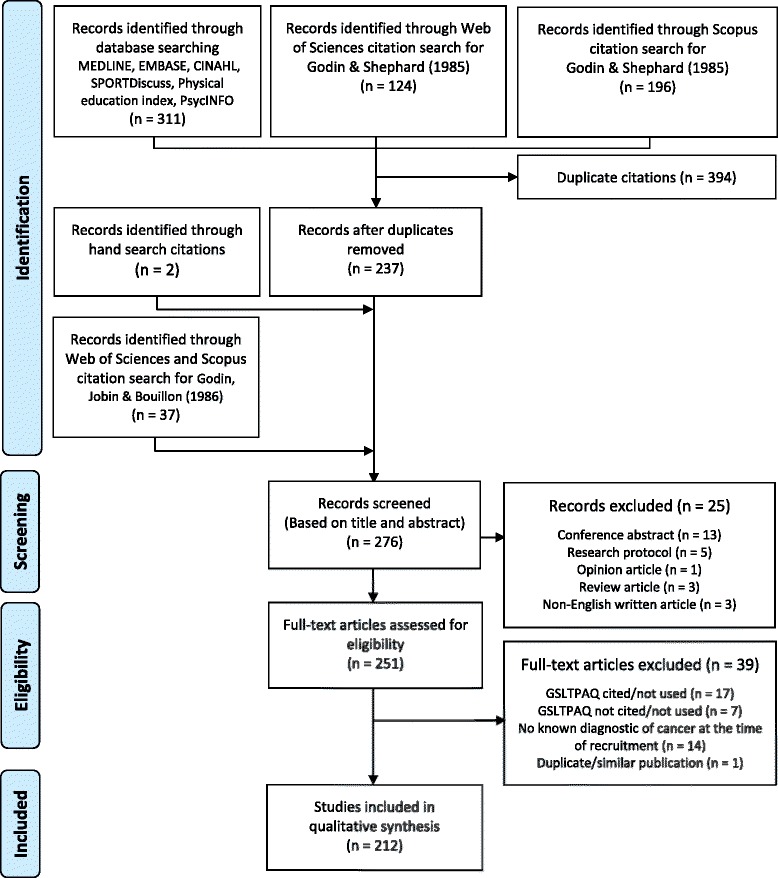
Flow Diagram. Adapted from: Moher D, Liberati A, Tetzlaff J, Altman DG, The PRISMA Group [33]. Fig. 1 depicts the number of studies screened, assessed for eligibility, and included in the review, with reasons for exclusions at each stage.

**Fig. 2:**
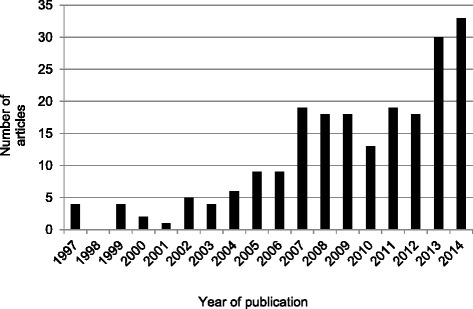
Number of Articles Reporting Using the Godin-Shephard Leisure-Time Physical activity Questionnaire in Cancer-Related Research (1997–2014)

The detailed process used to select studies is depicted in Fig. 1. There were 212 published English- language articles that reported using the GSLTPAQ to assess LTPA among cancer survivors between 1997 (the first evidence of the GSLTPAQ among cancer survivors) and 2014. Fig. 2 shows an increasing trend in the use of the questionnaire over time. As shown in Table 1, breast cancer survivors were the most frequently studied cancer population. In addition, 12 articles (5.7 %) targeted youth and adolescent cancer survivors (sample mean age < 18 years). A detailed summary of the characteristics of each study included in the review is presented in the Additional file 2.

**Table 1:** Characteristics of the articles included in the systematic review (*k* = 212)

Characteristics	Median [IQR]	Range (min - max)
Year of publication (year)	2010 [2007–2013]	1997 - 2014
Sample size (*N*)^a^	129 [56–359]	1 - 9105
Sample mean age (year)^a^	58 [52–64]	11 - 77
Sample mean percentage of female (%)	61 [41–100]	0 - 100
	Number of articles (*k*)	Percentage (%)
Country where participants came from		
Canada	110	51.9
USA	65	30.7
Australia	11	5.2
UK	9	4.2
Other countries^b,c^	17	8.0
Cancer site		
Breast	61	28.8
Prostate	19	9.0
Colorectal	16	7.5
Lung	12	5.7
Hematological (leukemia, myeloma)	10	4.7
Hodgkin/non-Hodgkin lymphoma	8	3.8
Endometrial	7	3.3
Brain/glioma	7	3.3
Head/neck/oral cavity	5	2.4
Ovarian	5	2.4
Kidney	5	2.4
Bladder	3	1.4
Survivors from different types of cancer	54	25.5

The unit of observation is the published article. IQR: 25th-75th interquartile range. *k*: number of published articles included in the systematic review. ^a^ *k* = 211 due to missing information in one article; for all the other variables, *k* = 212. ^b^One study included participants from Canada and USA (*k* = 1; 0.5 %) and one study included participants from Australia and New Zealand (*k* = 1; 0.5 %). ^c^Other countries included New Zealand (*k* = 4; 1.9 %), Ireland (*k* = 3; 1.4 %), Norway (*k* = 3; 1.4 %), Taiwan (*k* = 3; 1.4 %), Spain (*k* = 2; 0.9 %) and Italy (*k* = 1; 0.5 %).

### GSLTPAQ measurement purpose

As displayed in Table 2, 70.3 % and 50.9 % of the articles reported using the GSLTPAQ for ranking and classification purpose, respectively; this information was undetectable for two studies (1.1 %) given the lack of information reported [41, 42]. Specifically, the GSLTPAQ was most frequently used (frequency ≥ 10 %) for identifying correlates/determinants of LTPA barriers, motivation towards LTPA or future LTPA behavior (32.1 %); examining the association between LTPA and health-related outcomes (e.g., quality of life, fatigue; 29.7 %); evaluating the effectiveness of an intervention (18.4 %), comparing baseline levels of LTPA for cancer survivors randomly allocated to one of the experimental conditions (13.2 %), reporting and describing PA prevalence (10.9 %); or evaluating changes in LTPA levels across the cancer experience (i.e., before diagnosis, during treatment, and after treatment; 10.4 %). It is worth noting that a single study may have used the questionnaire for more than one purpose.

**Table 2:** Use of the godin-shephard leisure-time physical activity in oncology research

	Item content (*n* = 206)^a^		Recall period (*n* = 162)^c^		Measurement units (*n* = 210)^e^	
	Frequency	Frequency and duration	Typical or last week	Other recall periods	Frequency or LSI	Other units
General purpose						
Ranking only	45 (21.8 %)	53 (25.7 %)	41 (25.3 %)	32 (19.8 %)	50 (23.8 %)	49 (23.3 %)
Classifying only	6 (2.9 %)	51 (24.8 %)	9 (5.6 %)	37 (22.8 %)	3 (1.4 %)	57 (27.1 %)
Ranking and classifying	6 (2.9 %)	45 (21.8 %)	7 (4.3 %)	36 (22.2 %)	8 (3.8 %)	43 (20.5 %)
Total	57 (27.7 %)^b^	149 (72.3 %)	57 (35.2 %)	105 (64.8 %)^d^	61 (29.1 %)	149 (71.0 %)

The unit of observation is the published article. ^a^Whether or not duration items were measured was undetermined for four studies owing to the lack of information reported in the reviewed articles. ^b^For the two studies for which the measurement purpose was undetermined, duration items were not assessed. ^c^The recall period of the questionnaire was undetermined for 50 studies owing to the lack of information reported in the reviewed articles. ^d^For the two studies for which the measurement purpose was undetermined, the recall period was ‘other’ for one study. ^e^The general measurement purpose was undetectable for two studies owing to the lack of information reported in the reviewed articles.

### GSLTPAQ item content and scoring system

Only 12.3 % of the articles reported using the GSLTPAQ as originally intended in terms of item content (i.e., asking three questions about the frequency of mild, moderate and strenuous LTPA), recall period (i.e., during a typical week or in the last week), and scoring methods (i.e., using either LSI, frequency score or both). Further details are provided in Table 2. In 81.1 % of the articles reviewed, a modified version of the GSLTPAQ was used. The most frequent modification (72.3 %) was the collection of information on the average duration (minutes/week) for mild, moderate and strenuous LTPA bouts. LTPA scores were either reported as the number of minutes/week, the number of METs × hours/week, or the percentage of cancer survivors classified as *active* (e.g., individuals reporting ≥ 150 min of moderate-to-strenuous LTPA/week), *insufficiently active* (e.g., individuals reporting < 150 min of moderate-to-strenuous LTPA/week) and *sedentary* (e.g., individuals reporting 0 min of moderate-to-strenuous LTPA/week). In addition, some researchers selected an arbitrary LSI cut-point (e.g., LSI ≥ 15; [43], LSI ≥ 16; [44], or LSI ≥ 27; [45, 46]; a frequency cut-point (≥5 bouts of moderate-to-strenuous LTPA/week; [47]), or a MET × hours/week cut-point (≥10 MET × hours/week; [48]) to classify cancer survivors as *active* and *insufficiently active*.

### GSLTPAQ validity evidence in cancer survivors

There was no study specifically designed to estimate the validity of the GSLTPAQ LSI or classification scoring system in cancer survivors. However, two studies reported a Pearson correlation coefficient between the LSI and accelerometer counts [49, 50] and another reported such correlation between the LSI and pedometer step counts [51]. These correlations are: .53 [.23; .95] (*N* = 33, breast cancer survivors [49]); .57 [.26; 1.00] (*N* = 28, leukemia survivors [50]); and .31 [.04; .60] (*N* = 51, breast cancer survivors [51]). Thus, there is one uncertain level 3 and two uncertain level 1 pieces of validity evidence based on the relationship between GSLTPAQ and device-based PA scores in cancer research.

Identified in this review, there were six RCTs (reported in seven articles) evaluating structured and prescribed exercise interventions of 4 to 12 weeks in duration on PA behavior [51-57]. The interventions included a mix of aerobic (using cycle ergometer, treadmill or rowing ergometer) and resistance strength training (using body weight, free weight or muscular fitness machine). The intensity prescribed varied from 55 % (mild intensity) to 85 % (moderate to vigorous intensity) of participants’ maximal heart rates, depending on their initial levels of physical fitness. The methodological quality and findings of the studies are summarised in Table 3. In five of the six studies, cancer survivors were prescribed at least two sessions of MVPA per week, and were also counselled to perform up to three additional home-based PA sessions. The computed effect size for change in LSI for the exercise group in all those five studies was large. In contrast, the computed effect size for change in LSI for the control group was trivial/small in four studies [51, 52, 54, 55]. In one small study, a large change in LSI was also computed for the cancer survivors of the control group [53]. For one study [57], prostate cancer survivors were only prescribed two sessions of resistance training (moderate intensity) per week for 12 weeks. Although the participants were encouraged to supplement their resistance exercise training with home-based aerobic sessions, there was no formal behavioral home-based intervention offered. The computed effect size for change in LSI was small-to-medium for the intervention group and trivial for the control group. Sample sizes were < 100 for five studies and 100 for another one. Therefore, five satisfactory (four that were deemed uncertain [+ ?]) and one unsatisfactory (deemed uncertain [− ?]) effect sizes assessing the relative change in LSI were computed among the RCTs.

**Table 3:** Sensitivity to change validity estimate for the godin-shephard leisure-time physical activity in oncology research

Study	Risk of bias					Sensitivity to change validity estimate	
	Sequence generation (*N*)	Blinding of outcome assessors	Adherence (≥75 %)	Baseline imbalance	Incomplete data	Pre-post change in LSI (exercice group)	Pre-post change in LSI (control group)
Bourke et al. [52]^a^	Low (50)	Low	Yes (95 %)	Low	Low	2.31	.24
Bourke et al. [53]^a^	Low (18)	High^b^	Yes (90 %)	Unclear^c^	High^d^	1.67	1.17
Bourke et al. [54]^a^	Low (100)	Low	Yes (88 %)	Low	Low	1.63	.30
Broderick et al. [55]	Low (43)	Low	Yes (78 %)	Unclear^c^	High^d^	1.35	-.14
Cormie et al. [57]^a^	Low (20)	Low	Yes (93 %)	Low	Low	.45	.10
Perna et al. [51]	Low (51)	Low	Yes (83 %)	Low	Low	1.88	.27

*LSI* Leisure score index. Validity estimates are reported as Cohen’s *d*. ^a^The recall period of the GSLTPAQ was not explicitly stated. ^b^Only data analysts were blind to group assignment.^c^Reported only for socio-demographic and medical variables; however, the analyses were adjusted for baseline LSI. ^d^Last observation carried forward was the strategies used to deal with missing data. ^d^Complete cases analysis was performed; two participants dropped-out (7 %)

## Discussion

This study documented the frequency of use of the GSLTPAQ for ranking and classification purposes, summarized the use of the GSLTPAQ based on item content and scoring methods, and evaluated the validity evidence supporting the use of the GSLTPAQ among cancer survivors. The aims of this study were achieved based on a systematic review of 212 English-written published articles that reported using the GSLTPAQ among cancer survivors between 1997 and 2014.

The questionnaire was frequently used for classifying cancer survivors into *active* and *insufficiently active* categories in spite of the fact that there was no standard classification system for the GSLTPAQ available before 2011 [12]. None of the retrieved articles used the scoring system suggested by Godin [12] for interpreting the GSLTPAQ LSI score. In most cases, investigators modified the content of the questionnaire and used number of minutes, used an arbitrary or a distribution-based (e.g., quartile of LSI) cut-point to create their own classification system. Arbitrary or distribution-based classification can be potentially misleading [58, 59]. For example, such classification might have resulted in creating groups of *insufficiently active* and *active* individuals even if most of the sample was *active*. Similarly, the term ‘sedentary’ might not be appropriate for describing cancer survivors reporting 0 min of moderate-to-strenuous LTPA [60]. Some cancer survivors classified as *sedentary* might have engaged in mild PA, whereas some of them classified as *active* might have been sedentary for a large proportion of their waking time (i.e., being an ‘active couch potato’ [61]). Therefore, without rationale for classification, both arbitrary and distribution-based classifications are liable to cut-point bias and prone to misleading interpretation [26, 62]. Based on the findings of the current review, researchers are encouraged to use the LSI for ranking purpose and the GSLTPAQ classification coding system [12] for classification purpose. One study conducted among healthy adults [63] and breast cancer survivors [64] now provide support for the use of the GSLTPAQ classification coding system at the group level.

Over 80 % of the published articles using the GSLTPAQ among cancer survivors did not use the original version of this questionnaire. This finding may not be surprising given Sternfeld and Goldman-Rosas’s [36] observations about researchers and practitioners making ‘small’ modifications to existing PA questionnaires. The most frequent modification was the collection of information on the average duration (in minutes) for mild, moderate, and strenuous LTPA (e.g., [65–69]). Despite this common alteration to the GSLTPAQ, none of the retrieved studies provided validity evidence supporting the use and interpretation of scores derived from the modified GSLTPAQ. Without such evidence it is unknown if this modification has a trivial or large impact on the validity of the questionnaire. One study published in 2015 reported ‘fair’ ranking (rank-order correlation coefficient = 0.51), but ‘poor’ agreement (intraclass correlation coefficient = 0.31) between reported minutes of MVPA assessed with a modified version of the GSLTPAQ and an accelerometer among prostate cancer survivors [70]. However, this modification lengthens the questionnaire and may add recall and calculation burden to respondents [14, 26, 29]. Moreover, as the number of modifications increases (i.e., asking about the average duration of LTPA, changing the recall period, or creating and interpreting a new scoring system) the integrity of the information may be questioned. Flexibility in methods used to assess and interpret the score obtained from the GSLTPAQ is a source of heterogeneity that may contribute to inconsistent results across studies. Standardization in the use and interpretation of the GSLTPAQ in oncology research is warranted.

No study was found with the primary objective to estimate the validity of the GSLTPAQ in cancer survivors. Nonetheless, three studies reported validity evidence based on the relationship between the LSI and accelerometer or step counts [49–51]. The identified correlation coefficients compare favorably to those obtained from previous systematic reviews [37, 71–73] and previous studies reporting on the association between LSI and accelerometer data [5, 23] conducted outside the oncology context. Taken together, a limited amount of validity evidence from oncology research tends to support the use of the GSLTPAQ and the interpretation of the LSI for ranking purpose among cancer survivors.

Overall, findings support the use of the GSLTPAQ and the interpretation of the LSI for assessing relative change in PA among cancer survivors. This is a key addition to the literature because validity evidence supporting the use of the GSLTPAQ for assessing changes in LTPA among cancer survivors is scarce [16, 37]. However, the scope of these findings is limited by the fact that effect sizes were mostly derived from the synthesis of small samples of cancer survivors (*N* ≤ 100). As a result, the computed effect sizes lacked precision, which reflect uncertainty [37, 39]. Furthermore, because validation was not the primary aim of any oncology studies reviewed, the summarized findings may be hampered by within-study selective reporting bias [74]. Hence, it is likely that significant validity estimates were more likely to be reported than non-significant ones. Given that there is initial evidence suggesting that the sensitivity to changes of the LSI might vary with the levels of PA habits [25], it is recommended to use the GSLTPAQ to assess changes in LTPA only among cancer survivors having weak PA habits (i.e., *inactive* adult cancer survivors; [75]). Nonetheless, the lack of evidence regarding the ability of the questionnaire to accurately assess changes in LTPA limits our confidence in studies assessing change in LTPA across the cancer trajectory (i.e., before diagnosis, during treatment, after treatment). Additional studies gathering sensitivity to change validity evidence among cancer survivors are warranted.

The practical recommendations and future avenues of research concerning the use of the GSLTPAQ among cancer survivors are summarized in Table 4. They are based on the available validity evidence, both within [49–57, 64] and outside [5, 11, 22–25, 63] of oncology research. Additional validation studies, conducted among sufficiently large sample (*N* ≥ 100) of cancer survivors are needed, especially in children, adolescents, and survivors of other types of cancer than breast cancer. Although one study suggests that social desirability has limited impact on LSI among college students [24], it is impossible to ascertain that this finding generalized to cancer survivors. Similarly, it would be relevant to assess whether or not examples of LTPA provided for each intensity category of the questionnaire are pertinent and understood by cancer survivors.

**Table 4:** Practical recommendations and avenues of research for the validation of the GSLTPAQ in oncology research

Most popular specific uses	Practical recommendations	Avenues of research- types of validity evidence needed^a^
Use as a risk or protective factor for predicting health-related outcomes.	Supported	Relation with other PA measures in cancer survivors
*Property of the measure required:* Produce valid ranking.		
Use as a measure of past behavior for predicting LTPA behavior, barriers or motivation.	Supported	Relation with other PA measures in cancer survivors
	Supported	Relation with other PA measures in cancer survivors
*Property of the measure required:* Produce valid ranking.		
Use as a behavioral outcome in studies aiming at identifying the determinants of LTPA behavior.		
*Property of the measure required*: Produce valid ranking.		
Use as behavioral outcome in studies evaluating the effectiveness of behavior change intervention to *increase* LTPA.	Supported	Behavioral stability among *initially inactive* cancer survivors; Relation with other PA measures in cancer survivors; Sensitivity to changes in LTPA among *initially inactive* cancer survivors.
*Property of the measure required*: Produce valid ranking; detect and quantify relative change in LTPA behavior.		
Use as behavioral outcome in studies evaluating the effectiveness of behavior change intervention to *maintain* LTPA.	Not supported	Behavioral stability among *initially active* cancer survivors; Relation with other PA measures among *initially active* cancer survivors; Sensitivity to changes in LTPA among *initially active* cancer survivors.
*Property of the measure required*: Produce valid ranking; detect and quantify relative change in LTPA behavior.		
Use as behavioral outcome for comparing baseline levels of LTPA of cancer survivors randomly allocated to one of the experimental conditions.	Supported	Relation with other PA measures in cancer survivors.
*Property of the measure required*: Produce valid ranking.		
Use as a behavioral outcome for evaluating LTPA behavior change across the cancer experience (i.e., before diagnosis, during treatment, and after treatment).	Not supported	Behavioral stability in cancer survivors; Relation with other PA measures in cancer survivors; Sensitivity to changes LTPA in cancer survivors.
*Property of the measure required*: Produce valid ranking; detect and quantify relative change in LTPA behavior.		
Use as a behavioral outcome for reporting and describing PA prevalence. *Property of the measure required*: Produce stable estimates of LTPA at the population level that are free of bias (i.e., accurately estimate LTPA levels)	Not supported	Behavioral stability in cancer survivors; Relation with other PA measures in cancer survivors (absolute interpretation).

^a^Based on the approach suggested by Masse and de Niet [16]. *LTPA* leisure-time physical activity, *PA* physical activity.

Although the recall time frame of the original GSLTPAQ is a ‘typical 7-day period’, it may also be appropriate to use the ‘past 7-day (i.e., the last week)’ recall timeframe depending on the measurement purpose and study design [36]. The ‘past 7-day (i.e., the last week)’ recall timeframe may be used provided that it is reasonably representative of the typical LTPA in cancer survivors. Moreover, the ‘past 7-day (i.e., the last week)’ recall timeframe is not appropriate in case–control study as the timeframe of the exposure (i.e., LTPA) does not precede the health outcome. Additional studies testing whether or not the recall period influences the quality of the GSLTPAQ for different specific purpose and study design are needed.

Lastly, the GSLTPAQ’s characteristic most likely associated with measurement error is misreporting the intensity of the activities (e.g., reporting a mild LTPA as moderate LTPA or reporting a moderate LTPA as strenuous LTPA), which likely occurs when the perceived and absolute intensity of a given LTPA are different [26, 29]. In this regard, asking about mild LTPA may reduce the misreporting of LTPA intensities [76]. Therefore, it is advisable to ask about mild LTPA, even if the investigators are only interested in moderate and strenuous LTPA.

### Limitations

This study has some limitations. Despite the fact that a thorough method was implemented to identify and select articles, only one reviewer screened citations for eligibility. In addition, there were inadequate details in articles regarding the recall period of the GSLTPAQ for 23.6 % of the reviewed studies. Although authors cited the original publication of the GSLTPAQ and appeared to have used the original version of this questionnaire, we could not ascertain that the recall period was either the ‘typical week’ or ‘last week’. No attempt was made to contact the authors of those articles to obtain clarifications on the recall period used. Lastly, our search strategy was restricted to English-language articles published in peer-review journals. We acknowledge that this may have resulted in an oversampling of small and moderate sample size studies reporting significant and large effect sizes. Although publication bias is suspected, especially for the findings related to the validity estimates of the GSLTPAQ, how much of an impact it might have had on review findings is unknown. It is worth noting that most of the samples in the articles reviewed included primarily North American female adult breast cancer survivors. Overall, participants were highly educated and volunteered to participate in a study. Thus, the results of this review may not be generalizable to other cancer survivor populations.

## Conclusion

This systematic review showed that the use of the GSLTPAQ for classification purpose in oncology research is common. Standardization in the use and interpretation of the GSLTPAQ in oncology research is warranted. Although limited, the current state of evidence tends to support the use of the original form of the GSLTPAQ and interpreting the LSI for ranking respondents from the lowest to highest levels of LTPA within a given sample of cancer survivors. Thus, the GSLTPAQ’s LSI may be used in cancer survivors’ studies for (*i*) identifying the correlates/determinants of LTPA behavior, (*ii*) verifying whether or not LTPA is a risk or a protective factor of relevant health-related outcomes, and (*iii*) evaluate the efficacy of LTPA behavior change interventions.

## Additional files

Additional file 1:
**Components of the Comprehensive Systematic Review Concerning the use of the Godin-Shephard Leisure-Time Physical Activity Questionnaire in Oncology Research.** Description of data: Reports full details of the information source and search strategy, study selection, data collection, and extraction processes. Additional file 2:
**Characteristics of the Sample for Cancer Survivors Published Article that Used the Godin-Shephard Leisure-Time Physical Activity.** Description of data: Reports a detailed summary of the characteristics of each study included in the review.

## Abbreviations

GSLTPAQ: Godin-Shephard Leisure-Time Physical Activity Questionnaire
LSI: Leisure score index
LTPA: Leisure-time physical activity
MET: Metabolic equivalent of task
MVPA: Moderate-to-vigorous physical activity
PA: Physical activity
