# Erratum to: The use of the Godin-Shephard Leisure-Time Physical Activity Questionnaire in oncology research: a systematic review

**DOI:** 10.1186/s12874-016-0131-5

**Published:** 2016-03-09

**Authors:** Steve Amireault, Gaston Godin, Jason Lacombe, Catherine M. Sabiston

**Affiliations:** Faculty of Kinesiology and Physical Education, Warren Stevens Building, University of Toronto, 55 Harbord St., Toronto, ON M5S 2W6 Canada; Department of Psychology, Faculty of Arts and Science, PY Building, Concordia University, 7141 Sherbrooke West, Montreal, QC H4B 1R6 Canada; Faculty of Nursing, Pavillon Ferdinand-Vandry, Université Laval, 1050 Avenue de la Médecine, Quebec City, QC G1V 0A4 Canada

## Erratum

After publication of the original article [1], it was noticed that authors’ affiliations were incorrectly specified. Affiliation 1 was incorrectly given as Purdue University, whereas the review was carried out at the University of Toronto.

The updated and correct author affiliations of the original article [1] are presented in this erratum. As a result, the ‘Authors’ information’ and Authors details’ sections also should have originally appeared as presented here.
