# Supplementary material for: The use of the Godin-Shephard Leisure-Time Physical Activity Questionnaire in oncology research: a systematic review
Source: BMC Med Res Methodol. 2015 Aug 12;15:60. doi: 10.1186/s12874-015-0045-7 (PMC4542103; doi:10.1186/s12874-015-0045-7)
Supplement: Additional file 1: — Components of the Comprehensive Systematic Review Concerning the use of the Godin-Shephard Leisure-Time Physical Activity Questionnaire in Oncology Research. Description of data: Reports full details of the information source and search strategy, study selection, data collection, and extraction processes. [file 12874_2015_45_MOESM1_ESM.doc]

**The use of the Godin-Shephard Leisure-Time Physical Activity Questionnaire in oncology research: A systematic review**

Steve Amireault^1,2^, Gaston Godin^3^, Jason Lacombe^1^ and Catherine M. Sabiston^1^

^1^Faculty of Kinesiology and Physical Education, University of Toronto, Toronto, Canada;

^2^Department of Psychology, Faculty of Arts and Science, Concordia University, Montreal, Canada;

^3^Faculty of Nursing, Université Laval, Quebec City, Canada.

Correspondence should be sent to Steve Amireault, University of Toronto, Faculty of Kinesiology and Physical Education, 55 Harbord Street, Warren Stevens Building, office 227, Toronto, ON, Canada, M5S 2W6. E-mail: [steve.amireault@utoronto.ca](mailto:steve.amireault@utoronto.ca).

**Additional File 1**: Components of the Comprehensive Systematic Review Concerning the use of the Godin-Shephard Leisure-Time Physical Activity Questionnaire in Oncology Research

**Eligibility criteria**

Studies using correlational (i.e., cross-sectional, longitudinal or retrospective) and experimental (one-group pre-test/post-test; quasi-experimental; truly experimental) designs were included in the review. Case-control studies were included, only if participants from the case group (i.e., cancer survivors) had a known diagnostic of cancer at the time of recruitment. In addition, study protocol, case study and systematic review/meta-analysis were excluded. Included studies will be restricted to:

1. Studies that both assessed LTPA using the original or a modified version of the Godin Shephard Leisure-Time Physical Activity Questionnaire (GSLTPAQ) and;
2. Cited Godin & Shephard [1] or Godin, Jobin, & Bouillon [2] and;
3. Studies including cancer survivors. According to the National Cancer Institute [3], an individual is considered a cancer survivor from the time of diagnosis until the end of his or her life. Cancer survivors of any age were considered.

**Information sources and search strategy**

Articles were identified by searching six electronic databases and scanning reference list of the included articles. A reviewer (SA) performed the search in June 2013, which was updated in March 2014 and in March 2015. The electronic databases search strategy was developed for MEDLINE (via Pubmed; 1966 +) and adapted for CINAHL (1982 +), EMBASE (1974 +), SPORTDiscus (1975 +), Physical education index (1978 +), and PsycINFO (1994 +). For all databases, the following search terms were used to search in title and abstract: (“Godin leisure time questionnaire” OR “Godin leisure time exercise questionnaire” OR “Godin leisure time index” OR “Leisure score index”) AND (cancer OR neoplasm* OR tumor). In addition, cancer-related index terms (e.g., MeSH terms, Emtree terms, SPORTDiscus thesaurus, etc.), specific to each database, were used. Full details of the electronic search, including limitations and specific research fields, for all databases are presented in Table 1 to Table 6. Additionally, we performed a cited reference search using Web of Science and Scopus in order to screen for articles that cite Godin and Shephard [1] and Godin et al. [2]. Books, book sections, and conference abstracts were excluded.

**Study selection**

All references retrieved were exported into EndNote software. Title and abstract were screened by one reviewer (SA) and clearly irrelevant articles were excluded. The full-text published papers of the remaining potentially relevant citations were retrieved. Then, a reviewer (SA) independently performed eligibility assessment for all full text articles obtained. Although the unit of observation was the published article, included articles from the same author (or group of authors) were scrutinized to avoid counting double citations. Specifically, year of publication, the number of study participants (*N*), the study participant characteristics, study objectives and the primary outcomes results were verified.

**Data collection and extraction processes**

We developed a data extraction sheet that was independently pilot tested by two reviewers (SA and JL) on five randomly selected citations. Information concerning the first and second objective of the systematic review was extracted by one reviewer (SA). A second reviewer (JL) independently extracted data from a random sample of the included articles (*N*_articles_ = 35). These citations were randomly selected using a computer-generated random number table. An inter-rater reliability value for each item was examined and discrepancies were resolved by discussion between two reviewers (SA and JL). All information concerning the third objective of the systematic review was independently extracted by two reviewers (SA and JL). They evaluated the methodological quality and retrieved validity estimate for all relevant studies. Discrepancies were resolved by consensus between SA and JL.

**Data extracted and definitions**

Necessary information to present Study Characteristics (SC) and Result of Individual Studies (RIS) were extracted from each included citations. The Table 7 specifies which information was extracted.

Table 8 presents the inter-rater reliability indicators for study characteristics, GSLTPAQ item content, general and specific measurement purposes, and measurement units. Inter-rater reliability indicators were almost perfect for all study characteristics. However, some inter-rater reliability indicators were not satisfactory (see bolded items in Table 8). All, discrepancies between reviewers were resolved by discussion. After discussion between reviewers, data were extracted again for all items for which the inter-rater reliability indicators were deemed unsatisfactory (kappa coefficient < .41) by a reviewer (SA). As needed, corrections were made.

**References**

1. Godin G, Shephard RJ: **A simple method to assess exercise behavior in the community.** *Can J Appl Sport Sci* 1985, **10**:141-146.

2. Godin G, Jobin J, Bouillon J: **Assessment of leisure time exercise behavior by self-report: A concurrent validity study.** *Can J Public Health* 1986, **77**:359-362.

3. National Cancer Institute at the National Institutes of Health: **National Cancer Institute Dictionary of Cancer Terms** [<http://www.cancer.gov/dictionary?cdrid=445089>]

Table 1. Documenting the Search- MEDLINE via Pubmed

| Name of the database (range of dates) | MEDLINE (Pubmed:1966 +) |
| --- | --- |
| Date of the search | June 14^th^ 2013; updated March 10^th^ 2014 and March 25^th^ 2015 |
| Initials of the person who ran the search | SA |
| Limits :  Publication date: 1985 up to December 31, 2013  Search fields: All fields, MeSH terms | Search and MeSH terms:  S1- Godin leisure time questionnaire  S2- Godin leisure time exercise questionnaire  S3- Godin leisure-time index  S4- Leisure score index  S5- S1 or S2 or S3 or S4  S6- cancer  S7- neoplasm*  S8- tumor  S9- neoplasms (MeSH term)  S10- S6 or S7 or S8 or S9  S11- S5 and S10 |
| Number of hits | 89 |

Table 2. Documenting the Search- Embase

| Name of the database (range of dates) | Embase and Medline via Embase (1974 +) |
| --- | --- |
| Date of the search | June 14^th^ 2013; updated March 10^th^ 2014 and March 25^th^ 2015 |
| Initials of the person who ran the search | SA |
| Limits :  Publication date: 1985 up to December 31, 2013  Search fields: All fields, Emtree terms | Search and Emtree terms:  S1- Godin leisure time questionnaire  S2- Godin leisure time exercise questionnaire  S3- Godin leisure-time index  S4- Leisure score index  S5- S1 or S2 or S3 or S4  S6- cancer  S7- neoplasm*  S8- tumor  S9- neoplasms/exp (Emtree term)  S10- S6 or S7 or S8 or S9  S11- S5 and S10 |
| Number of hits | 46 |

Table 3. Documenting the Search- CINAHL

| Name of the database (range of dates) | CINAHL (1982 +) |
| --- | --- |
| Date of the search | June 14^th^ 2013; updated March 10^th^ 2014 and March 25^th^ 2015 |
| Initials of the person who ran the search | SA |
| Limits :  Publication date: 1985 up to December 31, 2013  Search fields: All text, CINAHL Headings | Search terms and CINAHL Headings:  S1- “Godin leisure time questionnaire”- all text  S2- “Godin leisure time exercise questionnaire”  S3- “Godin leisure time index”  S4- “Leisure score index”  S5- S1 or S2 or S3 or S4  S6- cancer – title, abstract  S7- neoplasm*  S8- tumor  S9- neoplasms (CINAHL Headings)  S10- S6 or S7 or S8 or S9  S11- S5 and S10 |
| Number of hits | 65 |

Table 4. Documenting the Search- SPORTDiscus

| Name of the database (range of dates) | SPORTDiscus (1975+) |
| --- | --- |
| Date of the search | June 14^th^ 2013; updated March 10^th^ 2014 and March 25^th^ 2015 |
| Initials of the person who ran the search | SA |
| Limits :  Publication date: 1985 up to December 31, 2013  Search fields: Title, abstract, keywords, SPORTDiscus thesaurus | Search terms and SPORTDiscus thesaurus:  S1- “Godin leisure time questionnaire”- all text  S2- “Godin leisure time exercise questionnaire” – all text  S3- “Godin leisure-time index” – all text  S4- “Leisure score index”- all text  S5- S1 or S2 or S3 or S4  S6- cancer –title, keywords, abstract  S7- neoplasm*  S8- tumor  S9- cancer (SPORTDiscus thesaurus)  S10- cancer-patients (SPORTDiscus thesaurus)    S11- S6 or S7 or S8 or S9 or S10  S12- S5 and S11 |
| Number of hits | 18 |

Table 5. Documenting the Search- PsycINFO

| Name of the database (range of dates) | PsycINFO (1994 +) |
| --- | --- |
| Date of the search | June 14^th^ 2013; updated March 10^th^ 2014 and March 25^th^ 2015 |
| Initials of the person who ran the search | SA |
| Limits :  Publication date: 1985 up to December 31, 2013  Search fields: All fields, index terms (keywords), Author cited | Search terms, author cited and index terms:  S1- “Godin leisure time questionnaire”  S2- “Godin leisure time exercise questionnaire”  S3- “Godin leisure-time index”  S4- “Leisure score index”  S5- S1 or S2 or S3 or S4  S6- cancer  S7- neoplasm*  S8- tumor  S9- neoplasms (index terms)  S10- S6 or S7 or S8 or S9  S11- S5 and S10 |
| Number of hits | 86 |

Table 6. Documenting the Search- Physical Education Index

| Name of the database (range of dates) | Physical Education Index (1978 +) |
| --- | --- |
| Date of the search | June 14^th^ 2013; updated March 10^th^ 2014 and March 25^th^ 2014 |
| Initials of the person who ran the search | SA |
| Limits :  Publication date: 1985 up to December 31, 2013  Search fields: All fields, identifier (keywords) | Search terms, author cited and identifier (keywords):  S1- “Godin leisure time questionnaire”  S2- “Godin leisure time exercise questionnaire”  S3- “Godin leisure-time index”  S4- “Leisure score index”  S5- S1 or S2 or S3 or S4  S6- cancer  S7- neoplasm  S8- tumor  S9- cancer (keywords)  S10- S6 or S7 or S8 or S9  S11- S5 and S10 |
| Number of hits | 7 |

**Table 7. Items Extracted**

| Items extracted | Targeted use |
| --- | --- |
| 1-Author/ year of publication  2-Country where the study participants came from  3-Descriptive characteristics of the samples (mean age; % of female; level of education: % of individuals who obtained a first degree university diploma; and type of cancer)  4- Sample size (*N*)  5- Study design (correlational- transversal; correlational- longitudinal; case-control; pre-experimental; quasi-experimental; truly-experimental)  6- How the GSLTPAQ was assessed? (item contents, recall period)  7- For what purpose the GSLTPAQ was used? (ranking, classification or both)  8- Measurement unit of the GSLTPAQ score(s) used  9- The name of the variable linked with the GSLTPAQ | SC  SC  SC  SC  SC  RIS  RIS  RIS  RIS |

*Note*. GSLTPAQ: Godin-Shephard Leisure-Time Physical Activity Questionnaire. SC: study characteristics. RIS: result of individual studies.

Table 8. Inter-Raters Reliability Indicators (*k* = 35; *j* = 2)

| Items | Reliability coefficient | |
| --- | --- | --- |
|  | Intraclass Coefficient [95%CI] | |
| Years of publication | 1 | |
| *N* | .99 [.98, .99] | |
| Sample mean age | 1 | |
| % female | .98 [.96, .99] | |
|  | Kappa [95%CI] | % of agreement |
| Study design | .69 [.49, .88] | 69% |
| **General measurement purpose** | .36 [.11, .61] | 57% |
| Specific measurement purpose  **Predicting health-related outcomes**  **Correlates of PA**  Outcome of a behaviour change intervention  **Prevalence of PA**  Change in PA over time | .38 [.08, .67]  .40 [.06, .75]  -.06^1^  .29 [.00, .67]  .70 [.43, .97] | 69%  77%  89%  77%  89% |
| Item content  Collecting information on average duration  **Recall period** | .58 [.17, .92]  .30 [.00, .61] | 89%  40% |
| Measurement units  LSI  **Frequency/week**  Minutes/week  METs × hours/week  **% meeting PA guidelines** | .55 [.17, .92]  .03 [.00, .08]  .79 [.56, 1.00]  .60 [.27, .94]  .32 [.02, .61] | 89%  34%  91%  89%  69% |

*Note*. *k* = number of studies. *j* = number of raters/judges. Inter-rater reliability for continuous/interval variables was assessed with intraclass coefficients (ICC) with its 95% confidence interval [95%CI]. Inter-rater reliability for categorical variables was assessed using kappa or weighted kappa coefficient with its 95% confidence interval [95%CI]. ^1^Negative values of kappa indicate agreement that is less than chance alone would predict.
