# Supplementary material for: The use of the Godin-Shephard Leisure-Time Physical Activity Questionnaire in oncology research: a systematic review
Source: BMC Med Res Methodol. 2015 Aug 12;15:60. doi: 10.1186/s12874-015-0045-7 (PMC4542103; doi:10.1186/s12874-015-0045-7)
Supplement: Additional file 2: — Characteristics of the Sample for Cancer Survivors Published Article that Used the Godin-Shephard Leisure-Time Physical Activity. Description of data: Reports a detailed summary of the characteristics of each study included in the review. [file 12874_2015_45_MOESM2_ESM.docx]

**The use of the Godin-Shephard Leisure-Time Physical Activity Questionnaire in oncology research: A systematic review**

Steve Amireault^1,2^, Gaston Godin^3^, Jason Lacombe^1^, & Catherine M. Sabiston^1^

^1^Faculty of Kinesiology and Physical Education, University of Toronto, Toronto, Canada;

^2^Department of Psychology, Faculty of Arts and Science, Concordia University, Montreal, Canada;

^3^Faculty of Nursing, Université Laval, Quebec City, Canada.

Correspondence should be sent to Steve Amireault, University of Toronto, Faculty of Kinesiology and Physical Education, 55 Harbord Street, Warren Stevens Building, office 227, Toronto, ON, Canada, M5S 2W6. E-mail: [steve.amireault@utoronto.ca](mailto:steve.amireault@utoronto.ca).

Additional File 2: Characteristics of the Sample for Cancer Survivors Published Article that Used

the Godin-Shephard Leisure-Time Physical Activity

| Author (year) | Baseline sample characteristics | Study design | GSLTPAQ item content | LTPA outcomes | Specific measurement purpose | General measurement purpose | Variables linked with LTPA^1^ |
| --- | --- | --- | --- | --- | --- | --- | --- |
| SMH Alibhai, S O'Neill, K Fisher-Schlombs, H Breunis, JM Brandwein, N Timilshina, GA Tomlinson, HD Klepin and SN Culos-Reed [1] | *N* = 35- Canada  Mean age: 56.4  54.3% ♀  57.1% beyond high school education  Type of cancer:  Leukemia | Pre-experimental | **Recall period**:  Not specified  **Asking for**:  Frequency and duration | **Scoring algorithm**:  Not specified- Assessment of moderate and strenuous LTPA  **Measurement unit**:  METs-hour/week | Assessment of baseline equivalence of the age groups | Relative ranking | Age |
| SMH Alibhai, S O'Neill, K Fisher-Schlombs, H Breunis, N Timilshina, JM Brandwein, MD Minden, GA Tomlinson and SN Culos-Reed [2] | *N* = 38- Canada  Mean age: 56.1  55.3% ♀  73.7% beyond high school education  Type of cancer:  Leukemia | Truly experimental | **Recall period**:  Typical week  **Asking for**:  Frequency and duration | **Scoring algorithm**:  Frequency × duration moderate + frequency × duration of strenuous  **Measurement unit**: Minutes/week  **Scoring algorithm**:  Meeting PA recommendations:  *Active*: ≥ 150 minutes of MVPA;  *Insufficiently active*: < 150 minutes of MVPA.  **Measurement unit**:  Proportion (%) of *active* and *insufficiently active* individuals | Assessment of intervention adherence  Assessment of baseline equivalence of the treatment and comparison groups | Relative ranking  Classification | Experimental conditions |
| MA Andrykowski, AO Beacham and PB Jacobsen [3] | *N* = 257- USA  Mean age: 55.2  100% ♀  71.5% beyond high school education  Type of cancer:  Breast | Correlational- longitudinal | **Recall period**:  *Baseline*-  Last 6 months prior to cancer diagnosis;  *Other measurement waves*-  Last 7 days  **Asking for**:  Frequency and duration | **Scoring algorithm**:  Frequency × duration of mild/15 × 3 + frequency × duration of moderate/15 × 5 + frequency × duration of strenuous/15 × 9  Frequency × duration of mild + frequency × duration of moderate + frequency × duration of strenuous  Meeting PA recommendations:  *Active*: ≥ 150 minutes of MVPA/week; i*nsufficiently active*: ≤ 150 minutes of MVPA/week; *sedentary*: not reporting any frequency of mild, moderate and strenuous LTPA (score = 0).  **Measurement unit**:  METs/week  Minutes/week    Proportion (%) of *active*, *insufficiently active* and *sedentary* individuals | Identifying correlates of LTPA  Assessment of LTPA patterns during cancer experience  Assessment of baseline equivalence of the treatment and comparison groups | Relative ranking  Classification | Cancer-related/medical variables  Intensity of cancer treatment  Time |
| H Badr, J Chandra, RJ Paxton, JL Ater, D Urbauer, CS Cruz and W Demark-Wahnefried [4] | *N* = 170- USA  Mean age: 18  48% ♀  73% had at least some college education  Type of cancer:  Leukemia, lymphoma, sarcoma, CNS cancer | Correlational- transversal | **Recall period**:  Not specified  **Asking for**:  Frequency | **Scoring algorithm**:  Frequency of moderate × 5 × 4 + frequency of strenuous × 9 × 4  **Measurement unit**:  Minutes/week | Examine the association between PA and health-related outcomes  Identify correlates of PA  Investigate exercise programming and counselling preferences | Relative ranking | Body mass index  Cancer worry  Quality of life  Clinical/medical variables  Socio-demographic variables  Exercise programming and exercise counselling preferences |
| H Badr, RJ Paxton, JL Ater, D Urbauer and W Demark-Wahnefried [5] | *N* = 35- USA  Mean age: 18  48% ♀  Type of cancer:  Leukemia, lymphoma, sarcoma, CNS cancer | Correlational- longitudinal | **Recall period**:  Last 7 days  **Asking for**:  Frequency and duration | **Scoring algorithm**:  Frequency × duration of moderate + frequency × duration of strenuous  **Measurement unit**:  Minutes/week | Examine the difference in MVPA between child cancer survivors and their parents | Relative ranking | Types of participants (child vs. their parents) |
| MK Baldwin and KS Courneya [6] | *N* = 64- Canada  Mean age: 51  100% ♀  64% beyond college/university education  Type of cancer:  Breast | Correlational- transversal | **Recall period**:  Typical week  **Asking for**:  Frequency | **Scoring algorithm**:  Frequency of mild × 3 + frequency of moderate × 5 + frequency of strenuous × 9  **Measurement unit**:  LSI (arbitrary units) | Examine the association between PA and health-related outcomes | Relative ranking | Physical competence  Physical acceptance  Self-esteem |
| LJ Bélanger, RC Plotnikoff, A Clark and KS Courneya [7] | *N* = 588- Canada  Mean age: 38.2  70.4% ♀  61.9% beyond high school education  Type of cancer:  Breast, thyroid, lymphoma | Correlational- transversal | **Recall period**:  Not specified  **Asking for**:  Frequency and duration | **Scoring algorithm**:  Meeting PA recommendations*:  *Above guidelines*: ≥ 300 minutes of MVPA;  *Within guidelines*: 150-300 minutes of MVPA; *Insufficiently active*: Some but < 150 minutes of MVPA;  *Completely sedentary*: No moderate-to-strenuous LTPA (score = 0)  *Minutes of strenuous LTPA were multiplied by 2  **Measurement unit**:  Proportion (%) of individuals within each PA categories: *Above guidelines, within guidelines, insufficiently active, and completely sedentary*. | Assess PA prevalence  Examine the association between PA and health-related outcomes | Classification | Clinical/medical variables  Depression  Quality of life  Stress |
| LJ Bélanger, RC Plotnikoff, AM Clark and KS Courneya [8] | *N* = 588- Canada  Mean age: 38.2  70.4% ♀  61.9% beyond high school education  Type of cancer:  Breast, lymphoma, thyroid. | Correlational- transversal | **Recall period**:  Not specified  **Asking for**:  Frequency and duration | **Scoring algorithm**:  Meeting PA recommendations:  *Above guidelines*: ≥ 300 minutes of MVPA;  *Within guidelines*: 150-300 minutes of MVPA; *Insufficiently active*: Some but < 150 minutes of MVPA;  *Completely sedentary*: No moderate/strenuous LTPA (score = 0)  **Measurement unit**:  Proportion (%) of individuals within each PA categories: *above guidelines*, *within guidelines*, *insufficiently active*, and completely *sedentary.* | Identify correlates of LTPA | Classification | Cancer-related/medical  Socio-demographic variables  Theory of planned behavior constructs |
| LJ Belanger, WK Mummery, AM Clark and KS Courneya [9] | *N* = 212- Canada  Median age: 35  60.8% ♀  67.5% completed university/college  Type of cancer:  Breast, lymphoma, thyroid, testicular | Truly experimental | **Recall period**:  Past month  **Asking for**:  Frequency and duration | **Scoring algorithm**:  Frequency × duration of moderate + frequency × duration of strenuous × 2  Frequency × duration of moderate  Frequency × duration of strenuous  **Measurement unit**: Minutes/week  **Scoring algorithm**:  Meeting PA recommendations:  *Active*: ≥ 150 minutes of MVPA or ≥ 75 minutes of vigorous PA;  *Insufficiently active*: < 150 minutes of MVPA and < 75 minutes of vigorous PA  **Measurement unit**:  Proportion (%) of *active* and *insufficiently active* individuals | Assessment of baseline equivalence of the treatment and comparison groups  Outcome of a PA intervention | Relative ranking  Classification | Experimental conditions |
| L Bellury, L Ellington, SL Beck, MA Pett, J Clark and K Stein [10] | *N* = 184- USA  Mean age: 77  100% ♀  41% beyond high school education  Type of cancer:  Breast | Correlational- transversal | **Recall period**:  Not specified  **Asking for**:  Frequency | **Scoring algorithm**:  Frequency of mild × 3 + frequency of moderate × 5 + frequency of strenuous × 9  **Measurement unit**:  LSI (arbitrary units) | Examine the association between PA and health-related outcomes | Relative ranking | Physical functioning |
| CM Blanchard, KS Courneya, WM Rodgers and DM Murnaghan [11] | *N* = 129- Canada  Mean age: 64  64% ♀  50% beyond high school education  Type of cancer:  Breast and prostate | Correlational- transversal | **Recall period**:  Last week  **Asking for**:  Frequency | **Scoring algorithm**:  Frequency of mild + frequency of moderate + frequency of strenuous  **Measurement unit**:  Number of ≥ 15 minutes LTPA cessions (frequency)/week | Identify correlates of LTPA | Relative ranking | Theory of planned behavior constructs |
| CM Blanchard, KS Courneya and K Stein [12] | *N* = 9105- USA  Mean age: 67.4  55% ♀  31% completed college/graduate education  Type of cancer:  Breast, colorectal, bladder, skin melanoma, uterine | Correlational- transversal | **Recall period**:  Last 3 months  **Asking for**:  Frequency and duration | **Scoring algorithm**:  Meeting PA recommendations:  *Active*: ≥ 150 minutes of MVPA or ≥ 60 minutes of vigorous PA;  *Insufficiently active*: < 150 minutes of MVPA and < 60 minutes of vigorous PA  **Measurement unit**:  Proportion (%) of *active* and *insufficiently active* individuals | Assess the prevalence of PA  Examine the association between PA and health-related outcomes | Classification | Quality of life |
| CM Blanchard, K Stein and KS Courneya [13] | *N* = 3241- USA  Mean age: 67.4  55% ♀  Type of cancer:  Breast, colorectal, bladder, skin melanoma, uterine | Correlational- transversal | **Recall period**:  Last 3 months  **Asking for**:  Frequency and duration | **Scoring algorithm**:  Meeting PA recommendations:  *Active*: ≥ 150 minutes of MVPA or ≥ 60 minutes of vigorous PA;  *Insufficiently active*: < 150 minutes of MVPA and < 60 minutes of vigorous PA  **Measurement unit**:  Proportion (%) of *active* and *insufficiently active* individuals | Examine the association between PA and health-related outcomes  Identify correlates of PA | Relative ranking | Quality of life  Body mass index |
| JM Blaney, A Lowe-Strong, J Rankin-Watt, A Campbell and JH Gracey [14] | *N* = 454- Ireland  Median age: 61  76% ♀  Type of cancer:  Breast, prostate, throat | Correlational- transversal | **Recall period**:  Not specified  **Asking for**:  Frequency | **Scoring algorithm**:  Frequency of mild; frequency of moderate; frequency of strenuous.  **Measurement unit**:  Number of ≥ 15 minutes LTPA cessions (frequency)/week | Describe study sample in terms of LTPA | Relative ranking | N/A |
| K Bolam, B Beck, K Adlard, T Skinner, P Cormie, D Galvão, N Spry, R Newton and D Taaffe [15] | *N* = 69- Australia  Mean age: 71  0% ♀  Type of cancer:  Prostate | Correlational- transversal | **Recall period**:  Not specified  **Asking for**:  Frequency and duration | **Scoring algorithm**:  Frequency × duration of mild + frequency × duration of moderate + frequency × duration of strenuous  Frequency × duration of moderate + frequency × duration of strenuous  **Measurement unit**:  Minutes/week | Describe study sample in terms of LTPA | Relative ranking | N/A |
| L Bourke, H Doll, H Crank, A Daley, D Rosario and JM Saxton [16] | *N* = 50- UK  Median age: 72  0% ♀  Type of cancer:  Prostate | Truly experimental | **Recall period**:  Not specified  **Asking for**:  Frequency | **Scoring algorithm**:  Frequency of mild × 3 + frequency of moderate × 5 + frequency of strenuous × 9  **Measurement unit**:  LSI (arbitrary units) | Assessment of baseline equivalence of the treatment and comparison groups  Outcome of a PA intervention | Relative ranking | Experimental conditions |
| L Bourke, G Thompson, DJ Gibson, A Daley, H Crank, I Adam, A Shorthouse and J Saxton [17] | *N* = 18- UK  Median age: 69  33% ♀  Type of cancer:  Colon | Truly experimental | **Recall period**:  Not specified  **Asking for**:  Frequency | **Scoring algorithm**:  Frequency of mild × 3 + frequency of moderate × 5 + frequency of strenuous × 9  **Measurement unit**:  LSI (arbitrary units) | Outcome of a PA intervention | Relative ranking | Experimental conditions |
| L Bourke, S Gilbert, R Hooper, LA Steed, M Joshi, JWF Catto, JM Saxton and DJ Rosario [18] | *N* = 100- UK  Mean age: 71  0% ♀  Type of cancer:  Prostate | Truly experimental | **Recall period**:  Not specified  **Asking for**:  Frequency | **Scoring algorithm**:  Frequency of mild × 3 + frequency of moderate × 5 + frequency of strenuous × 9  **Measurement unit**:  LSI (arbitrary units) | Assessment of baseline equivalence of the treatment and comparison groups  Outcome of a PA intervention | Relative ranking | Experimental conditions |
| JM Broderick, E Guinan, MJ Kennedy, D Hollywood, KS Courneya, SN Culos-Reed, K Bennett, OD DM and J Hussey [19] | *N* = 43- Ireland  Mean age: 51  86% ♀  Type of cancer:  Breast, colon, lymphoma, oesophageal. | Truly experimental | **Recall period**:  Typical week  **Asking for**:  Frequency | **Scoring algorithm**:  Frequency of mild × 3 + frequency of moderate × 5 + frequency of strenuous × 9  **Measurement unit**:  LSI (arbitrary units) | Outcome of a PA intervention | Relative ranking | Experimental conditions |
| J Brunet and CM Sabiston [20] | *N* = 169- Canada  Mean age: 55  100% ♀  73.4% completed college/university  Type of cancer:  Breast | Correlational- transversal | **Recall period**:  Not specified  **Asking for**:  Frequency and duration | **Scoring algorithm**:  Frequency × duration of mild × 3 + frequency × duration of moderate × 5 + frequency × duration of strenuous × 9  **Measurement unit**:  METs × minutes/week | Identify correlates of PA | Relative ranking | Self-presentational efficacy expectancy  Self-presentation outcome expectation and value |
| J Brunet, S Amireault, M Chaiton and CM Sabiston [21] | *N* = 199- Canada  Mean age: 55  100% ♀  50.7% completed college/university  Type of cancer:  Breast | Correlational- longitudinal | **Recall period**:  Typical week  **Asking for**:  Frequency and duration | **Scoring algorithm**:  Frequency × duration of moderate + frequency × duration of strenuous  **Measurement unit**:  Minutes/week | Identify correlates of PA  Assessment of PA change/patterns over time | Relative ranking | Cancer worry  Depressive symptoms  Fatigue  Cancer-related/medical  Physical symptoms  Socio-demographic variables |
| J Brunet, C Love, R Ramphal and CM Sabiston [22] | *N* = 64- Canada  Mean age: 29  73% ♀  57.8% completed college/university  Type of cancer:  Carcinoma, leukemia, lymphoma | Correlational- transversal | **Recall period**:  Last week  **Asking for**:  Frequency and duration | **Scoring algorithm**:  Frequency × duration of mild + frequency × duration of moderate + frequency × duration of strenuous  **Measurement unit**:  Minutes/week | Identify correlates of PA | Relative ranking | Social involvement  Social support  Stress |
| J Brunet, CM Sabiston and P Gaudreau [23] | *N* = 128- Canada  Mean age: 55  100% ♀  57.8% completed college/university  Type of cancer:  Breast | Correlational- longitudinal | **Recall period**:  Typical week  **Asking for**:  Frequency and duration | **Scoring algorithm**:  Frequency × duration of moderate × 5 + frequency × duration of strenuous × 9  **Measurement unit**:  METs/week | Identify correlates of PA  Assessment of PA change/patterns over time | Relative ranking | Cancer-related/medical  Self-presentation processes |
| AJ Burton, RM Martin, JL Donovan, JA Lane, M Davis, FC Hamdy, DE Neal and K Tilling [24] | *N* = 820- UK  Mean age: 62.5  0% ♀  Type of cancer:  Prostate | Correlational- longitudinal | **Recall period**:  Not specified  **Asking for**:  Frequency | **Scoring algorithm**:  Frequency of mild × 3 + frequency of moderate × 5 + frequency of strenuous × 9  **Measurement unit**:  LSI (arbitrary units) | Examine the association between PA and health-related outcomes | Relative ranking | PSA |
| S Casla, P Hojman, R Cubedo, I Calvo, J Sampedro and R Barakat [25] | *N* = 50- Spain  Mean age: 49  100% ♀  Type of cancer:  Breast | Pre-experimental | **Recall period**:  Not specified  **Asking for**:  Frequency | **Scoring algorithm**:  Frequency of mild × 3 + frequency of moderate × 5 + frequency of strenuous × 9  **Measurement unit**:  LSI (arbitrary units) | Examine the association between PA and health-related outcomes  Outcome of a PA intervention | Relative ranking | Depressive symptoms  Quality of life |
| MM Clark, KS Vickers, JC Hathaway, M Smith, SA Looker, LR Petersen, BM Pinto, TA Rummans and CL Loprinzi [26] | *N* = 128- USA  Mean age: 60  58% ♀  Type of cancer:  Breast, digestive, lung, gynecologic | Correlational- transversal | **Recall period**:  Typical week  **Asking for**:  Frequency | **Scoring algorithm**:  Frequency of mild; frequency of moderate; frequency of strenuous;  **Measurement unit**:  Number of ≥ 15 minutes LTPA cessions (frequency)/week | Examine the association between PA and health-related outcomes  Identify correlates PA | Relative ranking | Affect/mood  Socio-demographic variables  Social cognitive theory constructs  Well-being |
| ME Cooley, KT Finn, Q Wang, K Roper, S Morones, L Shi, D Litrownik, JP Marcoux, K Zaner and LL Hayman [27] | *N* = 37- USA  Median age: 59  46% ♀  51% completed college/graduate education  Type of cancer:  Lung | Correlational- transversal | **Recall period**:  Not specified  **Asking for**:  Frequency [and likely for duration; although, not specified] | **Scoring algorithm**:  Meeting PA recommendations:  Active; ≥ 150 minutes of MVPA or ≥ 60 minutes of vigorous PA;  *Insufficiently active*: < 150 minutes of MVPA and < 60 minutes of vigorous PA  **Measurement unit**:  Proportion (%) of *active* and *insufficiently active* individuals | Assess the prevalence of PA  Identify correlates of PA | Classification | % of family members meeting PA guidelines  Motivational readiness (Transtheoretical model construct) |
| P Cormie, RU Newton, N Spry, D Joseph, DR Taaffe and DA Galvao [28] | *N =* 20- Australia  Median age*:* 73  0% ♀  20% completed college/graduate education  Type of cancer:  Prostate | Truly experimental | **Recall period**:  Not specified  **Asking for**:  Frequency | **Scoring algorithm**:  Frequency of mild × 3 + frequency of moderate × 5 + frequency of strenuous × 9  **Measurement unit**:  LSI (arbitrary units) | Outcome of a PA intervention | Relative ranking | Experimental conditions |
| P Cormie, N Spry, K Jasas, M Johansson, IF Yusoff, RU Newton and DA Galvão [29] | *N =* 1- Australia  Age*:* 49  0% ♀  Type of cancer:  Pancreas | Case-study | **Recall period**:  Not specified  **Asking for**:  Frequency | **Scoring algorithm**:  Frequency of mild × 3 + frequency of moderate × 5 + frequency of strenuous × 9  **Measurement unit**:  LSI (arbitrary units) | Outcome of a PA intervention | Relative ranking | Experimental conditions/time |
| EJ Coups, BJ Park, MB Feinstein, RM Steingart, BL Egleston, DJ Wilson and JS Ostroff [30] | *N* = 175- USA  Median age: 70  63.4% ♀  ≥ 50% completed college/graduate education  Type of cancer:  Lung | Correlational- retrospective | **Recall period**:  Last week- at 3 time points:  a) During the 6 months before diagnostic;  b) During the 6 months following treatment  c) Current time  **Asking for**:  Frequency and duration | **Scoring algorithm**:  Frequency × duration of mild + frequency × duration of moderate + frequency × duration of strenuous  **Measurement unit**: Minutes/week  **Scoring algorithm**:  Meeting PA recommendations:  A*ctive*: ≥ 150 minutes of MVPA or ≥ 60 minutes of vigorous PA;  *Insufficiently active*: < 150 minutes of MVPA and < 60 minutes of vigorous PA; *Sedentary*: no mild, moderate and strenuous PA  **Measurement unit**:  Proportion (%) of *active,* *insufficiently active*, and *sedentary* individuals | Assessment of PA patterns during cancer experience  Examine the association between PA and health-related outcomes | Relative ranking  Classification | Quality of life |
| EJ Coups, BJ Park, MB Feinstein, RM Steingart, BL Egleston, DJ Wilson and JS Ostroff [31] | *N* = 175- USA  Mean age: 68.7  63.4% ♀  ≥ 50% completed college/graduate education  Type of cancer:  Lung | Correlational- transversal | **Recall period**:  Last week  **Asking for**:  Frequency and duration | **Scoring algorithm**:  Frequency × duration of moderate + frequency × duration of strenuous  **Measurement unit**: Minutes/week | Identify correlates of PA | Relative ranking | Social cognitive theory constructs |
| KS Courneya, CM Blanchard and DM Laing [32] | *N* = 24- Canada  Mean age: 51.1  100% ♀  44% completed graduate education  Type of cancer:  Breast | Correlational- longitudinal | **Recall period**:  Last month  **Asking for**:  Frequency | **Scoring algorithm**:  Frequency of mild × 3 + frequency of moderate × 5 + frequency of strenuous × 9  **Measurement unit**:  LSI (arbitrary units) | Identify correlates of PA | Relative ranking | Theory of planned behavior constructs |
| KS Courneya and CM Friedenreich [33] | *N* = 111- Canada  Mean age: 61  37% ♀  34% completed college or undergraduate education  Type of cancer:  Colorectal | Correlational- retrospective | **Recall period**:  Typical week-  Recall LTPA during treatment for cancer  **Asking for**:  Frequency | **Scoring algorithm**:  Frequency of mild × 3 + frequency of moderate × 5 + frequency of strenuous × 9  **Measurement unit**:  LSI (arbitrary units) | Identify correlates of PA | Relative ranking | Cancer-related/medical variables  Socio-demographic variables  Theory of planned behaviour constructs |
| KS Courneya and CM Friedenreich [34] | *N* = 167- Canada  Mean age: 53.1  100% ♀  34.2% completed college or undergraduate education  Type of cancer:  Breast | Correlational- retrospective | **Recall period**:  Typical week-  a) During the month prior cancer diagnosis    b) During treatment  c) After treatment    **Asking for**:  Frequency | **Scoring algorithm**:  Frequency of mild × 3 + frequency of moderate × 5 + frequency of strenuous × 9  **Measurement unit**:  LSI (arbitrary units)  **Scoring algorithm**:  Meeting PA recommendations:  *Active*: ≥ 1 bout of moderate and strenuous LTPA; *Insufficiently active*: 0 bout of moderate and strenuous LTPA  **Measurement unit**:  Proportion (%) of *active*, *inactive*, maintainers*, temporary relapsers, permanent relapsers and nonexercisers* | Examine the association between PA patterns and health-related outcomes | Relative ranking  Classification | Quality of life  Satisfaction  with life |
| KS Courneya and CM Friedenreich [35] | *N* = 130- Canada  Mean age: 40.9  46% ♀  30.0% completed college or undergraduate education  Type of cancer:  Colorectal | Correlational- retrospective | **Recall period**:  Typical week-  a) During the month prior cancer diagnosis;  b) During treatment;  c) After treatment  **Asking for**:  Frequency | **Scoring algorithm**:  Frequency of mild × 3 + frequency of moderate × 5 + frequency of strenuous × 9  Meeting PA recommendations:  *Active*: ≥ 16 LSI; *Insufficiently active*: < 16 LSI  **Measurement unit**:  LSI (arbitrary units)  Proportion (%) of *maintainers, temporary relapsers, permanent relapsers and nonexercisers* | Assessment of PA patterns during cancer experience  Examine the association between PA patterns and health-related outcomes | Relative ranking  Classification | Quality of life  Time |
| KS Courneya and CM Friedenreich [36] | *N* = 164- Canada  Mean age: 53  100% ♀  34% completed graduate education  Type of cancer:  Breast | Correlational- retrospective | **Recall period**:  Typical week-during cancer treatment  **Asking for**:  Frequency | **Scoring algorithm**:  Frequency of mild × 3 + frequency of moderate × 5 + frequency of strenuous × 9  **Measurement unit**:  LSI (arbitrary units) | Identify correlates of PA | Relative ranking | Theory of planned behaviour constructs |
| KS Courneya, CM Friedenreich, K Arthur and TM Bobick [37] | *N* = 53- Canada  Mean age: 51.6  40% ♀  Type of cancer:  Colorectal | Correlational- retrospective | **Recall period**:  Typical week in:  a) The month prior to cancer diagnosis  b) At post-treatment (during the past month)  **Asking for**:  Frequency | **Scoring algorithm**:  Frequency of mild; frequency of moderate; frequency of strenuous.  **Measurement unit**:  Number of ≥ 15 minutes LTPA cessions (frequency)/week | Examine the association between PA and health-related outcomes | Relative ranking | Quality of life |
| KS Courneya, CM Friedenreich, K Arthur and TM Bobick [38] | *N* = 66- Canada  Mean age: 40.9  84.4% ♀  31.8% completed college or undergraduate education  Type of cancer:  Colorectal | Correlational- longitudinal | **Recall period**:  Typical week  **Asking for**:  Frequency | **Scoring algorithm**:  Frequency of mild × 3 + frequency of moderate × 5 + frequency of strenuous × 9  **Measurement unit**:  LSI (arbitrary units) | Identify correlates of PA | Relative ranking | Theory of planned behavior constructs |
| KS Courneya, CM Friedenreich, HA Quinney, ALA Fields, LW Jones and AS Fairey [39] | *N* = 93- Canada  Mean age: 60.3  41.9% ♀  39% completed undergraduate education  Type of cancer:  Colorectal | Truly experimental | **Recall period**:  a) Last month  b) Last week  **Asking for**:  Frequency and duration | **Scoring algorithm**:  Frequency × duration moderate + frequency × duration of strenuous  Meeting PA recommendations:  *Active*: ≥ 150 minutes of MVPA or ≥ 60 minutes of strenuous PA;  *Insufficiently active*: < 150 minutes of MVPA and < 60 minutes of strenuous PA  **Measurement unit**: Minutes/week  Proportion (%) of *active* and *insufficiently active* individuals | Assessment of baseline equivalence of the treatment and comparison groups  Assess contamination rate between conditions | Relative ranking  Classification | Experimental conditions |
| KS Courneya, CM Friedenreich, HA Quinney, ALA Fields, LW Jones and AS Fairey [40] | *N* = 93- Canada  Mean age: 60.3  41.9% ♀  36% completed undergraduate education  Type of cancer:  Colorectal | Truly experimental | **Recall period**:  a) Last month  b) Last week  **Asking for**:  Frequency and duration | **Scoring algorithm**:  Frequency × duration moderate + frequency × duration of strenuous  Meeting PA recommendations:  *Active*: ≥ 150 minutes of MVPA or ≥ 60 minutes of strenuous PA;  *Insufficiently active*: < 150 minutes of MVPA and < 60 minutes of strenuous PA  **Measurement unit**: Minutes/week  Proportion (%) of *active* and *insufficiently active* individuals | Identify correlates of adherence and contamination in RCT  Assess contamination rate between conditions  Assess fidelity to PA supervised intervention | Relative ranking  Classification | N/A |
| KS Courneya, CM Friedenreich, HA Quinney, ALA Fields, LW Jones, JKH Vallance and AS Fairey [41] | *N* = 69- Canada  Mean age: 59.9  43.5% ♀  34.8% completed undergraduate education  Type of cancer:  Colorectal | Correlational- longitudinal | **Recall period**:  Last week  **Asking for**:  Frequency and duration | **Scoring algorithm**:  Meeting PA recommendations:  *Active*: ≥ 30 minutes of MVPA at least 3 times/week;  *Insufficiently active*: < 30 minutes of MVPA at least 3 times/week  **Measurement unit**:  Proportion (%) of *active* and *insufficiently active* individuals | Identify correlates of PA | Classification | Exercise barriers |
| KS Courneya, CM Friedenreich, RD Reid, K Gelmon, JR Mackey, AB Ladha, C Proulx, JK Vallance and RJ Segal [42] | *N* = 242- Canada  Mean age: 49  100% ♀  23% completed graduate education  Type of cancer:  Breast | Correlational-  Longitudinal | **Recall period**:  Last 6 months  **Asking for**:  Frequency and duration | **Scoring algorithm**:  Meeting PA recommendations:  *Active*: ≥ 150 minutes of MVPA or ≥ 60 minutes of strenuous PA;  *Insufficiently active*: < 150 minutes of MVPA and < 60 minutes of strenuous PA  **Measurement unit**:  Proportion (%) of *active* and *insufficiently active* individuals | Identify correlates of PA | Classification | Clinical/medical variables  Socio-demographic variables  Theory of planned behaviour constructs |
| KS Courneya, CM Friedenreich, RA Sela, HA Quinney and RE Rhodes [43] | *N* = 96- Canada  Mean age: 51.6  84.4% ♀  57.8% completed undergraduate education  Type of cancer:  Breast and others | Truly experimental | **Recall period**:  Typical week  **Asking for**:  Frequency and duration | **Scoring algorithm**:  Frequency × duration moderate + frequency × duration of strenuous  Minimum prescription for PA: *Active*: ≥ 3 times/week for at least 20 minutes; *Insufficiently active*: < 3 times/week for at least 20 minutes  Optimum prescription for PA: *Active*: ≥ 5 times/week for at least 150 minutes; *Insufficiently active*: < 5 times/week for at least 150 minutes  **Measurement unit**:  Number of ≥ 15 minutes LTPA cessions (frequency)/week  Proportion (%) of *active* and *insufficiently active* individuals | Assessment of baseline equivalence of the treatment and comparison groups  Identify correlates of adherence and contamination in RCT | Relative ranking  Classification | Cancer-related/medical variables  Experimental conditions  Socio-demographic variables  Theory of planned behavior constructs |
| KS Courneya, CM Friedenreich, RA Sela, HA Quinney, RE Rhodes and M Handman [44] | *N* = 108- Canada  Mean age: 51.6  84.4% ♀  58.7% completed graduate education  Type of cancer:  Breast, colon, ovarian, stomach, melanoma | Truly experimental | **Recall period**:  Last month and Last week  **Asking for**:  Frequency and duration | **Scoring algorithm**:  Frequency × duration of mild + frequency × duration of moderate + frequency × duration of strenuous  Frequency × duration of moderate + frequency × duration of strenuous  Frequency × duration of mild; moderate; and strenuous  Meeting PA recommendations:  *Active*: ≥ 60 minutes of MVPA;  *Insufficiently active*: < 60 minutes of MVPA  **Measurement unit**: Minutes/week  Proportion (%) of *active* and *insufficiently active* individuals | Assessment of baseline equivalence of the treatment and comparison groups  Assess fidelity to PA supervised intervention | Relative ranking  Classification | N/A |
| KS Courneya, CM Friedenreich, RA Sela, HA Quinney, RE Rhodes and LW Jones [45] | *N* = 30- Canada  Mean age: 52.8  80% ♀  55% completed undergraduate education  Type of cancer:  Breast, and other types of cancers | Correlational- longitudinal | **Recall period**:  Last 5 weeks  **Asking for**:  Frequency and duration | **Scoring algorithm**:  Frequency × duration moderate + frequency × duration of strenuous  Frequency of moderate + frequency of strenuous  **Measurement unit**:  Minutes/week  Number of ≥ 15 minutes LTPA cessions (frequency)/week | Identify correlates of PA | Relative ranking | Attribution theory constructs |
| KS Courneya, LW Jones, CJ Peddle, CM Sellar, T Reiman, AA Joy, N Chua, L Tkachuk and JR Mackey [46] | *N* = 55- Canada  Mean age: 56  84.4% ♀  52.7% completed graduate education  Type of cancer:  Breast | Truly experimental | **Recall period**:  Not specified  **Asking for**:  Frequency [and likely for duration; although, not specified] | **Scoring algorithm**:  Not specified  **Measurement unit**:  Proportion (%) of *active* and *insufficiently active* individuals | Assessment of baseline equivalence of the treatment and comparison groups | Classification | Experimental conditions |
| KS Courneya, KH Karvinen, KL Campbell, RG Pearcey, G Dundas, V Capstick and KS Tonkin [47] | *N* = 386- Canada  Mean age: 64.5  100% ♀  25.4% completed graduate education  Type of cancer:  Endometrial | Correlational- retrospective | **Recall period**:  Typical week-  a) Before diagnosis;  b) During treatment;  c) In the last month  **Asking for**:  Frequency and duration | **Scoring algorithm**:  Frequency × duration of mild + frequency × duration of moderate + frequency × duration of strenuous  Frequency × duration of moderate + frequency × duration of strenuous  Frequency × duration of mild; moderate; and strenuous  Meeting PA recommendations:  *Active*: ≥ 150 minutes of MVPA or ≥ 60 minutes of strenuous PA;  *Insufficiently active*: < 150 minutes of MVPA and < 60 minutes of strenuous PA  **Measurement unit**: Minutes/week  Proportion (%) of *active* and *insufficiently active* individuals | Assess the prevalence of PA  Examine the association between PA and health-related outcomes | Relative ranking  Classification | Quality of life  Body mass index |
| KS Courneya, MR Keats and AR Turner [48] | *N* = 25- Canada  Mean age: 47.2  52% ♀  36% completed graduate education  Type of cancer:  Breast, Hodgkin’s and non-Hodgkin’s lymphoma, multiple myeloma, others | Correlational- retrospective | **Recall period**:  Typical week-  a) Before diagnosis  b) After diagnosis  **Asking for**:  Frequency | **Scoring algorithm**:  Frequency of mild, moderate and strenuous  **Measurement unit**: Number of LTPA bouts (frequency)/week | Assessment of PA patterns during cancer experience | Relative ranking | Time |
| KS Courneya, MR Keats and AR Turner [49] | *N* = 37- Canada  Mean age: 40.9  84.4% ♀  31.8% completed college or undergraduate education  Type of cancer:  Breast, Hodgkin and non-Hodgkin lymphoma and other cancers | Correlational- longitudinal | **Recall period**:  Typical week-  During the month prior cancer diagnosis and during treatment  **Asking for**:  Frequency | **Scoring algorithm**:  Frequency of mild × 3 + frequency of moderate × 5 + frequency of strenuous × 9  **Measurement unit**: LSI (arbitrary units) | Identify correlates of PA | Relative ranking | Theory of planned behaviour constructs |
| KS Courneya, JR Mackey, GJ Bell, LW Jones, CJ Field and AS Fairey [50] | *N* = 122- Canada  Mean age: 53.2  41% ♀  51.6% completed undergraduate education  Type of cancer:  Hodgkin’s and non-Hodgkin’s lymphoma | Truly experimental | **Recall period**:  Not specified  **Asking for**:  Frequency and duration | **Scoring algorithm**:  Meeting PA recommendations:  *Active*: ≥ 150 minutes of MVPA;  *Insufficiently active*: < 150 minutes of MVPA  **Measurement unit**:  Proportion (%) of *active* and *insufficiently active* individuals | Assessment of baseline equivalence of the treatment and comparison groups  Served as a control variable in the analyses | Classification | Experimental conditions |
| KS Courneya, DC McKenzie, RD Reid, JR Mackey, K Gelmon, CM Friedenreich, AB Ladha, C Proulx, K Lane, JK Vallance, et al. [51] | *N* = 242- Canada  Mean age: 49  100% ♀  Type of cancer:  Breast | Truly experimental | **Recall period**:  Not specified  **Asking for**:  Frequency and duration | **Scoring algorithm**:  Meeting PA recommendations:  *Active*: ≥ 150 minutes of MVPA or ≥ 60 minutes of strenuous PA;  *Insufficiently active*: < 150 minutes of MVPA and < 60 minutes of strenuous PA  **Measurement unit**:  Proportion (%) of *active* and *insufficiently active* individuals | Assessment of baseline equivalence of the treatment and comparison groups  Identify correlates of PA | Classification | Experimental conditions  PA barriers |
| KS Courneya, RD Reid, CM Friedenreich, K Gelmon, C Proulx, JK Vallance, DC McKenzie and RJ Segal [52] | *N* = 242- Canada  Mean age: 49  100% ♀  Type of cancer:  Breast | Truly experimental | **Recall period**:  Not specified  **Asking for**:  Frequency and duration | **Scoring algorithm**:  Meeting PA recommendations:  *Active*: ≥ 150 minutes of MVPA or ≥ 60 minutes of strenuous PA;  *Insufficiently active*: < 150 minutes of MVPA and < 60 minutes of strenuous PA  **Measurement unit**:  Proportion (%) of *active* and *Insufficiently active* individuals | Determine exercise type, counselling modalities preferences | Classification | Satisfaction with exercise protocol |
| KS Courneya, RJ Segal, K Gelmon, RD Reid, JR Mackey, CM Friedenreich, C Proulx, K Lane, AB Ladha, JK Vallance, et al. [53] | *N* = 242- Canada  Mean age: 49  100% ♀  Type of cancer:  Breast | Truly experimental-  6-month following the end of a RCT | **Recall period**:  Last 6 months  **Asking for**:  Frequency and duration | **Scoring algorithm**:  Meeting PA recommendations:  *Active*: ≥ 150 minutes of MVPA or ≥ 60 minutes of strenuous PA;  *Insufficiently active*: < 150 minutes of MVPA and < 60 minutes of strenuous PA  **Measurement unit**:  Proportion (%) of *active* and *insufficiently active* individuals | Examine the association between PA patterns and health-related outcomes | Classification | Depression  Fatigue  Quality of life  Self-esteem |
| KS Courneya, RJ Segal, K Gelmon, RD Reid, JR Mackey, CM Friedenreich, C Proulx, K Lane, AB Ladha, JK Vallance, et al. [54] | *N* = 242- Canada  Mean age: 49  100% ♀  Type of cancer:  Breast | Truly experimental | **Recall period**:  Not specified  **Asking for**:  Frequency and duration | **Scoring algorithm**:  Meeting PA recommendation:  *Active*: ≥ 150 minutes of MVPA or ≥ 60 minutes of strenuous PA;  *Insufficiently active*: < 150 minutes of MVPA and < 60 minutes of strenuous PA  **Measurement unit**:  Proportion (%) of *Active* and *Insufficiently active* Individuals | Identify correlates of PA | Classification | Theory of planned behaviour constructs |
| KS Courneya, RJ Segal, JR Mackey, K Gelmon, RD Reid, CM Friedenreich, AB Ladha, C Proulx, JKH Vallance, K Lane, et al. [55] | *N* = 242- Canada  Mean age: 49  100% ♀  Type of cancer:  Breast | Truly experimental | **Recall period**:  Not specified  **Asking for**:  Frequency and duration | **Scoring algorithm**:  Meeting PA recommendations:  *Active*: ≥ 150 minutes of MVPA or ≥ 60 minutes of strenuous PA;  *Insufficiently active*: < 150 minutes of MVPA and < 60 minutes of strenuous PA  **Measurement unit**:  Proportion (%) of *Active* and *Insufficiently active* individuals | Assessment of baseline equivalence of the treatment and comparison groups  Assess fidelity to PA supervised intervention | Classification | Experimental conditions |
| KS Courneya, RJ Segal, RD Reid, LW Jones, SC Malone, PM Venner, MB Parliament, CG Scott, HA Quinney and GA Wells [56] | *N* = 155- Canada  Mean age: 68  0% ♀  Type of cancer:  Prostate | Truly experimental | **Recall period**:  Typical week the month before cancer diagnosis  **Asking for**:  Frequency | **Scoring algorithm**:  Frequency of mild; frequency of moderate; frequency of strenuous.  Meeting PA recommendations:  *Active*: ≥ 3 times/week  *Insufficiently active*: < 3times/week  **Measurement unit**:  Number of ≥ 15 minutes LTPA cessions (frequency)/week  Proportion (%) of *active* and *insufficiently active* individuals | Assessment of baseline equivalence of the treatment and comparison groups | Relative ranking  Classification | Experimental conditions |
| KS Courneya, CM Sellar, C Stevinson, ML McNeely, CJ Peddle, CM Friedenreich, K Tankel, S Basi, N Chua, A Mazurek, et al. [57] | *N* = 53- Canada  Mean age: 59  100% ♀  44% completed undergraduate education  Type of cancer:  Breast | Truly experimental | **Recall period**:  Not specified  **Asking for**:  Frequency and duration | **Scoring algorithm**:  Frequency × duration moderate + frequency × duration of strenuous  Frequency × duration moderate  Frequency × duration strenuous  Meeting PA recommendations:  *Active*: ≥ 90 minutes of MVPA;  *Insufficiently active*: < 90 minutes of MVPA  **Measurement unit**: Minutes/week  Proportion (%) of *active* and *insufficiently active* individuals | Assessment of baseline equivalence of the treatment and comparison groups  Assess fidelity to PA supervised intervention | Relative ranking  Classification | Experimental conditions |
| KS Courneya, CM Sellar, L Trinh, CC Forbes, C Stevinson, ML McNeely, CJ Peddle-McIntyre, CM Friedenreich and T Reiman [58] | *N* = 122- Canada  Mean age: 53.2  41% ♀  51.6% completed undergraduate education  Type of cancer:  Hodgkin’s and non-Hodgkin’s lymphoma | Truly experimental | **Recall period**:  Not specified  **Asking for**:  Frequency and duration | **Scoring algorithm**:  Meeting PA recommendations*:*  *Active*: ≥ 150 minutes of MVPA;  *Insufficiently active*: < 150 minutes of MVPA  **Measurement unit**:  Proportion (%) of *active* and *insufficiently active* individuals | Assessment of baseline equivalence of the treatment and comparison groups  Served as a control variable in the analyses | Classification | Experimental conditions |
| KS Courneya, C Stevinson, ML McNeely, CM Sellar, CM Friedenreich, CJ Peddle-Mcintyre, N Chua and T Reiman [59] | *N* = 122- Canada  Mean age: 53  41% ♀  Type of cancer:  Hodgkin and non-Hodgkin lymphoma | Truly experimental | **Recall period**:  Last month  **Asking for**:  Frequency and duration | **Scoring algorithm**:  Frequency × duration moderate + frequency × duration of strenuous  Meeting PA recommendations:  *Active*: ≥ 150 minutes of MVPA or ≥ 75 minutes of strenuous PA;  *Insufficiently active*: < 150 minutes of MVPA and < 75 minutes of strenuous PA  **Measurement unit**: Minutes/week  Proportion (%) of *active* and *insufficiently active* individuals | Examine the association between PA and health-related outcomes  Identify correlates of PA (mediators of the intervention) | Relative ranking  Classification | Theory of planned behaviour constructs |
| KS Courneya, C Stevinson, ML McNeely, CM Sellar, CM Friedenreich, CJ Peddle-McIntyre, N Chua and T Reiman [60] | *N* = 122- Canada  Mean age: 53.2  41% ♀  51.6% completed undergraduate education  Type of cancer:  Hodgkin’s and non-Hodgkin’s lymphoma | Correlational-  Longitudinal | **Recall period**:  Last month  **Asking for**:  Frequency and duration | **Scoring algorithm**:  Meeting PA recommendations:  *Active*: ≥ 150 minutes of MVPA or ≥ 75 minutes of strenuous PA;  *Insufficiently active*: < 150 minutes of MVPA and < 75 minutes of strenuous PA  **Measurement unit**:  Proportion (%) of *active* and *insufficiently active* individuals | Identify correlates of PA | Classification | Cancer-related/medical variables  Socio-demographic variables  Theory of planned behaviour constructs |
| KS Courneya, C Stevinson, ML McNeely, CM Sellar, CJ Peddle, CM Friedenreich, A Mazurek, N Chua, K Tankel, S Basi, et al. [61] | *N* = 122- Canada  Mean age: 53  41% ♀  Type of cancer:  Hodgkin’s and non-Hodgkin’s lymphoma | Truly experimental | **Recall period**:  Last month  **Asking for**:  Frequency and duration | **Scoring algorithm**:  Meeting PA recommendations:  *Active*: ≥ 150 minutes of MVPA or ≥ 75 minutes of strenuous PA;  *Insufficiently active*: < 150 minutes of MVPA and < 75 minutes of strenuous PA; *sedentary*: 0 minutes of mild/moderate/ strenuous PA/week  **Measurement unit**:  Proportion (%) of *active, insufficiently active* and *sedentary* individuals | Identify correlates of PA | Classification | Exercise adherence |
| KS Courneya, JKH Vallance, LW Jones and T Reiman [62] | *N* = 399- Canada  Mean age: 61  48% ♀  29% completed undergraduate education  Type of cancer:  Hodgkin’s and non-Hodgkin’s lymphoma | Correlational-  Transversal | **Recall period**:  Typical week  **Asking for**:  Frequency and duration | **Scoring algorithm**:  Frequency × duration of moderate + frequency × duration of strenuous  **Measurement unit**: Minutes/week | Identify correlates of PA | Relative ranking | Cancer-related/medical variables  Socio-demographic variables  Theory of planned behaviour constructs |
| M Craike, K Hose and PM Livingston [63] | *N* = 229- Australia  Median age: 60  46.9% ♀  Type of cancer:  Multiple myeloma | Correlational- retrospective | **Recall period**:  a) Typical week- prior to diagnosis  b) Last month-at the time of the survey  **Asking for**:  Frequency and duration | **Scoring algorithm**:  Meeting PA recommendations*:  *Active*: ≥ 150 minutes of at least moderate intensity/week;  *Insufficiently active*: < 150 minutes of MVPA of at least moderate intensity/week  *Minutes of strenuous LTPA were multiplied by 2  **Measurement unit**:  Proportion (%) of *active* and *insufficiently active* individuals | Assessment of PA patterns during cancer experience  Identify correlates of PA | Classification | Cancer-related/medical variables  PA barriers  Socio-demographic variables |
| MJ Craike, K Hose, KS Courneya, SJ Harrison and PM Livingston [64] | *N* = 24- Australia  Mean age: 62  54% ♀  42% completed undergraduate education  Type of cancer:  Multiple myeloma | Correlational- transversal  (qualitative study) | **Recall period**:  a) Typical week- prior to diagnosis  b) Last month-at the time of the survey  **Asking for**:  Frequency and duration | **Scoring algorithm**:  Frequency × duration of moderate + frequency × duration of strenuous  Meeting PA recommendations*:  *Active*: ≥ 150 minutes of MVPA/week;  *Insufficiently active*: < 150 minutes of MVPA/week;  *Minutes of strenuous LTPA were multiplied by 2  **Measurement unit**: Minutes/week  Proportion (%) of *active* and *insufficiently active* individuals | Assess the prevalence of PA  Assessment of PA patterns during cancer experience | Relative ranking  Classification | N/A |
| AD Crosswell, KG Lockwood, PA Ganz and JE Bower [65] | *N* = 84- USA  Mean age: 47  100% ♀  42% completed college/university  Type of cancer:  Breast | Correlational- transversal | **Recall period**:  Typical week  **Asking for**:  Frequency and duration | **Scoring algorithm**:  Frequency × duration of mild × 3 + frequency × duration of moderate × 5 + frequency × duration of strenuous × 9  **Measurement unit**:  MET hours/week | Served as a control variable in the analyses | Relative ranking | N/A |
| T Crowgey, KB Peters, WE Hornsby, A Lane, F McSherry, JE Herndon, 2nd, MJ West, CL Williams and LW Jones [66] | *N* = 37- USA  Mean age: 52  100% ♀  Type of cancer:  Breast | Case-control | **Recall period**:  Last month  **Asking for**:  Frequency and duration | **Scoring algorithm**:  Frequency × duration of mild + frequency × duration of moderate + frequency × duration of strenuous  Frequency × duration of moderate + frequency × duration of strenuous  Meeting PA recommendations:  *Active*: ≥ 150 minutes of MVPA/week;  *Insufficiently active*: < 150 minutes of MVPA/week  **Measurement unit**:  Minutes/week  Proportion (%) of *active* and *insufficiently active* individuals | Assessment of baseline PA to test difference across health conditions  Examine the association between PA and health-related outcomes | Relative ranking  Classification | Cognitive function |
| SN Culos-Reed, LE Carlson, LM Daroux and S Hately-Aldous [67] | *N* = 38- Canada  Median age: 50  95% ♀  Type of cancer:  Breast | Truly experimental | **Recall period**:  Last month  **Asking for**:  Frequency | **Scoring algorithm**:  Not specified  **Measurement unit**:  Not specified | Assessment of baseline equivalence of the treatment and comparison groups | Not specified | N/A |
| SN Culos-Reed, JL Robinson, H Lau, K O'Connor and MR Keats [68] | *N* = 31- Canada  Mean age: 65  0% ♀  26% completed undergraduate education  Type of cancer:  Prostate | Pre-experimental | **Recall period**:  Typical week- over the last 12 weeks  **Asking for**:  Frequency | **Scoring algorithm**:  Frequency of mild × 3 + frequency of moderate × 5 + frequency of strenuous × 9  **Measurement unit**:  LSI (arbitrary units) | Outcome of a PA intervention | Relative ranking | Experimental conditions |
| SN Culos-Reed, JW Robinson, H Lau, L Stephenson, M Keats, S Norris, G Kline and P Faris [69] | *N* = 31- Canada  Mean age: 68  0% ♀  44% completed undergraduate education  Type of cancer:  Prostate | Truly experimental | **Recall period**:  Typical week- over the last 12 weeks  **Asking for**:  Frequency | **Scoring algorithm**:  Frequency of mild × 3 + frequency of moderate × 5 + frequency of strenuous × 9  **Measurement unit**:  LSI (arbitrary units) | Assessment of baseline equivalence of the treatment and comparison groups  Outcome of a behavioural intervention | Relative ranking | Experimental conditions |
| SN Culos-Reed, C Shields and LR Brawley [70] | *N* = 56- Canada  Mean age: 53  100% ♀  48% completed undergraduate education  Type of cancer:  Breast | Correlational- longitudinal | **Recall period**:  Typical week- over the last month  **Asking for**:  Frequency and duration | **Scoring algorithm**:  Frequency × duration of mild + frequency × duration of moderate + frequency × duration of strenuous PA  **Measurement unit**: Minutes/week | Examine the association between PA patterns and health-related outcomes  Identify correlates of PA | Relative ranking | Theory of planned behaviour constructs |
| W Demark-Wahnefried, C Werner, EC Clipp, AB Guill, M Bonner, LW Jones and PM Rosoff [71] | *N* = 209- USA  Mean age: 20  50% ♀  Type of cancer:  Leukemia, lymphoma, CNS | Correlational- transversal | **Recall period**:  Not specified  **Asking for**:  Frequency | **Scoring algorithm**:  Frequency of moderate + strenuous  **Measurement unit**: Number of ≥ 15 minutes LTPA cessions (frequency)/week  **Scoring algorithm**:  Not specified  **Measurement unit**: Proportion (%) of *active* and *insufficiently active* individuals | Assess prevalence of PA  Examine the association between PA and health-related outcomes  Identify correlates of PA  Investigate interest and preferences for exercise | Relative ranking  Classification | Quality of life  Clinical/medical variables  Socio-demographic variables  Interest in exercise counselling |
| W Demark-Wahnefried, LW Jones, DC Snyder, RJ Sloane, GG Kimmick, DC Hughes, HJ Badr, PE Miller, LE Burke and IM Lipkus [72] | *N* = 68- USA  (mother)  Mean age: 61  100% ♀  33% completed college/university  Type of cancer:  Breast | Truly experimental | **Recall period**:  Not specified  **Asking for**:  Frequency and duration | **Scoring algorithm**:  Frequency × duration of moderate + frequency × duration of strenuous  METs: Not specified  **Measurement unit**:  Minutes/week  MET hours/week | Outcome of an intervention | Relative ranking | Experimental conditions |
| KA Donovan, BJ Small, MA Andrykowski, P Munster and PB Jacobsen [73] | *N* = 261- USA  Mean age: 55  100% ♀  Mean years of education: 14  Type of cancer:  Breast | Correlational- longitudinal | **Recall period**:  Last week  **Asking for**:  Frequency | **Scoring algorithm**:  Frequency of mild × 3 + frequency of moderate × 5 + frequency of strenuous × 9  **Measurement unit**:  LSI (arbitrary units) | Examine the association between PA and health-related outcomes | Relative ranking | Fatigue |
| AS Fairey, KS Courneya, CJ Field, GJ Bell, LW Jones and JR Mackey [74] | *N* = 52- Canada  Mean age: 59  100% ♀  Type of cancer:  Breast | Truly experimental | **Recall period**:  Not specified  **Asking for**:  Frequency and duration | **Scoring algorithm**:  Frequency × duration of moderate  Frequency × duration of strenuous  Meeting PA recommendations:  *Active*: ≥ 90 minutes of MVPA/week; *Insufficiently active*: < 90 minutes of MVPA/week  **Measurement unit**: Minutes/week  Proportion (%) of *active* and *insufficiently active* individuals | Assessment of baseline equivalence of the treatment and comparison groups  Assess contamination rate between conditions | Relative ranking  Classification | Experimental conditions |
| AS Fairey, KS Courneya, CJ Field, GJ Bell, LW Jones and JR Mackey [75] | *N* = 52- Canada  Mean age: 59  100% ♀  Type of cancer:  Breast | Truly experimental | **Recall period**:  Not specified  **Asking for**:  Frequency and duration | **Scoring algorithm**:  Frequency × duration of moderate + frequency × duration of strenuous;  Frequency × duration of moderate; and strenuous  Meeting PA recommendations:  *Active*: ≥ 90 minutes of MVPA intensity/week; *Insufficiently active*: < 90 minutes of intensity/week  **Measurement unit**:  Minutes/week  Proportion (%) of *active* and *insufficiently active* individuals | Assessment of baseline equivalence of the treatment and comparison groups  Assess contamination rate between conditions | Relative ranking  Classification | Experimental conditions |
| LA Faul, HS Jim, S Minton, M Fishman, T Tanvetyanon and PB Jacobsen [76] | *N* = 192- USA  Mean age: 56  67% ♀  40% completed college education  Type of cancer:  Breast, lung, other cancers | Correlational- transversal | **Recall period**:  Last week  **Asking for**:  Frequency | **Scoring algorithm**:  Frequency of mild × 3 + frequency of moderate × 5 + frequency of strenuous × 9  *No exercise group*: No LSI (score = 0);  *Mild exercise group*: mild as the highest level of PA; *Moderate exercise group*: moderate as the highest level of PA;  *Strenuous exercise group*: Strenuous as the highest level of PA  **Measurement unit**:  LSI (arbitrary units)  Proportion (%) of individuals in the *no exercise, mild exercise, moderate exercise* and *strenuous* groups | Examine the association between PA patterns and health-related outcomes | Relative ranking  Classification | Anxiety  Depression  Quality of life |
| MB Feinstein, P Krebs, EJ Coups, BJ Park, RM Steingart, J Burkhalter, A Logue and JS Ostroff [77] | *N* = 342- USA  Mean age: 69  64% ♀  68% completed college education  Type of cancer:  Lung | Correlational- transversal | **Recall period**:  Not specified  **Asking for**:  Frequency | **Scoring algorithm**:  *Any moderate/strenuous PA group*: moderate/strenuous frequency ≥ 0;  *No moderate/strenuous PA group*: moderate/strenuous frequency = 0.  Proportion (%) of individuals in the *any moderate/strenuous PA* and n*o moderate/strenuous* groups | Examine the association between PA patterns and health-related outcomes | Classification | Dyspnea |
| CC Forbes, CM Blanchard, WK Mummery and KS Courneya [78] | *N* = 741- Canada  Median age: 65  45% ♀  51% completed college/university  Type of cancer:  Breast, colorectal, prostate | Correlational- transversal | **Recall period**:  Last month  **Asking for**:  Frequency and duration | **Scoring algorithm**:  MVPA: Frequency × duration of moderate + frequency × duration of strenuous × 2  Meeting PA recommendations:  *Active*: ≥ 150 minutes of MVPA/week or ≥ 75 minutes of strenuous PA/week;  *Insufficiently active*: < 150 minutes of MVPA/week and ≥ 75 minutes of strenuous PA/week  **Measurement unit**:  *Continuous scores*-  Minutes/week  *Categorical score*-  Proportion (%) of *active* and *insufficiently active* | Identify correlates of PA | Relative ranking  Classification | Cancer-related/medical variables  Theory of planned behaviour constructs  Socio-demographic variables |
| DA Galvão, N Spry, J Denham, DR Taaffe, P Cormie, D Joseph, DS Lamb, SK Chambers and RU Newton [79] | *N* = 100- Australia and New-Zealand  Mean age: 72  0% ♀  Type of cancer:  Prostate | Truly experimental | **Recall period**:  Not specified  **Asking for**:  Frequency | **Scoring algorithm**:  Frequency of mild × 3 + frequency of moderate × 5 + frequency of strenuous × 9  **Measurement unit**:  LSI (arbitrary units) | Assessment of baseline equivalence of the treatment and comparison groups | Relative ranking | Experimental conditions |
| SE Gilbert, GA Tew, L Bourke, EM Winter and DJ Rosario [80] | *N* = 20- UK  Mean age: 69  0% ♀  Type of cancer:  Prostate | Case-control | **Recall period**:  Last 6 months  **Asking for**:  Frequency | **Scoring algorithm**:  Frequency of mild × 3 + frequency of moderate × 5 + frequency of strenuous × 9  **Measurement unit**:  LSI (arbitrary units) | Examine the association between PA and health-related outcomes | Relative ranking | The case and control groups |
| MB Gilliam, A Madan-Swain, K Whelan, DC Tucker, W Demark-Wahnefried and DC Schwebel [81] | *N* = 105- USA  Mean age: 11  49% ♀  Type of cancer:  Leukemia, soft tissue sarcoma, CNS tumor, kidney tumor | Correlational- transversal | **Recall period**:  Typical week  **Asking for**:  Frequency and duration | **Scoring algorithm**:  Frequency of mild × 3 + frequency of moderate × 5 + frequency of strenuous × 9  Frequency × duration of mild PA  Frequency × duration of moderate PA  Frequency × duration of strenuous PA  **Measurement unit**:  LSI (arbitrary units)  Minutes/week | Identify correlates of PA | Relative ranking | Cancer-related/medical variables  Neighbourhood environment  Social support  Socio-demographic variables |
| MB Gilliam, A Madan-Swain, K Whelan, DC Tucker, W Demark-Wahnefried and DC Schwebel [82] | *N* = 105- USA  Mean age: 11  49% ♀  Type of cancer:  Leukemia, soft tissue sarcoma, CNS tumor, kidney tumor | Correlational- transversal | **Recall period**:  Typical week  **Asking for**:  Frequency and duration | **Scoring algorithm**:  Frequency × duration of mild  Frequency × duration of moderate  Frequency × duration of strenuous  **Measurement unit**:  Minutes/week | Identify correlates of PA | Relative ranking | Cancer-related/medical variables  Social cognitive theory constructs  Socio-demographic variables |
| GM Gjerset, SD Fosså, KS Courneya, E Skovlund, AB Jacobsen and L Thorsen [83] | *N* = 1284- Norway  Mean age: 57  56% ♀  36% completed college education  Type of cancer:  Testicular, breast, ovarian, cervical, prostate, lymphoma | Correlational- retrospective | **Recall period**:  Typical week-  a) Prior to cancer diagnosis  b) At the time of survey (post-diagnosis)  **Asking for**:  Frequency and duration | **Scoring algorithm**:  Meeting PA recommendations:  *Active*: ≥ 150 minutes of moderate/strenuous PA/week;  *Insufficiently active*: < 150 minutes of moderate/strenuous PA/week  **Measurement unit**:  Proportion (%) of *maintainers, adopters, inactive, relapsers* | Investigate interest and preferences for exercise | Classification | Interest in exercise counselling |
| GM Gjerset, SD Fosså, KS Courneya, E Skovlund and L Thorsen [84] | *N* = 975- Norway  Median age: 56  56% ♀  42% completed college education  Type of cancer:  Testicular, breast, ovarian, cervical, prostate, lymphoma | Correlational- retrospective | **Recall period**:  Typical week-  a) Prior to cancer diagnosis  b) At the time of survey (post-diagnosis)  **Asking for**:  Frequency and duration | **Scoring algorithm**:  Meeting PA recommendations:  *Active*: ≥ 150 minutes of moderate PA/week or ≥ 75 minutes of strenuous PA/week;  *Insufficiently active*: < 150 minutes of moderate PA/week and < 75 minutes of strenuous PA/week  **Measurement unit**:  Proportion (%) of *maintainers, adopters, persistently inactive, relapsers* | Assess the prevalence of PA  Assessment of PA patterns during cancer experience  Identify correlates of PA | Classification | Time  Cancer-related/medical variables  Socio-demographic variables |
| GM Gjerset, SD Fosså, AA Dahl, JH Loge, T Ensby and L Thorsen [85] | *N* = 67- Norway  Mean age: 67  0% ♀  52% completed college education  Type of cancer:  Prostate | Pre-experimental | **Recall period**:  Typical week  **Asking for**:  Frequency and duration | **Scoring algorithm**:  Frequency × duration of mild  Frequency × duration of moderate  Frequency × duration of strenuous  Meeting PA recommendations:  *Active*: ≥ 150 minutes of moderate PA/week or ≥ 75 minutes of strenuous PA/week;  *Insufficiently active*: < 150 minutes of moderate PA/week and < 75 minutes of strenuous PA/week  **Measurement unit**:  Minutes/week  Proportion (%) of *active* and *insufficiently active* individuals | Outcome of a PA intervention | Relative ranking  Classification | Experimental conditions |
| C Grimmett, J Bridgewater, A Steptoe and J Wardle [86] | *N* = 495- UK  Mean age: 68  41% ♀  Type of cancer:  Colorectal | Correlational- transversal | **Recall period**:  Typical week  **Asking for**:  Frequency | **Scoring algorithm**:  Meeting PA recommendations:  *Active*: ≥ 5 bouts of moderate/ strenuous PA/week;  *Insufficiently active*: < 5 bouts of moderate/ strenuous PA/week  **Measurement unit**:  Proportion (%) of *active* and *insufficiently active* individuals | Assess the prevalence of PA  Examine the association between PA and health-related outcomes | Classification | Fatigue  Quality of life |
| P Grossman, G Deuring, SN Garland, TS Campbell and LE Carlson [87] | *N* = 33- Canada  Mean age: 52  100% ♀  64% completed college education  Type of cancer:  Breast | Case-control | **Recall period**:  Not specified  **Asking for**:  Frequency | **Scoring algorithm**:  Frequency of mild × 3 + frequency of moderate × 5 + frequency of strenuous × 9  **Measurement unit**:  LSI (arbitrary units) | Examine the association between PA and health-related outcomes | Relative ranking | The case and control groups |
| E Guinan, J Hussey, JM Broderick, FE Lithander, D O'Donnell, MJ Kennedy and EM Connolly [88] | *N* = 26- Ireland  Mean age: 48  100% ♀  Type of cancer:  Breast | Truly experimental | **Recall period**:  Not specified  **Asking for**:  Frequency | **Scoring algorithm**:  Frequency of mild × 3 + frequency of moderate × 5 + frequency of strenuous × 9  **Measurement unit**:  LSI (arbitrary units) | Assessment of baseline equivalence of the treatment and comparison groups  Outcome of an intervention | Relative ranking | Experimental conditions |
| JM Harrington, DC Schwenke and DR Epstein [89] | *N* = 135- USA  Mean age: 72  0% ♀  Type of cancer:  Prostate | Correlational-transversal | **Recall period**:  Last week  **Asking for**:  Frequency | **Scoring algorithm**:  Meeting PA recommendations:  *Active*: ≥ 150 minutes of moderate/strenuous PA/week;  *Insufficiently active*: < 150 minutes of moderate/ strenuous PA/week  **Measurement unit**:  Proportion (%) of *active* and *insufficiently active* individuals | Determine exercise type, counselling, or programming preferences | Classification | BMI  Duration  of ADT  Physical activity preferences |
| AL Hawkes, SK Chambers, KI Pakenham, TA Patrao, PD Baade, BM Lynch, JF Aitken, X Meng and KS Courneya [90] | *N* = 410- Australia  Mean age: 66  54% ♀  91% completed high school education  Type of cancer:  Colorectal | Truly experimental | **Recall period**:  Not specified  **Asking for**:  Frequency and duration | **Scoring algorithm**:  Frequency × duration of mild  Frequency × duration of moderate  Frequency × duration of strenuous  Meeting PA recommendations:  *Active*: ≥ 150 minutes of moderate/strenuous PA/week;  *Insufficiently active*: < 150 minutes of moderate/ strenuous PA/week  **Measurement unit**:  Minutes/week  Proportion (%) of *active* and *insufficiently active* individuals | Assess eligibility criterion  Outcome of an intervention | Relative ranking  Classification | Experimental conditions |
| MC Hocking, LA Schwartz, WL Hobbie, BW Derosa, RF Ittenbach, JJ Mao, JP Ginsberg and AE Kazak [91] | *N* = 117- USA  Mean age: 22  53% ♀  37% completed college education  Type of cancer:  Breast | Case-control | **Recall period**:  Not specified  **Asking for**:  Frequency | **Scoring algorithm**:  Frequency of mild × 3 + frequency of moderate × 5 + frequency of strenuous × 9  Frequency of mild + frequency of moderate + frequency of strenuous  **Measurement unit**:  LSI (arbitrary units)  Number of LTPA bouts (frequency/week) | Identify correlates of PA | Relative ranking | Cancer-related/medical variables  Cognitive competences  Health beliefs  Socio-demographic variables |
| WE Hornsby, PS Douglas, MJ West, AA Kenjale, AR Lane, ER Schwitzer, KA Ray, JE Herndon, A Coan, A Gutierrez, et al. [92] | *N* = 20- USA  Mean age: 49  100% ♀  Type of cancer:  Breast | Truly experimental | **Recall period**:  Not specified  **Asking for**:  Frequency and duration | **Scoring algorithm**:  Frequency × duration of mild + frequency × duration of moderate + frequency × duration of strenuous  Frequency × duration of moderate + frequency × duration of strenuous  **Measurement unit**:  Minutes/week | Outcome of an intervention | Relative ranking | Experimental conditions |
| N Humpel and DC Iverson [93] | *N* = 91- Australia  Mean age: 61  35% ♀  31% completed graduate education  Type of cancer:  Prostate and breast | Correlational- retrospective | **Recall period**:  Typical week-  a) During 6 months prior to cancer diagnosis  b) At the time of survey  **Asking for**:  Frequency and duration | **Scoring algorithm**:  Meeting PA recommendations:  *Active*: ≥ 150 minutes of moderate/strenuous PA/week;  *Insufficiently active*: < 150 (but > 0) minutes of moderate/ strenuous PA/week;  *no PA*: 0 minutes of moderate/ strenuous PA/week  **Measurement unit**:  Proportion (%) of *active, insufficiently active* and *no PA* individuals | Examine the association between PA and health-related outcomes  Assessment of PA patterns during cancer experience  Identify correlates of PA | Classification | Body mass index  Cancer site  Depression  Quality of life  Time |
| N Humpel and DC Iverson [94] | *N* = 91- Australia  Mean age: 61  35% ♀  31% completed graduate education  Type of cancer:  Prostate and breast | Correlational- transversal | **Recall period**:  Typical week  **Asking for**:  Frequency and duration | **Scoring algorithm**:  Meeting PA recommendations:  *Active*: ≥ 150 minutes of mild/moderate/strenuous PA/week;  *Insufficiently active*: < 150 (but > 0) minutes of mild/moderate/ strenuous PA/week;  *No PA*: 0 minutes of mild/moderate/ strenuous PA/week  **Measurement unit**:  Proportion (%) of *active, insufficiently active* and *no PA* individuals | Examine the association between PA and health-related outcomes | Classification | Fatigue  Sleep |
| TT Hunt-Shanks, CM Blanchard, F Baker, D Hann, CS Roberts, J McDonald, M Livingston, C Witt, J Ruiterman, R Ampela, et al. [95] | *N* = 208- USA  Mean age: 62  61% ♀  40% completed college education  Type of cancer:  Prostate and breast | Correlational- transversal | **Recall period**:  Typical week-Since cancer diagnosis  **Asking for**:  Frequency | **Scoring algorithm**:  Frequency of mild + frequency of moderate + frequency of strenuous  **Measurement unit**:  Number of LTPA bouts (frequency)/week | Identify correlates of PA | Relative ranking | Theory of planned behavior constructs |
| PB Jacobsen, KM Phillips, HS Jim, BJ Small, LA Faul, CD Meade, L Thompson, CC Williams, Jr., LS Loftus, M Fishman, et al. [96] | *N* = 286- USA  Mean age: 58  68% ♀  40% completed college education  Type of cancer:  Breast, lung and other cancers | Truly experimental | **Recall period**:  Last week-  **Asking for**:  Frequency | **Scoring algorithm**:  Frequency of mild × 3 + frequency of moderate × 5 + frequency of strenuous × 9  **Measurement unit**:  LSI (arbitrary units) | Assessment of baseline equivalence of the treatment and comparison groups  Test the difference in PA between study completers and drop-outs  Outcome of an intervention | Relative ranking | Experimental conditions |
| LM Jaremka, RR Andridge, CP Fagundes, CM Alfano, SP Povoski, AM Lipari, DM Agnese, MW Arnold, WB Farrar, LD Yee, et al. [97] | *N* = 49- USA  (Study-1)  Mean age: 58  98% ♀  78% completed college/university  Type of cancer:  Breast and colorectal | Correlational-longitudinal | **Recall period**:  Last week  **Asking for**:  Frequency and duration | **Scoring algorithm**:  MVPA: Frequency × duration of moderate + frequency × duration of strenuous  **Measurement unit**:  Minutes/week | Examine the association between PA and health-related outcomes | Relative ranking | Loneliness  Symptoms cluster (depression, fatigue, and pain) |
| M Jeffreys, F McKenzie, R Firestone, M Gray, S Cheng, A Moala, N Pearce and L Ellison-Loschmann [98] | *N* = 1799- New Zealand  Mean age: 60  100% ♀  52% completed high school  Type of cancer:  Breast | Case-control:  retrospective | **Recall period**:  Last year  **Asking for**:  Frequency | **Scoring algorithm**:  Frequency of mild × 3 + frequency of moderate × 5 + frequency of strenuous × 9  **Measurement unit**:  Quartile of LSI (arbitrary units) | Examine the association between PA and health-related outcomes | Classification | Risk of breast cancer |
| LW Jones, RR Cohen, SK Mabe, MJ West, A Desjardins, JJ Vredenburgh, AH Friedman, DA Reardon, E Waner and HS Friedman [99] | *N* = 171- USA  Mean age: 49  68% ♀  Type of cancer:  Brain (malignant glioma) | Correlational-  transversal | **Recall period**:  Last month  **Asking for**:  Frequency and duration | **Scoring algorithm**:  Frequency × duration of mild + frequency × duration of moderate + frequency × duration of strenuous  Meeting PA recommendations:  *Active*: ≥ 150 minutes of MVPA/week;  *Insufficiently active*: < 150 minutes of MVPA/week  **Measurement unit**: Minutes/week  Proportion (%) of *active* and *insufficiently active* individuals | Examine the association between PA and health-related outcomes | Relative ranking  Classification | Physical fitness indicators  (6-min walking distance) |
| LW Jones and KS Courneya [100] | *N* = 307- USA  Mean age: 61  59% ♀  47% completed college education  Type of cancer:  Breast, colorectal, lung, prostate | Correlational-  retrospective | **Recall period**:  Typical week-  During cancer treatment  **Asking for**:  Frequency and duration | **Scoring algorithm**:  Frequency of mild frequency of moderate frequency of strenuous  Frequency × duration of mild + frequency × duration of moderate + frequency × duration of strenuous  Meeting PA recommendations:  *Active*: ≥ 1 bout of moderate/strenuous PA/week;  *Insufficiently active*: 0 bout of moderate/ strenuous PA/week  **Measurement unit**:  Number of LTPA cessions (frequency)/week  Minutes/week  Proportion (%) of *active* and *insufficiently active* individuals | Determine exercise type, counselling, or programming preferences | Relative ranking  Classification | Exercise programming preferences |
| LW Jones and KS Courneya [101] | *N* = 303- USA  Mean age: 61  59% ♀  46% completed college education  Type of cancer:  Breast, colorectal, lung, prostate | Correlational-  retrospective | **Recall period**:  Typical week-  During the total span of the cancer treatment  **Asking for**:  Frequency and duration | **Scoring algorithm**:  Frequency of mild × 3 + frequency of moderate × 5 + frequency of strenuous × 9  Frequency × duration of mild + frequency × duration of moderate + frequency × duration of strenuous  Meeting PA recommendations:  *Active*: ≥ 150 minutes of moderate/strenuous PA/week;  *Insufficiently active*: < 150 minutes of moderate/ strenuous PA/week  **Measurement unit**:  LSI (arbitrary units)  Minutes/week  Proportion (%) of *active* and *insufficiently active* individuals | Identify correlates of PA  Determine exercise type, counselling modalities preferences | Relative ranking  Classification | Exercise discussion with oncologist |
| LW Jones, KS Courneya, AS Fairey and JR Mackey [102] | *N* = 329- Canada  Mean age: 56  100% ♀  Type of cancer:  Breast | Truly experimental | **Recall period**:  Typical week-  During treatment  **Asking for**:  Frequency and duration | **Scoring algorithm**:  Frequency of mild × 3 + frequency of moderate × 5 + frequency of strenuous × 9  Frequency × duration of mild + frequency × duration of moderate + frequency × duration of strenuous  Frequency × duration of mild × 3 + frequency × duration of moderate × 5 + frequency × duration of strenuous × 9  Meeting PA recommendations:  *Active*: ≥ 150 minutes of moderate PA/week  *Insufficiently active*: < 150 minutes of moderate PA/week  **Measurement unit**:  LSI (arbitrary units)  Minutes/week  METs × hours/week  Proportion (%) of *active* and *insufficiently active* Individuals | Outcome of an intervention | Relative ranking  Classification | Experimental conditions |
| LW Jones, KS Courneya, AS Fairey and JR Mackey [103] | *N* = 450- Canada  Mean age: 62  100% ♀  Type of cancer:  Breast | Truly experimental | **Recall period**:  Typical week  **Asking for**:  Frequency and duration | **Scoring algorithm**:  Frequency × duration of mild × 3  Frequency × duration of moderate × 5  Frequency × duration of strenuous × 9  **Measurement unit**:  METs × hours/week | Identify correlates of PA (mediation analysis)  Outcome of an intervention | Relative ranking | Experimental conditions  Theory of planned behaviour constructs |
| LW Jones, KS Courneya, JR Mackey, HB Muss, EN Pituskin, JM Scott, WE Hornsby, AD Coan, JE Herndon, II, PS Douglas, et al. [104] | *N* = 248- Canada/USA  Mean age: 55  100% ♀  Type of cancer:  Breast | Correlational-  Transversal | **Recall period**:  Not specified  **Asking for**:  Frequency and duration | **Scoring algorithm**:  Frequency × duration of mild +  Frequency × duration of moderate +  Frequency × duration of strenuous  Meeting PA recommendations:  *Active*: ≥ 150 minutes of MVPA/week;  *Insufficiently active*: < 150 minutes of MVPA/week  **Measurement unit**: Minutes/week  Proportion (%) of *active* and *insufficiently active* individuals | Describe study sample in terms of LTPA | Relative ranking  Classification | N/A |
| LW Jones, KS Courneya, JKH Vallance, AB Ladha, MJ Mant, AR Belch and T Reiman [105] | *N* = 70- Canada  Mean age: 64  44% ♀  52% completed college education  Type of cancer:  Multiple myeloma | Correlational-  Retrospective | **Recall period**:  Typical week-  a) Before diagnosis  b) During treatment  c) After treatment  **Asking for**:  Frequency and duration | **Scoring algorithm**:  Frequency × duration of mild + frequency × duration of moderate + frequency × duration of strenuous  **Measurement unit**: Minutes/week | Identify correlates of PA | Relative ranking | Cancer-related/medical variables  Socio-demographic variables  Theory of Planned behaviour constructs |
| LW Jones, KS Courneya, JKH Vallance, AB Ladha, MJ Mant, AR Belch, DA Stewart and T Reiman [106] | *N* = 88- Canada  Mean age: 64  59% ♀  49% completed college education  Type of cancer:  Multiple myeloma | Correlational-  Retrospective | **Recall period**:  Typical week-  a) Before diagnosis  b) During treatment  c) After treatment  **Asking for**:  Frequency and duration | **Scoring algorithm**:  Frequency × duration of mild + frequency × duration of moderate + frequency × duration of strenuous  Meeting PA recommendations:  *Active*: ≥ 150 minutes of MVPA/week;  *Insufficiently active*: < 150 minutes of MVPA/week  **Measurement unit**: Minutes/week  Proportion (%) of *active* and *insufficiently active* individuals | Assess prevalence of PA  Assessment of PA patterns during cancer experience  Examine the association between PA and health-related outcomes | Relative ranking  Classification | Anemia  Depression  Fatigue  Quality of life  Wellbeing |
| LW Jones, ND Eves, JR Mackey, CJ Peddle, M Haykowsky, AA Joy, KS Courneya, K Tankel, J Spratlin and T Reiman [107] | *N* = 85- Canada  Mean age: 63  35% ♀  Type of cancer:  Lung | Correlational-  Transversal | **Recall period**:  Typical week-  Since diagnosis  **Asking for**:  Frequency and duration | **Scoring algorithm:**  Not specified  **Measurement unit**:  Minutes/week | Assess the prevalence of PA | Relative ranking | N/A |
| LW Jones, AH Friedman, MJ West, SK Mabe, J Fraser, WE Kraus, HS Friedman, MI Tresch, N Major and DA Reardon [108] | *N* = 35- USA  Mean age: 47  40% ♀  Type of cancer:  Brain (malignant glioma) | Correlational-  Transversal | **Recall period**:  Last month  **Asking for**:  Frequency and duration | **Scoring algorithm**:  Frequency × duration of mild + frequency × duration of moderate + frequency × duration of strenuous  **Measurement unit**: Minutes/week | Examine the association between PA and health-related outcomes | Relative ranking | Fatigue  Quality of life  Muscle cross-sectional area  VO2peak |
| LW Jones, B Guill, ST Keir, K Carter, HS Friedman, DD Bigner and DA Reardon [109] | *N* = 106- USA  Mean age: 45  51% ♀  82% completed college education  Type of cancer:  Brain | Correlational-  Retrospective | **Recall period**:  Typical week-  a) Before diagnosis  b) During treatment  c) After treatment  **Asking for**:  Frequency and duration | **Scoring algorithm**:  Frequency × duration of mild + frequency × duration of moderate + frequency × duration of strenuous  Frequency × duration of mild; moderate; and strenuous  Meeting PA recommendations:  *Active*: ≥ 150 minutes of moderate/strenuous PA/week;  *Insufficiently active*: < 150 (but > 0) minutes of moderate/ strenuous PA/week  *No PA*: 0 minutes of moderate/ strenuous PA/week  **Measurement unit**:  Minutes/week  Proportion (%) of *active, insufficiently active* and *no PA individuals* | Assessment of PA patterns during cancer experience  Identify correlates of PA | Relative ranking  Classification | Cancer-related/medical variables  Socio-demographic variables |
| LW Jones, B Guill, ST Keir, K Carter, HS Friedman, DD Bigner and DA Reardon [110] | *N* = 170- USA  Mean age: 45  51% ♀  82% completed college education  Type of cancer:  Brain | Correlational-  Retrospective | **Recall period**:  Typical week-  a) Before diagnosis  b) During treatment  c) After treatment  **Asking for**:  Frequency and duration | **Scoring algorithm**:  Frequency × duration of mild + frequency × duration of moderate + frequency × duration of strenuous  Meeting PA recommendations:  *Active*: ≥ 150 minutes of MVPA/week;  *Insufficiently active*: < 150 minutes of MVPA/week  **Measurement unit**: Minutes/week  Proportion (%) of *active* and *insufficiently active* individuals | Determine exercise type, counselling, and programming preferences | Relative ranking  Classification | Exercise programming preferences |
| LW Jones, B Guill, ST Keir, K Carter, HS Friedman, DD Bigner and DA Reardon [111] | *N* = 100- USA  Mean age: 45  51% ♀  85% completed college education  Type of cancer:  Brain | Correlational-  retrospective | **Recall period**:  Typical week-  a) Before diagnosis  b) During treatment  c) After treatment  **Asking for**:  Frequency and duration | **Scoring algorithm**:  Frequency × duration of mild + frequency × duration of moderate + frequency × duration of strenuous  Meeting PA recommendations:  *Active*: ≥ 150 minutes of MVPA/week;  *Insufficiently active*: < 150 minutes of MVPA/week  **Measurement unit**: Minutes/week  Proportion (%) of *active* and *insufficiently active* individuals | Identify correlates of PA intention | Relative ranking  Classification | Cancer-related/medical variables  Socio-demographic variables  Theory of Planned behavior constructs |
| LW Jones, WE Hornsby, A Goetzinger, LM Forbes, EL Sherrard, M Quist, AT Lane, M West, ND Eves, M Gradison, et al. [112] | *N* = 118- USA  Mean age: 61  40% ♀  Type of cancer:  Lung | Correlational-  longitudinal | **Recall period**:  Typical week-  Since cancer treatment  **Asking for**:  Frequency and duration | **Scoring algorithm**:  Frequency × duration of mild + frequency × duration of moderate + frequency × duration of strenuous  Frequency × duration of mild × 3 + frequency × duration of moderate × 5 + frequency × duration of strenuous × 9  Meeting PA recommendations:  *Active*: ≥ 150 minutes of moderate/strenuous PA/week;  *Insufficiently active*: < 150 (but > 0) minutes of moderate/ strenuous PA/week;  *No PA*: 0 minutes of mild, moderate, or strenuous PA/week  **Measurement unit**:  Minutes/week  METs × hours/week  Proportion (%) of *active*, *insufficiently active* *and no PA at all* individuals | Examine the association between PA and health-related outcomes | Relative ranking  Classification | Survival time |
| LW Jones, M Mourtzakis, KB Peters, AH Friedman, MJ West, SK Mabe, WE Kraus, HS Friedman and DA Reardon [113] | *N* = 100- USA  Mean age: 47  40% ♀  Type of cancer:  Brain (malignant glioma) | Correlational-  longitudinal | **Recall period**:  Last month-  a) Baseline  b) 6 weeks after baseline  c) 24 weeks after treatment  **Asking for**:  Frequency and duration | **Scoring algorithm**:  Frequency × duration of mild + frequency × duration of moderate + frequency × duration of strenuous  **Measurement unit**: Minutes/week | Assessment of PA patterns during cancer experience  Examine the association between PA and health-related outcomes | Relative ranking | Time  Quality of life |
| LW Jones, CJ Peddle, ND Eves, MJ Haykowsky, KS Courneya, JR Mackey, AA Joy, V Kumar, TW Winton and T Reiman [114] | *N* = 20- Canada  Mean age: 65  70% ♀  Type of cancer:  Lung | Single group; pre-experimental | **Recall period**:  Not specified  **Asking for**:  Frequency and duration | **Scoring algorithm**:  Frequency × duration of mild + frequency × duration of moderate + frequency × duration of strenuous  **Measurement unit**: Minutes/week | Served as a control variable in the analyses  Assess LTPA performed outside the supervised PA program | Relative ranking | Experimental conditions |
| KH Karvinen, KS Courneya, KL Campbell, RG Pearcey, G Dundas, V Capstick and KS Tonkin [115] | *N* = 386- Canada  Mean age: 65  100% ♀  38% completed college education  Type of cancer:  Endometrial | Correlational-  transversal | **Recall period**:  Last month  **Asking for**:  Frequency and duration | **Scoring algorithm**:  Meeting PA recommendations:  *Active*: ≥ 150 minutes of Moderate PA/week or : ≥ 60 minutes of strenuous PA/week;  *Insufficiently active*: < 150 minutes of moderate PA/week and < 60 minutes of strenuous PA/week  **Measurement unit**:  Proportion (%) of *active, insufficiently active* individuals | Investigate exercise programming and counselling preferences | Classification | Exercise programming and exercise counselling preferences |
| KH Karvinen, KS Courneya, KL Campbell, RG Pearcey, G Dundas, V Capstick and KS Tonkin [116] | *N* = 354- Canada  Mean age: 65  100% ♀  38% completed college education  Type of cancer:  Endometrial | Correlational-  transversal | **Recall period**:  Not specified  **Asking for**:  Frequency and duration | **Scoring algorithm**:  Meeting PA recommendations:  *Active*: ≥ 150 minutes of moderate PA/week or : ≥ 60 minutes of strenuous PA/week;  *Insufficiently active*: < 150 minutes of moderate PA/week and < 60 minutes of strenuous PA/week; S*edentary*: 0 minute of moderate and strenuous PA/week  **Measurement unit**:  Proportion (%) of *active, insufficiently active* and *sedentary* individuals | Identify correlates of PA | Classification | Cancer-related/medical variables  Socio-demographic variables  Theory of Planned behavior constructs |
| KH Karvinen, KS Courneya, S North and P Venner [117] | *N* = 525- Canada  Mean age: 70  25% ♀  20% completed college education  Type of cancer:  Bladder | Correlational-  retrospective | **Recall period**:  Past month-  a) Before diagnosis  b) During treatment  c) After treatment  **Asking for**:  Frequency and duration | **Scoring algorithm**:  Meeting PA recommendations:  *Active*: ≥ 150 minutes of moderate PA/week or ≥ 60 minutes of strenuous PA/week;  *Insufficiently active*: < 150 minutes of moderate PA/week and < 60 minutes of strenuous PA/week; S*edentary*: 0 minute of moderate and strenuous PA/week  **Measurement unit**:  Proportion (%) of *active, insufficiently active* and *sedentary* individuals;  Proportion (%) of *maintainers, temporary relapsers, permanent relapsers and non-exercisers.* | Assessment of PA patterns during cancer experience  Examine the association between PA and health-related outcomes | Classification | Fatigue  Quality of life  Time |
| KH Karvinen, KS Courneya, RC Plotnikoff, JC Spence, PM Venner and S North [118] | *N* = 525- Canada  Mean age: 70  75% ♀  Type of cancer:  Bladder | Correlational-  longitudinal | **Recall period**:  Last 3 months  **Asking for**:  Frequency and duration | **Scoring algorithm**:  Meeting PA recommendations:  *Active*: ≥ 150 minutes of moderate PA/week or ≥ 60 minutes of strenuous PA/week;  *Insufficiently active*: < 150 minutes of moderate PA/week and < 60 minutes of strenuous PA/week;  *No LTPA*: 0 minute of moderate and strenuous PA/week  **Measurement unit**:  Proportion (%) of *active, insufficiently active,* and *No* *LTPA* individuals | Identify correlates of PA | Classification | Clinical/medical variables  Socio-demographic variables  Theory of planned behavior constructs |
| KH Karvinen, KS Courneya, P Venner and S North [119] | *N* = 525- Canada  Median age: 65  26% ♀  38% completed college education  Type of cancer:  Bladder | Correlational-  longitudinal | **Recall period**:  Last month  **Asking for**:  Frequency and duration | **Scoring algorithm**:  Meeting PA recommendations:  *Active*: ≥ 150 minutes of moderate PA/week or ≥ 60 minutes of strenuous PA/week;  *Insufficiently active*: < 150 minutes of moderate PA/week and < 60 minutes of strenuous PA/week  **Measurement unit**:  Proportion (%) of *active* and *insufficiently active* individuals | Investigate exercise programming and counselling preferences | Classification | Exercise programming and exercise counselling preferences |
| KH Karvinen, TD Raedeke, H Arastu and RR Allison [120] | *N* = 91- Canada  Mean age: 57  100% ♀  62% completed college education  Type of cancer:  Breast | Correlational-  transversal | **Recall period**:  Last months  **Asking for**:  Frequency and duration | **Scoring algorithm**:  Meeting PA recommendations:  *Active*: ≥ 150 minutes of moderate PA/week or : ≥ 60 minutes of strenuous PA/week;  *Insufficiently active*: < 150 minutes of moderate PA/week and < 60 minutes of strenuous PA/week  **Measurement unit**:  Proportion (%) of *active* and *insufficiently active* individuals | Assess PA prevalence  Identify correlates of PA  Investigate exercise programming and counselling preferences | Classification | Exercise programming and exercise counselling preferences  Socio-demographic variables |
| KH Karvinen, D Esposito, TD Raedeke, J Vick and PR Walker [121] | *N* = 14- USA  Mean age: 57  29% ♀  21% had some post-secondary school  Type of cancer:  Lung | Truly experimental | **Recall period**:  Last months  **Asking for**:  Frequency and duration | **Scoring algorithm**:  Frequency × duration of moderate + frequency × duration of strenuous × 2  Frequency × duration of moderate  Frequency × duration of strenuous  Meeting PA recommendations:  *Active*: ≥ 150 minutes of MVPA/week or ≥ 60 minutes of strenuous PA/week;  *Insufficiently active*: < 150 minutes of MVPA/week and < 60 minutes of strenuous PA/week  **Measurement unit**:  Minutes/week  Proportion (%) of *active* and *insufficiently active* | Assessment of baseline equivalence of the treatment and comparison groups | Relative ranking  Classification | Experimental conditions |
| MR Keats, KS Courneya, S Danielsen and SF Whitsett [122] | *N* = 53- Canada  Mean age: 17  47% ♀  Type of cancer:  Lymphoma, leukemia, central nervous system | Correlational-retrospective | **Recall period**:  Typical week-  a) Before diagnosis  b) During treatment  c) After treatment  **Asking for**:  Frequency | **Scoring algorithm**:  Frequency of mild × 3 + frequency of moderate × 5 + frequency of strenuous × 9  Meeting PA recommendations:  *Active*: ≥ 27 LSI; *Insufficiently active*: < 27 LSI  **Measurement unit**:  LSI (arbitrary units)    Proportion (%) of *active* and *insufficiently active* individuals; Proportion (%) of *maintainers, temporary relapsers, permanent relapsers* and *nonparticipants* individuals | Assessment of PA patterns during cancer experience  Examine the association between PA and health-related outcomes | Relative ranking  Classification | Well-being  Time |
| MR Keats and N Culos-Reed [123] | *N* = Not specified- Canada  Mean age: 16  80% ♀  Type of cancer:  Lymphoma, leukemia, central nervous system, germ cell tumor | Correlational-  longitudinal | **Recall period**:  Typical week  **Asking for**:  Frequency | **Scoring algorithm**:  Frequency of mild × 3 + frequency of moderate × 5 + frequency of strenuous × 9  **Measurement unit**:  LSI (arbitrary units) | Identify correlates of PA | Relative ranking | Theory of Planned behavior constructs |
| MR Keats and SN Culos-Reed [124] | *N* = 10- Canada  Mean age: 16  80% ♀  Type of cancer:  Lymphoma, leukemia, central nervous system | Pre-experimental | **Recall period**:  Not specified  **Asking for**:  Frequency and duration | **Scoring algorithm**:  Frequency × duration of mild PA × 3  Frequency × duration of moderate PA × 5  Frequency × duration of strenuous PA × 9  **Measurement unit**:  METs × hours/week | Outcome of an intervention | Relative ranking | Experimental conditions |
| MR Keats, SN Culos-Reed, KS Courneya and M McBride [125] | *N* = 97- Canada  Mean age: 17  43% ♀  Type of cancer:  Lymphoma, leukemia, central nervous system | Correlational-  retrospective | **Recall period**:  Typical week-  a) Before diagnosis  b) During treatment  c) After treatment  **Asking for**:  Frequency | **Scoring algorithm**:  Frequency of mild; frequency of moderate; frequency of strenuous.  Frequency of mild × 3 + frequency of moderate × 5 + frequency of strenuous × 9  Meeting PA recommendations:  *Active*: ≥ 27 LSI; *Insufficiently active*: < 27 LSI  **Measurement unit**:  LSI (arbitrary units)  Number of LTPA cessions (frequency)/week  Proportion (%) of *maintainers*, *temporary relapsers*, *permanent relapsers* | Assessment of PA patterns during cancer experience | Relative ranking  Classification | Time |
|  |  |  |  |  |  |  |  |
| MR Keats, SN Culos-Reed, KS Courneya and M McBride [126] | *N* = 59- Canada  Mean age: 17  39% ♀  Type of cancer:  Lymphoma, leukemia, central nervous system | Correlational-  retrospective | **Recall period**:  Typical week-  a) Before diagnosis  b) During treatment  c) After treatment  **Asking for**:  Frequency and duration | **Scoring algorithm**:  Frequency of mild; frequency of moderate; frequency of strenuous.  Frequency of mild × 3 + frequency of moderate × 5 + frequency of strenuous × 9  Frequency × duration of mild PA × 3  Frequency × duration of moderate PA × 5  Frequency × duration of strenuous PA × 9  **Measurement unit**:  Number of LTPA cessions (frequency)/week  LSI (arbitrary units)  METs × hours/week | Identify correlates of PA | Relative ranking | Theory of Planned behavior constructs |
| AA Kenjale, WE Hornsby, T Crowgey, S Thomas, JE Herndon Ii, MG Khouri, AR Lane, CE Bishop, ND Eves, J Peppercorn, et al. [127] | *N* = 413- USA  Mean age: 58  61% ♀  Type of cancer:  Breast, glioma, hematological, lung | Correlational-transversal | **Recall period**:  Not specified  **Asking for**:  Frequency and duration | **Scoring algorithm**:  Meeting PA recommendations:  *Active*: ≥ 150 minutes of MVPA/week minutes of strenuous PA/week;  *Insufficiently active*: < 150 minutes of MVPA/week  **Measurement unit**:  Proportion (%) of *active* and *insufficiently active* individuals | Describe study sample in terms of LTPA | Classification | N/A |
| P Krebs, EJ Coups, MB Feinstein, JE Burkhalter, RM Steingart, A Logue, BJ Park and JS Ostroff [128] | *N* = 183- USA  Mean age: 69  64% ♀  47% completed college education  Type of cancer:  Lung | Correlational-  transversal | **Recall period**:  Not specified  **Asking for**:  Frequency and duration | **Scoring algorithm**:  Meeting PA recommendations:  *Active*: ≥ 150 minutes of MVPA/week;  *Insufficiently active*: < 150 minutes of MVPA/week  **Measurement unit**:  Proportion (%) of *active, insufficiently active* individuals | Assess the prevalence of PA | Classification | N/A |
| AB Ladha, KS Courneya, GJ Bell, CJ Field and P Grundy [129] | *N* = 4- Canada  Mean age: 11  0% ♀  Type of cancer:  Leukemia | Case-control | **Recall period**:  Not specified  **Asking for**:  Frequency and duration | **Scoring algorithm**:  Frequency × duration of mild + frequency × duration of moderate + frequency × duration of strenuous  **Measurement unit**: Minutes/week | Examine the association between PA and health-related outcomes | Relative ranking | The case and control groups |
| YY Lin, YC Wu, KM Rau and CC Lin [130] | *N* = 185- Taiwan  Mean age: 64  48% ♀  20% completed college education  Type of cancer:  Lung | Correlational-  transversal | **Recall period**:  Typical week  **Asking for**:  Frequency and duration | **Scoring algorithm**:  Meeting PA recommendations:  *Active*: ≥ 150 minutes of MVPA/week;  *Insufficiently active*: < 150 minutes of MVPA/week; *Light activity:* any mild LTPA vs. no mild LTPA; *Sedentary*: no mild, moderate and strenuous LTPA vs any LTPA.  **Measurement unit**:  Proportion (%) of *active, insufficiently active, light activity* and *sedentary* individuals | Examine the association between PA and health-related outcomes | Classification | Quality of life |
| YY Lin, MF Liu, JI Tzeng and CC Lin [131] | *N* = 107- Taiwan  Mean age: 64  47% ♀  Mean year of education : 9 yr.  Type of cancer:  Lung | Correlational-longitudinal | **Recall period**:  Typical week  **Asking for**:  Frequency and duration | **Scoring algorithm**:  Frequency × duration of walking  **Measurement unit**:  Frequency/week of walking  Minutes/week of walking | Examine the association between PA and health-related outcomes  Identify correlates of PA | Relative ranking | Quality of life  Self-efficacy  Social support |
| C Love and CM Sabiston [132] | *N* = 64- Canada  Mean age: 29  73% ♀  21% completed undergrad education  Type of cancer:  Carcinoma, lymphoma, leukemia, sarcoma, central nervous system skin | Correlational-  transversal | **Recall period**:  Typical week  **Asking for**:  Frequency and duration | **Scoring algorithm**:  Meeting PA recommendations:  *Active*: ≥ 150 minutes of MVPA/week;  *Insufficiently active*: < 150 minutes of MVPA/week  **Measurement unit**:  Proportion (%) of *active, insufficiently active* individuals | Examine the association between PA and health-related outcomes | Classification | Posttraumatic growth  Social support  Stress |
| DE Mack, LS Meldrum, PM Wilson and CM Sabiston [133] | *N* = 144- Canada  Mean age: 55  100% ♀  Type of cancer:  Breast | Correlational-  longitudinal | **Recall period**:  Typical week  **Asking for**:  Frequency | **Scoring algorithm**:  Frequency of mild × 3 + frequency of moderate × 5 + frequency of strenuous × 9  **Measurement unit**:  LSI (arbitrary units) | Examine the association between PA and health-related outcomes | Relative ranking | Affect  Depressive symptoms  Psychological Needs Theory constructs  Stress |
| MJ Mackenzie, LE Carlson, P Ekkekakis, DM Paskevich and SN Culos-Reed [134] | *N* = 66- Canada  Mean age: 53  90% ♀  55% completed college education  Type of cancer:  Breast, colorectal, lymphoma, other types of cancer | Pre-experimental | **Recall period**:  Typical week-  Last month  **Asking for**:  Frequency | **Scoring algorithm**:  Frequency of mild × 3 + frequency of moderate × 5 + frequency of strenuous × 9  **Measurement unit**:  LSI (arbitrary units) | Examine the association between PA and health-related outcomes  Outcome of an intervention | Relative ranking | PA program  Affect  Mindfulness  Mood  Quality of life  Stress  Time |
| MJ Mackenzie, LE Carlson, DM Paskevich, P Ekkekakis, AJ Wurz, K Wytsma, KA Krenz, E McAuley and SN Culos-Reed [135] | *N* = 18- Canada  Mean age: 54  100% ♀  61% completed college/university  Type of cancer:  61% had breast cancer | Correlational-transversal | **Recall period**:  Typical week-  Last month  **Asking for**:  Frequency and duration | **Scoring algorithm**:  Frequency of mild × 3 + frequency of moderate × 5 + frequency of strenuous × 9  Frequency × duration of moderate + frequency × duration of strenuous  **Measurement unit**:  LSI (arbitrary units)  Minutes/week | Examine the association between PA and health-related outcomes | Relative ranking | Affect  Attention  Perceived exertion |
| M Maddocks, S Armstrong and A Wilcock [136] | *N* = 200- UK  Mean age: 64  52% ♀  Type of cancer:  Breast, colorectal, lung, urological and gynecological, upper gastro-intestinal cancers | Correlational-  transversal | **Recall period**:  Typical week-  a) Before diagnosis  b) At the time of survey  **Asking for**:  Frequency | **Scoring algorithm**:  Frequency of mild × 3 + frequency of moderate × 5 + frequency of strenuous × 9  Meeting PA recommendations:  *Active*: ≥ 15 LSI; *Insufficiently active*: < 15 LSI  **Measurement unit**:  LSI (arbitrary units)  Proportion (%) of *active* and *insufficiently active* individuals | Investigate exercise programming and counselling preferences | Relative ranking  Classification | Exercise programming and counselling preferences |
| E McAuley, SM White, LQ Rogers, RW Motl and KS Courneya [137] -  Study 1 | *N* = 192- USA  Mean age: 64  100% ♀  Type of cancer:  Breast | Correlational-  transversal | **Recall period**:  Typical week  **Asking for**:  Frequency and duration | **Scoring algorithm**:  Frequency × duration of moderate LTPA + frequency × duration of strenuous LTPA  **Measurement unit**:  Minutes/week | Examine the association between PA and health-related outcomes | Relative ranking | Depression symptoms  Fatigue  Self-efficacy |
| EL McGowan, S North and KS Courneya [138] | *N* = 423- Canada  Mean age: 68  0% ♀  38% completed college education  Type of cancer:  Prostate | Truly experimental | **Recall period**:  Last month  **Asking for**:  Frequency and duration | **Scoring algorithm:**  Frequency × duration of mild;  Frequency × duration of moderate;  Frequency × duration of strenuous  **Scoring algorithm**:  Meeting PA recommendations:  *Active*: ≥ 150 minutes of moderate PA/week or ≥ 75 minutes of strenuous PA/week;  *Insufficiently active*: < 150 minutes of moderate PA/week and < 75 minutes of strenuous PA/week  **Measurement unit**:  Minutes/week  Proportion (%) of *active, insufficiently active* individuals | Assessment of baseline equivalence of the treatment and comparison groups  Outcome of an intervention | Relative ranking  Classification | Experimental conditions |
| EL McGowan, AE Speed-Andrews, CM Blanchard, RE Rhodes, CM Friedenreich, SN Cubs-Reed and KS Courneya [139] | *N* = 600- Canada  Median age: 65  42% ♀  51% completed college education  Type of cancer:  Colorectal | Correlational-  transversal | **Recall period**:  Last month  **Asking for**:  Frequency and duration | **Scoring algorithm**:  Meeting PA recommendations:  *Active*: ≥ 150 minutes of moderate PA/week or ≥ 75 minutes of strenuous PA/week;  *Insufficiently active*: < 150 minutes of moderate PA/week and < 75 minutes of strenuous PA/week  **Measurement unit**:  Proportion (%) of *active, insufficiently active* individuals | Investigate exercise programming and counselling preferences | Classification | Exercise programming and counselling preferences |
| F McKenzie, L Ellison-Loschmann, M Jeffreys, R Firestone, N Pearce and I Romieu [140] | *N* = 1799- New Zealand  Mean age: 60  100% ♀  52% completed high school  Type of cancer:  Breast | Case-control | **Recall period**:  Last year  **Asking for**:  Frequency | **Scoring algorithm**:  Frequency of mild × 3 + frequency of moderate × 5 + frequency of strenuous × 9  **Measurement unit**:  Quartile of LSI | Served as a control variable in the analyses | Classification | N/A |
| F McKenzie, L Ellison-Loschmann, M Jeffreys, R Firestone, N Pearce and I Romieu [141] | *N* = 1093- New Zealand  Mean age: 58  100% ♀  Type of cancer:  Breast | Case-control | **Recall period**:  Last year  **Asking for**:  Frequency | **Scoring algorithm**:  Frequency of moderate × 5 + frequency of strenuous × 9  Meeting PA recommendations:  *Active*: ≥ 36 LSI from MVPA;  *Moderately active*: 24-35 LSI from MVPA; *Insufficiently active*: < 24 LSI from MVPA  **Measurement unit**:  Proportion (%) of *active, moderately active* and *insufficiently active* | Include in a healthy lifestyle score; which was used to examine the association between PA and health-related outcomes | Classification | Cancer |
| ML McNeely, M Parliament, KS Courneya, H Seikaly, N Jha, R Scrimger and J Hanson [142] | *N* = 17- Canada  Mean age: 61  82% ♀  Type of cancer:  Head and neck cancers | Truly experimental | **Recall period**:  Last month  **Asking for**:  Frequency and duration | **Scoring algorithm**:  Frequency × duration of moderate;  Frequency × duration of strenuous  Meeting PA recommendations:  *Active*: ≥ 90 minutes of MVPA/week; *insufficiently active*: < 90 minutes of MVPA/week  **Measurement unit**:  Minutes/week  Proportion (%) of *active, insufficiently active* individuals | Assessment of baseline equivalence of the treatment and comparison groups | Relative ranking  Classification | Experimental conditions |
| RA Mesa, J Niblack, M Wadleigh, S Verstovsek, J Camoriano, S Barnes, AD Tan, PJ Atherton, JA Sloan and A Tefferi [143] | *N* = 1179- USA  Median age: 56  59% ♀  Type of cancer:  Hematological neoplasm | Correlational-transversal | **Recall period**:  Typical week  **Asking for**:  Frequency | **Scoring algorithm**:  Frequency of mild × 3 + frequency of moderate × 5 + frequency of strenuous × 9  **Measurement unit**:  LSI (arbitrary units) | Identify correlates of PA | Relative ranking | PA barriers |
| HM Milne, S Gordon, A Guilfoyle, KE Wallman and KS Courneya [144] | *N* = 558- Australia  Mean age: 59  100% ♀  34% completed college education  Type of cancer:  Breast | Correlational-retrospective | **Recall period**:  Typical week-  a) Before diagnosis  b) During treatment  c) After treatment  **Asking for**:  Frequency and duration | **Scoring algorithm**:  Frequency × duration of moderate + frequency × duration of strenuous  Frequency × duration of mild; moderate; and strenuous  Meeting PA recommendations:  *Active*: ≥ 150 minutes of moderate PA/week or ≥ 60 minutes of strenuous PA/week;  *Insufficiently active*: < 150 minutes of moderate PA/week and < 60 minutes of strenuous PA/week  **Measurement unit**:  Minutes/week  Proportion (%) of *active, insufficiently active* individuals | Assessment of PA patterns during cancer experience  Examine the association between PA and health-related outcomes | Relative ranking  Classification | Body mass index  Quality of life  Time |
| HM Milne, KE Wallman, A Guilfoyle, S Gordon and KS Courneya [145] | *N* = 558- Australia  Mean age: 59  100% ♀  34% completed college education  Type of cancer:  Breast | Correlational-transversal | **Recall period**:  Typical week-  a) Before diagnosis  b) During treatment  c) After treatment  **Asking for**:  Frequency and duration | **Scoring algorithm**:  Frequency × minutes of mild +  Frequency × minutes of moderate +  Frequency × minutes of strenuous  **Measurement unit**:  Minutes/week  **Scoring algorithm**:  Meeting Australian PA recommendations:  *Active*: ≥ 150 minutes of Moderate PA/week or : ≥ 60 minutes of strenuous PA/week; *insufficiently active*: < 150 minutes of moderate PA/week and < 60 minutes of strenuous PA/week  **Measurement unit**:  Proportion (%) of *active, insufficiently active* individuals | Identify correlates of PA | Relative ranking  Classification | Cancer-related/medical variables  Self-determination theory constructs  Socio-demographic variables |
| DS Mina, MK Connor, SM Alibhai, P Toren, C Guglietti, AG Matthew, J Trachtenberg and P Ritvo [146] | *N* = 26- Canada  Mean age: 72  0% ♀  Type of cancer:  Prostate | Truly experimental | **Recall period**:  Last week  **Asking for**:  Frequency | **Scoring algorithm**:  Frequency of mild × 3 + frequency of moderate × 5 + frequency of strenuous × 9  **Measurement unit**:  LSI (arbitrary units) | Assessment of adherence to exercise program | Relative ranking | N/A |
| R Musanti [147] | *N* = 42- USA  Mean age: 51  100% ♀  Type of cancer:  Breast | Truly experimental | **Recall period**:  Not specified  **Asking for**:  Frequency and duration | **Scoring algorithm**:  Not specified  **Measurement unit**:  METs × hours/week | Served as a control variable in the analyses  Assessment of baseline equivalence of the treatment and comparison groups | Relative ranking | Experimental conditions |
| JM Norris, NJ Moules, G Pelletier and SN Culos-Reed [148] | *N* = 17- Canada  Mean age: 14  53% ♀  Type of cancer:  Leukemia, central nervous system, lymphoma | Correlational-  Transversal | **Recall period**:  Last month  **Asking for**:  Frequency and duration | **Scoring algorithm**:  Frequency of mild + frequency of moderate + frequency of strenuous  Frequency × duration of mild + frequency × duration of moderate + frequency × duration of strenuous  Frequency × duration/60 of mild × 3 + frequency × duration/60 of moderate × 5 + frequency × duration/60 of strenuous × 9  **Measurement unit**:  Number of LTPA cessions (frequency)/week  Minutes/week  METs × hours/week | Examine the association between PA and health-related outcomes  Identify correlates of PA | Relative ranking | Quality of life  Family members |
| E O'Carroll Bantum, CL Albright, KK White, JL Berenberg, G Layi, PL Ritter, D Laurent, K Plant and K Lorig [149] | *N* = 352- USA  Mean age: 51  82% ♀  Mean education level of 16 years  Type of cancer:  Breast, ovarian, uterine, and others | Truly experimental | **Recall period**:  Not specified  **Asking for**:  Frequency and duration | **Scoring algorithm**:  Frequency × duration of moderate + frequency × duration of strenuous  Frequency × duration of mild; moderate; and strenuous  **Measurement unit**:  Minutes/week | Outcome of an intervention | Relative ranking | Experimental conditions |
| EA Olson, SP Mullen, LQ Rogers, KS Courneya, S Verhulst and E McAuley [150] | *N* = 483- USA  Mean age: 63  100% ♀  47% started/completed college/university  Type of cancer:  Breast | Correlational-transversal | **Recall period**:  Last month  **Asking for**:  Frequency and duration | **Scoring algorithm**:  Meeting PA recommendations:  *Active*: ≥ 150 minutes of MVPA/week;  *Insufficiently active*: < 150 minutes of MVPA/week  **Measurement unit**:  Proportion (%) of *active, insufficiently active* individuals | Identify correlates of PA | Classification | Fatigue  Cancer-related/medical variables  Socio-demographic variables  Social cognitive theory constructs |
| LB Packel, AW Prehn, CL Anderson and PL Fisher [151] | *N* = 96- USA  Mean age: 66  ♀: not reported  Mean number of years of education: 15 yr.  Type of cancer:  Colorectal | Correlational-transversal | **Recall period**:  Last month  **Asking for**:  Frequency and duration | **Scoring algorithm**:  Frequency × duration of moderate + frequency × minutes of strenuous × 2  Meeting PA recommendations:  *Active*: ≥ 150 minutes of moderate PA/week or ≥ 75 minutes of strenuous PA/week;  *Insufficiently active*: 1-149 minutes of moderate PA/week and 1-74 minutes of strenuous PA/week;  *No weekly MVPA*: 0 minutes of moderate and strenuous PA/week.  **Measurement unit**:  Minutes/week  Proportion (%) of *active, insufficiently active,* and *no weekly MVPA* individuals | Identify correlates of PA  Assessment of PA patterns during cancer experience | Relative ranking  Classification | Fatigue  Cancer-related/medical variables  Socio-demographic variables  Theory of planned behavior constructs |
| RJ Paxton, LW Jones, PM Rosoff, M Bonner, JL Ater and W Demark-Wahnefried [152] | *N* = 380- USA  Mean age: 18  100% ♀  Type of cancer:  Leukemia, lymphoma, central nervous system | Correlational-  Transversal | **Recall period**:  Not specified  **Asking for**:  Frequency | **Scoring algorithm**:  Frequency of mild × 3 + frequency of moderate × 5 + frequency of strenuous × 9  **Measurement unit**:  LSI (arbitrary units) | Examine the association between PA and health-related outcomes | Relative ranking | Quality of life |
| CJ Peddle, HJ Au and KS Courneya [153] | *N* = 413- Canada  Mean age: 61  46% ♀  52% completed college education  Type of cancer:  Colorectal | Correlational-  retrospective | **Recall period**:  Typical week-  a) Before diagnosis  b) During treatment  Last month-  c) After treatment  **Asking for**:  Frequency and duration | **Scoring algorithm**:  Frequency × duration of moderate + frequency × duration of strenuous  Frequency × duration of moderate; and strenuous  **Measurement unit**:  Minutes/week  **Scoring algorithm**:  Meeting PA recommendations:  *Active*: ≥ 150 minutes of moderate PA/week or ≥ 60 minutes of strenuous PA/week;  *Insufficiently active*: < 150 minutes of moderate PA/week and < 60 minutes of strenuous PA/week  **Measurement unit**:  Proportion (%) of *active, insufficiently active* individuals | Assessment of PA patterns during cancer experience  Examine the association between PA and health-related outcomes | Relative ranking  Classification | Time  Fatigue  Quality of life |
| CJ Peddle, RC Plotnikoff, TC Wild, H-J Au and KS Courneya [154] | *N* = 413- Canada  Mean age: 61  46% ♀  52% completed college education  Type of cancer:  Colorectal | Correlational-  transversal | **Recall period**:  Last month  **Asking for**:  Frequency and duration | **Scoring algorithm**:  Meeting PA recommendations:  *Active*: ≥ 150 minutes of moderate PA/week or ≥ 60 minutes of strenuous PA/week;  *Insufficiently active*: < 150 minutes of moderate PA/week and < 60 minutes of strenuous PA/week;  *No exercise*: 0 minute of MVPA  **Measurement unit**:  Proportion (%) of *active, insufficiently active* and *no exercise* individuals | Identify correlates of PA | Classification | Cancer-related/medical variables  Self-determination theory constructs  Socio-demographic variables |
| C Peeters, A Stewart, R Segal, E Wouterloot, CG Scott and T Aubry [155] | *N* = 66- Canada  Mean age: 63  22% ♀  Type of cancer:  Breast, colorectal, prostate | Correlational-  transversal | **Recall period**:  Last month  **Asking for**:  Frequency and duration | **Scoring algorithm**:  Frequency × duration of moderate + frequency × duration of strenuous  Meeting PA recommendations:  *Active*: ≥ 150 minutes of MVPA;  *Insufficiently active*: < 150 minutes of MVPA/week  **Measurement unit**:  Minutes/week  Proportion (%) of *active* and *insufficiently active* individuals | Describe study sample in terms of LTPA  Investigate exercise programming and counselling preferences | Classification | Exercise programming and counselling preferences |
| HY Perkins, AJ Waters, GP Baum and KM Basen-Engquist [156] | *N* = 20- USA  Mean age: not specified  100% ♀  Type of cancer:  Endometrial | Correlational-  longitudinal | **Recall period**:  Typical week-Last 6 months  **Asking for**:  Frequency and duration | **Scoring algorithm**:  Meeting PA recommendations:  *Active*: ≥ 150 minutes of MVPA/week; *insufficiently active*: < 150 minutes of MVPA/week  **Measurement unit**:  Proportion (%) of *active* and *insufficiently active* individual | Eligibility criterion | Classification | N/A |
| FM Perna, L Craft, KM Freund, G Skrinar, M Stone, L Kachnic, C Youren and TA Battaglia [157] | *N* = 51- Canada  Mean age: 51  100% ♀  Type of cancer:  Breast | Truly experimental | **Recall period**:  Last week  **Asking for**:  Frequency | **Scoring algorithm**:  Frequency of mild × 3 + frequency of moderate × 5 + frequency of strenuous × 9  **Measurement unit**:  LSI (arbitrary units) | Examine the association between PA and health-related outcomes  Outcome of an intervention | Relative ranking | Experimental conditions |
| EJ Philip, EJ Coups, MB Feinstein, BJ Park, DJ Wilson and JS Ostroff [158] | *N* = 175- USA  Mean age: 69  63% ♀  50% completed college/university  Type of cancer:  Lung | Correlational-  transversal | **Recall period**:  Not specified  **Asking for**:  Frequency and duration | **Scoring algorithm**:  Meeting PA recommendations:  *Active*: ≥ 150 minutes of MVPA/week;  *Insufficiently active*: > 0 and < 150 minutes of MVPA/week;  *Sedentary*: 0 minutes of MVPA  **Measurement unit**:  Proportion (%) of *active, insufficiently active* and *sedentary* | Investigate exercise programming and counselling preferences | Classification | Exercise programming and counselling preferences |
| KM Phillips, HS Jim, KA Donovan, MC Pinder-Schenck and PB Jacobsen [159] | *N* = 288- USA  Mean age: 57  65% ♀  40% completed college education  Type of cancer:  Breast, lung, bladder, and other cancers | Correlational-  transversal | **Recall period**:  Last week  **Asking for**:  Frequency and duration | **Scoring algorithm**:  Frequency × duration of mild + frequency × duration of moderate + frequency × duration of strenuous  **Measurement unit**:  Minutes/week | Examine the association between PA and health-related outcomes | Relative ranking | Sleep |
| SM Phillips and E McAuley [160] | *N* = 1527- USA  Mean age: 56  100% ♀  67% completed college education  Type of cancer:  Breast | Correlational-  longitudinal | **Recall period**:  Last week  **Asking for**:  Frequency | **Scoring algorithm**:  Frequency of mild × 3 + frequency of moderate × 5 + frequency of strenuous × 9  **Measurement unit**:  LSI (arbitrary units) | Identify correlates of PA | Relative ranking | Depression  Fatigue  Self-efficacy |
| SM Phillips and E McAuley [161] | *N* = 1527- USA  Mean age: 56  100% ♀  67% completed college education  Type of cancer:  Breast | Correlational-  longitudinal | **Recall period**:  Last week  **Asking for**:  Frequency | **Scoring algorithm**:  Frequency of mild × 3 + frequency of moderate × 5 + frequency of strenuous × 9  **Measurement unit**:  LSI (arbitrary units) | Examine the association between PA and health-related outcomes  Part of a latent PA score with accelerometer data | Relative ranking | Fatigue  Social cognitive theory constructs |
| SM Phillips and E McAuley [162] | *N* = 1527- USA  Mean age: 56  100% ♀  67% completed college education  Type of cancer:  Breast | Correlational-  longitudinal | **Recall period**:  Last week  **Asking for**:  Frequency | **Scoring algorithm**:  Frequency of mild × 3 + frequency of moderate × 5 + frequency of strenuous × 9  **Measurement unit**:  LSI (arbitrary units) | Examine the association between PA and health-related outcomes  Part of a latent PA score with accelerometer data | Relative ranking | Quality of life  Health status  Self-efficacy |
| LQ Rogers, KS Courneya, KT Robbins, J Malone, A Seiz, L Koch and K Rao [163] | *N* = 59- USA  Mean age: 58  17% ♀  Mean years of education: 12  Type of cancer:  Head and neck | Correlational-  transversal | **Recall period**:  Typical month-  a) During the year before cancer diagnosis  b) After diagnosis; i.e., at the time of the survey  **Asking for**:  Frequency and duration | **Scoring algorithm**:  Frequency × duration of mild + frequency × duration of moderate + frequency × duration of strenuous  **Measurement unit**:  Minutes/week | Identify correlates of PA | Relative ranking | Alcohol use  Depression  Role model  Social cognitive theory constructs  Head and neck symptoms |
| LQ Rogers, KS Courneya, KT Robbins, J Malone, A Seiz, L Koch, K Rao and M Nagarkar [164] | *N* = 59- USA  Mean age: 58  17% ♀  Mean years of education: 12  Type of cancer:  Head and neck | Correlational-  retrospective | **Recall period**:  Typical month-  a) During the year before cancer diagnosis  b) After diagnosis; i.e., at the time of the survey  **Asking for**:  Frequency and duration | **Scoring algorithm**:  Frequency × duration of mild + frequency × duration of moderate + frequency × duration of strenuous  **Measurement unit**:  Minutes/week  *Categorical score*-  **Scoring algorithm**:  Meeting PA recommendations:  *Active*: ≥ 150 minutes of moderate LTPA/week or ≥ 60 minutes of strenuous LTPA/week;  *Insufficiently active*: < 150 minutes of moderate /week and < 60 minutes of strenuous LTPA/week  **Measurement unit**:  Proportion (%) of *active* and *insufficiently active* individuals | Examine the association between PA and health-related outcomes  Examine the prevalence of PA | Relative ranking  Classification | Depression  Fatigue  Quality of life |
| LQ Rogers, KS Courneya, KT Robbins, K Rao, J Malone, A Seiz, S Reminger, SJ Markwell and V Burra [165] | *N* = 58- USA  Mean age: 58  36% ♀  Mean years of education: 13  Type of cancer:  Head and neck | Correlational-  transversal | **Recall period**:  Last month  **Asking for**:  Frequency and duration | **Scoring algorithm**:  Frequency × duration of moderate + frequency × duration of strenuous  **Measurement unit**:  Minutes/week | Examine the association between PA and health-related outcomes | Relative ranking | Cognitive function  Fatigue  Sleep |
| LQ Rogers, KS Courneya, S Verhulst, SJ Markwell and E McAuley [166] | *N* = 192- USA  Mean age: 64  100% ♀  Mean years of education: 14  Type of cancer:  Breast | Correlational-  transversal | **Recall period**:  Last month  **Asking for**:  Frequency and duration | **Scoring algorithm**:  Meeting PA recommendations:  *Active*: ≥ 150 minutes of MVPA/week;  *Insufficiently active*: < 150 minutes of MVPA/week; *sedentary*: 0 minute of MVPA  **Measurement unit**:  Proportion (%) of *active, insufficiently active* sedentary individuals | Investigate exercise programming and counselling preferences | Classification | Exercise programming and counselling preferences |
| LQ Rogers, P Hopkins-Price, S Vicari, R Pamenter, KS Courneya, S Markwell, S Verhulst, K Hoelzer, C Naritoku, L Jones, et al. [167] | *N* = 41- USA  Mean age: 53  100% ♀  Mean years of education: 15  Type of cancer:  Breast | Truly experimental | **Recall period**:  Last month  **Asking for**:  Frequency and duration | **Scoring algorithm**:  Frequency × duration of moderate + frequency × duration of strenuous  **Measurement unit**:  Minutes/week | Outcome of an intervention | Relative ranking | Experimental conditions |
| LQ Rogers, J Malone, K Rao, KS Courneya, A Fogleman, A Tippey, SJ Markwell and KT Robbins [168] | *N* = 41- USA  Median age: 65  22% ♀  Years of education: 12 for 33% of the sample  Type of cancer:  Head and neck | Correlation-  transversal | **Recall period**:  Last month  **Asking for**:  Frequency and duration | **Scoring algorithm**:  Frequency × duration of mild + frequency × duration of moderate LTPA + frequency × duration of strenuous  **Measurement unit**:  Minutes/week | Investigate exercise programming and counselling preferences | Relative ranking | Exercise programming and counselling preferences |
| LQ Rogers, SJ Markwell, KS Courneya, E McAuley and S Verhulst [169]  LQ Rogers, SJ Markwell, S Verhulst, E McAuley and KS Courneya [170] | *N* = 476- USA  Mean age: 63  100% ♀  Mean years of education: 13  Type of cancer:  Breast | Correlational-  transversal | **Recall period**:  Last month  **Asking for**:  Frequency and duration | **Scoring algorithm**:  Meeting PA recommendations:  *Active*: ≥ 150 minutes of moderate-to-strenuous LTPA/week;  *Insufficiently active*: < 150 minutes of moderate-to-strenuous LTPA/week; *Sedentary*: 0 minute of moderate-to-strenuous LTPA  **Measurement unit**:  Proportion (%) of *active, insufficiently active sedentary* individuals | Investigate exercise programming and counselling preferences | Relative ranking | Exercise programming and counselling preferences |
| LQ Rogers, E McAuley, KS Courneya and SJ Verhulst [171] | *N* = 192- USA  Mean age: 64  100% ♀  Mean years of education: 14  Type of cancer:  Breast | Correlational-  retrospective | **Recall period**:  Last month-  a) Before diagnosis  b) After diagnosis; i.e., at the time of survey  **Asking for**:  Frequency and duration | **Scoring algorithm**:  Frequency × duration of moderate + frequency × duration of strenuous  **Measurement unit**:  Minutes/week | Identify correlates of PA | Relative ranking | Clinical/medical variables  Fatigue  Social cognitive theory constructs  Socio-demographic variables |
| E Ruden, DA Reardon, AD Coan, JE Herndon Ii, WE Hornsby, M West, DR Fels, A Desjardins, JJ Vredenburgh, E Waner, et al. [172] | *N* = 243- USA  Mean age: 49  32% ♀  Type of cancer:  Brain (malignant glioma) | Correlational-  longitudinal | **Recall period**:  Since treatment  **Asking for**:  Frequency and duration | **Scoring algorithm**:  Frequency × duration/60 of mild LTPA × 3 + frequency × duration/60 of moderate LTPA × 5 + frequency × duration/60 of strenuous LTPA × 9  **Measurement unit**:  METs × hours/week | Examine the association between PA and health-related outcomes | Relative ranking | All-cause mortality |
| D Santa Mina, SM Alibhai, AG Matthew, CL Guglietti, M Pirbaglou, J Trachtenberg and P Ritvo [173] | *N* = 66- Canada  Mean age: 76  0% ♀  Completed high school: 67%  Type of cancer:  Prostate | Truly experimental | **Recall period**:  Last week  **Asking for**:  Frequency and duration | **Scoring algorithm**:  Frequency × duration/60 of mild LTPA × 3 + frequency × duration/60 of moderate LTPA × 5 + frequency × duration/60 of strenuous LTPA × 9  **Measurement unit:**  METs × hours/week | Assessment of baseline equivalence of the treatment and comparison groups  Outcome of an intervention | Relative ranking | Experimental conditions |
| D Santa Mina, CL Guglietti, SMH Alibhai, AG Matthew, R Kalnin, N Ahmad, U Lindner and J Trachtenberg [174] | *N* = 509- Canada  Mean age: 60  0% ♀  Completed college: 40%  Type of cancer:  Prostate | Correlational-  longitudinal | **Recall period**:  Last month  **Asking for**:  Frequency and duration | **Scoring algorithm**:  Meeting PA recommendations:  *Active*: ≥ 150 minutes of moderate LTPA/week or ≥ 75 minutes of strenuous LTPA/week;  *Insufficiently active*: < 150 minutes of moderate LTPA/week and < 75 minutes of strenuous LTPA/week  **Measurement unit**:  Proportion (%) of *active* and *insufficiently active* individuals | Examine the association between PA and health-related outcomes | Classification | Quality of life  Sexual dysfunction  Urinary incontinence |
| D Santa Mina, CL Guglietti, DR de Jesus, S Azargive, AG Matthew, SMH Alibhai, J Trachtenberg, JZ Daskalakis and P Ritvo [175] | *N* = 36- Canada  Mean age: 65  0% ♀  69% completed college/university  Type of cancer:  Prostate | Truly experimental | **Recall period**:  Typical week  **Asking for**:  Frequency and duration | **Scoring algorithm**:  Frequency × duration of mild + frequency × duration of moderate + frequency × duration of strenuous  Meeting PA recommendations:  *Active*: Not specified *Insufficiently active*: Not specified  **Measurement unit**:  Minutes/week  Proportion (%) of *active* and *insufficiently active* individuals | Assessment of baseline equivalence of the treatment and comparison groups | Relative ranking  Classification | Experimental conditions |
| BC Serdà, P Monreal and A Del Valle [176] | *N* = 36- Spain  Mean age: 72  0% ♀  Type of cancer:  Prostate | Pre-experimental | **Recall period**:  Not specified  **Asking for**:  Frequency | **Scoring algorithm**:  Not specified  **Measurement unit**:  Not specified | Outcome of an intervention | Not specified | Experimental conditions |
| CE Short, EL James, C Vandelanotte, KS Courneya, MJ Duncan, A Rebar and RC Plotnikoff [177] | *N* = 330- Australia  Mean age: 56  100% ♀  77% completed college/university  Type of cancer:  Breast | Correlational-  transversal | **Recall period**:  Last month  **Asking for**:  Frequency and duration | **Scoring algorithm**:  Frequency × duration of moderate + frequency × duration of strenuous × 2  Meeting PA recommendations:  *Active*: ≥ 150 minutes of MVPA;  *Insufficiently active*: < 150 minutes of MVPA  **Measurement unit**:  Minutes/week  Proportion (%) of *active* and *insufficiently active* individuals | Identify correlates of PA | Relative ranking  Classification | Clinical/medical variables  Quality of life  Social cognitive theory constructs  Socio-demographic variables |
| AE Speed-Andrews, RE Rhodes, CM Blanchard, SN Culos-Reed, CM Friedenreich, LJ Belanger and KS Courneya [178] | *N* = 600- Canada  Median age: 65  42% ♀  51% completed college education  Type of cancer:  Colorectal | Correlational-  transversal | **Recall period**:  Last month  **Asking for**:  Frequency and duration | **Scoring algorithm**:  Meeting PA recommendations:  *Above guidelines*: ≥ 300 minutes of MVPA/week; *Active*: 150 to 299 minutes of MVPA/week; *Insufficiently active*: 1 to 149 minutes of MVPA/week;  *Completely sedentary*: 0 minute of MVPA/week  **Measurement unit**:  Proportion (%) of *above guidelines*, *active, insufficiently active* and *completely sedentary* individuals | Identify correlates of PA | Classification | Action planning  Clinical/medical variables  Socio-demographic variables  Theory of planned behavior constructs |
| AE Speed-Andrews, C Stevinson, LJ Belanger, JJ Mirus and KS Courneya [179] | *N* = 24- Canada  Median age: 55  100% ♀  78% completed college education  Type of cancer:  Breast | Pre-experimental | **Recall period**:  Last month  **Asking for**:  Frequency and duration | **Scoring algorithm**:  Meeting PA recommendation:  *Active*: ≥ 150 minutes of MVPA or ≥ 75 minutes of strenuous PA;  *Insufficiently active*: < 150 minutes of MVPA and < 75 minutes of strenuous PA  **Measurement unit**:  Proportion (%) of *active* and *insufficiently active* individuals | Test the difference in % meeting PA guidelines between study completers and drop-outs | Classification | Study completers/  drop-outs |
| DP Steensma, KV Heptinstall, VM Johnson, PJ Novotny, JA Sloan, JK Camoriano, J Niblack, JM Bennett and RA Mesa [180] | *N* = 359- USA  Mean age: 64  44% ♀  Type of cancer:  Myelodysplastic syndromes | Correlational-  transversal | **Recall period**:  Last week  **Asking for**:  Frequency | **Scoring algorithm**:  Frequency of mild × 3 + frequency of moderate × 5 + frequency of strenuous × 9  **Measurement unit**:  LSI (arbitrary units) | Examine the association between PA and drug use | Relative ranking | Erythropoiesis-stimulating agents use |
| LE Stephenson, DG Bebb, RA Reimer and SN Culos-Reed [181] | *N* = 63- Canada  Mean age: 60  48% ♀  36% completed college education  Type of cancer:  Colorectal | Correlational-  transversal | **Recall period**:  Last month  **Asking for**:  Frequency and duration | **Scoring algorithm**:  Frequency of mild × duration of mild × 3/60 + frequency of moderate × duration of moderate × 5/60 + frequency of strenuous × duration of strenuous × 9/60  Meeting PA recommendations:  *Active*: ≥ 10 Moderate-to-strenuous METs × hours/week;  *Insufficiently active*: < 10 Moderate-to-strenuous METs × hours/week  **Measurement unit**:  METs × hours/week  Proportion (%) *active* and *insufficiently active* individuals | Examine the prevalence of PA  Examine the association between PA and health-related outcomes | Relative ranking  Classification | Quality of life  Social support |
| C Stevinson, V Capstick, A Schepansky, K Tonkin, JK Vallance, AB Ladha, H Steed, W Faught and KS Courneya [182] | *N* = 359- Canada  Mean age: 60  100% ♀  Type of cancer:  Ovarian | Correlational-  transversal | **Recall period**:  Last month  **Asking for**:  Frequency and duration | **Scoring algorithm**:  Meeting PA recommendations:  *Active*: ≥ 150 minutes of moderate LTPA/week or ≥ 60 minutes of strenuous LTPA/week;  *Insufficiently active*: < 150 minutes of moderate LTPA/week and < 60 minutes of strenuous LTPA/week  **Measurement unit**:  Proportion (%) of *active* and *insufficiently active* individuals | Investigate exercise programming and counselling preferences | Classification | Exercise programming and counselling preferences |
| C Stevinson, W Faught, H Steed, K Tonkin, AB Ladha, JK Vallance, V Capstick, A Schepansky and KS Courneya [183] | *N* = 359- Canada  Mean age: 60  100% ♀  42% has some post-secondary education  Type of cancer:  Ovarian | Correlational-  transversal | **Recall period**:  Last month  **Asking for**:  Frequency and duration | **Scoring algorithm**:  Meeting PA recommendations*:  *Above guidelines*: ≥ 300 minutes of MVPA/week; *Active*: 150 to 299 minutes of MVPA/week; *Insufficiently active*: 1 to 149 minutes of MVPA/week;  *Completely sedentary*: 0 minute of MVPA/week  *Minutes of strenuous LTPA were multiplied by 2  **Measurement unit**:  Proportion (%) of *above guidelines*, *active, insufficiently active* and *completely sedentary* individuals | Examine the prevalence of PA  Examine the association between PA and health-related outcomes | Classification | Quality of life |
| C Stevinson, H Steed, W Faught, K Tonkin, JK Vallance, AB Ladha, A Schepansky, V Capstick and KS Courneya [184] | *N* = 359- Canada  Mean age: 60  100% ♀  42% has some post-secondary education  Type of cancer:  Ovarian | Correlational-  transversal | **Recall period**:  Last month  **Asking for**:  Frequency and duration | **Scoring algorithm**:  Meeting PA recommendations:  *Above guidelines*: ≥ 300 minutes of MVPA/week; *Active*: 150 t0 299 minutes of MVPA/week; *Insufficiently active*: 1 to 149 minutes of MVPA/week;  *Completely sedentary*: 0 minute of MVPA/week  **Measurement unit**:  Proportion (%) of *above guidelines*, *active, insufficiently active* and *completely sedentary* individuals | Examine the association between PA and health-related outcomes | Classification | Anxiety  Depression  Happiness  Fatigue  Peripheral neuropathy  Sleep |
| C Stevinson, K Tonkin, V Capstick, A Schepansky, AB Ladha, JK Vallance, W Faught, H Steed and KS Courneya [185] | *N* = 359- Canada  Mean age: 60  100% ♀  42% has some post-secondary education  Type of cancer:  Ovarian | Correlational-  transversal | **Recall period**:  Last month  **Asking for**:  Frequency and duration | **Scoring algorithm**:  Meeting ACS PA recommendations:  *Above guidelines*: ≥ 300 minutes of MVPA/week; *active*: 150 to 299 minutes of MVPA/week; *insufficiently active*: 1 to 149 minutes of MVPA/week; *completely sedentary*: 0 minute of MVPA/week  **Measurement unit**:  Proportion (%) of *above guidelines*, *active, insufficiently active* and *completely sedentary* individuals | Identify correlates of PA | Classification | Clinical/medical variables  Socio-demographic variables  Theory of planned behavior constructs |
| C Stevinson, A Lydon and Z Amir [186] | *N* = 748- UK  Mean age: 65  68% ♀  39% completed college/university  Type of cancer:  Breast, haematological, prostate, and others | Correlational-  transversal | **Recall period**:  Last month  **Asking for**:  Frequency and duration | **Scoring algorithm**:  Frequency × duration of moderate + frequency × duration of strenuous  Meeting PA recommendations:  *Active*: ≥ 150 minutes of MVPA/week;  *Active but below guidelines*: 1 to 149 minutes of MVPA/week;  *Inactive*: 0 minutes of MVPA/week  **Measurement unit**:  Minutes/week    Proportion (%) of individuals meeting (*Active*), and not meeting PA guidelines (*Active but below guidelines* and *Inactive* individuals) | Identify correlates of PA  Examine the prevalence of PA | Relative ranking  Classification | Clinical/medical variables  Health status  Socio-demographic variables |
| V Tillmann, AS Darlington, C Eiser, NJ Bishop and HA Davies [187] | *N* = 28- UK  Mean age: 11  39% ♀  Type of cancer:  Leukemia | Case-control | **Recall period**:  Typical week  **Asking for**:  Frequency | **Scoring algorithm**:  Frequency of mild × 3 + frequency of moderate × 5 + frequency of strenuous × 9  **Measurement unit**:  LSI (arbitrary units) | Examine the association between PA and health-related outcomes | Relative ranking | Bone mineral density volume |
| L Trinh, RC Plotnikoff, RE Rhodes, S North and KS Courneya [188] | *N* = 703- Canada  Mean age: 65  37% ♀  40% completed college education  Type of cancer:  Kidney | Correlational-  transversal | **Recall period**:  Last month  **Asking for**:  Frequency and duration | **Scoring algorithm**:  Meeting PA recommendations*:  *Above guidelines*: ≥ 300 minutes of MVPA/week; *active*: 150 to 299 minutes of MVPA/week; *Insufficiently active*: 1 to 149 minutes of MVPA/week;  *Completely sedentary*: 0 minute of MVPA/week  *Minutes of strenuous LTPA were multiplied by 2  **Measurement unit**:  Proportion (%) of *above guidelines*, *active, insufficiently active* and *completely sedentary* individuals | Examine the prevalence of PA  Examine the association between PA and health-related outcomes | Classification | Fatigue  Quality of life |
| L Trinh, RC Plotnikoff, RE Rhodes, S North and KS Courneya [189] | *N* = 703- Canada  Mean age: 65  37% ♀  40% completed college education  Type of cancer:  Kidney | Correlational-  transversal | **Recall period**:  Last month  **Asking for**:  Frequency and duration | **Scoring algorithm**:  Meeting PA recommendations*:  *Above guidelines*: ≥ 300 minutes of MVPA/week; *Active*: 150 to 299 minutes of MVPA/week; *insufficiently active*: 1 to 149 minutes of MVPA/week;  *Completely sedentary*: 0 minute of MVPA/week  *Minutes of strenuous LTPA were multiplied by 2  **Measurement unit**:  Proportion (%) of *above guidelines*, *active, insufficiently active* and *completely sedentary* individuals | Identify correlates of PA | Classification | Clinical/medical variables  Socio-demographic variables  Theory of planned behavior constructs |
| L Trinh, RC Plotnikoff, RE Rhodes, S North and KS Courneya [190] | *N* = 703- Canada  Mean age: 65  37% ♀  40% completed college education  Type of cancer:  Kidney | Correlational-  transversal | **Recall period**:  Last month  **Asking for**:  Frequency and duration | **Scoring algorithm**:  Meeting PA recommendations*:  *Above guidelines*: ≥ 300 minutes of MVPA/week; *Active*: 150 to 299 minutes of MVPA/week; *Insufficiently active*: 1 to 149 minutes of MVPA/week;  *Completely sedentary*: 0 minute of MVPA/week  *Minutes of strenuous LTPA were multiplied by 2  **Measurement unit**:  Proportion (%) of *above guidelines*, *active, insufficiently active* and *completely sedentary* individuals | Investigate exercise programming and counselling preferences | Classification | Exercise programming and counselling preferences |
| L Trinh, RC Plotnikoff, RE Rhodes, S North and KS Courneya [191] | *N* = 540- Canada  Mean age: 63  37% ♀  41% completed college education  Type of cancer:  Kidney | Correlational-  transversal | **Recall period**:  Last month  **Asking for**:  Frequency and duration | **Scoring algorithm**:  Meeting PA recommendations*:  *Above guidelines*: ≥ 300 minutes of MVPA/week; *Active*: 150 to 299 minutes of MVPA/week; *Insufficiently active*: 1 to 149 minutes of MVPA/week;  *Completely sedentary*: 0 minute of MVPA/week  *Minutes of strenuous LTPA were multiplied by 2  **Measurement unit**:  Proportion (%) of *above guidelines*, *active, insufficiently active* and *completely sedentary* individuals | Examine the association between PA and health-related outcomes | Classification | Quality of life  Sitting time |
| L Trinh, RC Plotnikoff, RE Rhodes, S North and KS Courneya [192] | *N* = 32- Canada  Mean age: 62  50% ♀  44% completed college/university  Type of cancer:  Kidney | Truly experimental | **Recall period**:  Last month  **Asking for**:  Frequency and duration | **Scoring algorithm**:  Frequency × duration of moderate + frequency × duration of strenuous × 2  Frequency × duration of moderate; and strenuous  Meeting PA recommendations:  *Active*: ≥ 150 minutes of moderate PA/week or ≥ 75 minutes of strenuous PA/week;  *Insufficiently active*: < 150 minutes of moderate PA/week and < 75 minutes of strenuous PA/week  **Measurement unit**:  Minutes/week  Proportion (%) of *active, insufficiently active* individuals | Outcome of an intervention | Relative ranking  Classification | Experimental conditions |
| A Tyrrell, M Keats and CM Blanchard [193] | *N* = 239- Canada  Mean age: 53  100% ♀  Type of cancer:  Cervical, ovarian, uterine, vaginal | Correlational- transversal | **Recall period**:  Last week  **Asking for**:  Frequency and duration | **Scoring algorithm**:  Meeting PA recommendations*:  *Above guidelines*: ≥ 300 minutes of MVPA/week; *Active*: 150 to 299 minutes of MVPA/week; *Insufficiently active*: 1 to 149 minutes of MVPA/week;  *Completely sedentary*: 0 minute of MVPA/week  *Minutes of strenuous LTPA were multiplied by 2  **Measurement unit**:  Proportion (%) of *above guidelines*, *active, insufficiently active* and *completely sedentary* individuals | Investigate exercise programming and counselling preferences | Classification | Exercise programming and counselling preferences |
| M Valenti, G Porzio, F Aielli, L Verna, K Cannita, R Manno, F Masedu, P Marchetti and C Ficorella [194] | *N* = 212- Italy  Mean age: 55  100% ♀  11% completed graduate education  Type of cancer:  Breast | Correlational-  retrospective | **Recall period**:  Typical week-  a) Before diagnosis  b) During treatment  c) After treatment  **Asking for**:  Frequency and duration | **Scoring algorithm**:  Frequency × duration of mild + frequency × duration of moderate + frequency × duration of strenuous  Frequency × duration of mild; moderate; and strenuous  **Measurement unit**:  Minutes/week | Assessment of PA patterns during cancer experience  Examine the association between PA and health-related outcomes | Relative ranking | Quality of life |
| J Vallance, C Lavallee, N Culos-Reed and M Trudeau [195] | *N* = 524- Canada  Mean age: 62  100% ♀  50% complete graduate education  Type of cancer:  Breast | Correlational-  retrospective | **Recall period**:  Last month-  a) Before diagnosis  b) During treatment  c) After treatment  **Asking for**:  Frequency and duration | **Scoring algorithm**:  Frequency × duration of moderate + frequency × duration of strenuous.  **Measurement unit**:  Minutes/week | Investigate exercise programming and counselling preferences | Relative ranking | Exercise programming and counselling preferences |
| J Vallance, RC Plotnikoff, KH Karvinen, JR Mackey and KS Courneya [196] | *N* = 377- Canada  Mean age: 58  100% ♀  30% complete graduate education  Type of cancer:  Breast | Correlational-  longitudinal | **Recall period**:  Last 6 months  **Asking for**:  Frequency and duration | **Scoring algorithm**:  Meeting PA recommendations:  *Active*: ≥ 150 minutes of MVPA/week;  *Insufficiently active*: < 150 minutes of MVPA/week  **Measurement unit**:  Proportion (%) of *active* and *insufficiently active* individuals | Identify correlates of PA | Classification | Clinical/medical variables  Socio-demographic variables  Theory of planned behavior constructs |
| JK Vallance, KS Courneya, RC Plotnikoff, I Dinu and JR Mackey [197] | *N* = 377- Canada  Mean age: 58  100% ♀  30% complete graduate education  Type of cancer:  Breast | Truly experimental | **Recall period**:  Last 6 months  **Asking for**:  Frequency and duration | **Scoring algorithm**:  Frequency × duration of moderate LTPA + frequency × duration of strenuous LTPA  **Measurement unit**: Minutes/week | Outcome of an intervention  Test the difference in MVPA between completers and drop-outs | Relative ranking | Experimental conditions |
| JK Vallance, C Lavallee, NS Culos-Reed and MG Trudeau [198] | *N* = 524- Canada  Mean age: 62  100% ♀  Type of cancer:  Breast | Correlational-  transversal | **Recall period**:  Last months  **Asking for**:  Frequency and duration | **Scoring algorithm**:  Frequency × minutes of moderate + frequency × minutes of strenuous LTPA.  **Measurement unit**:  Minutes/week | Identify correlates of PA | Relative ranking | Self-efficacy  Theory of planned behavior constructs |
| JK Vallance, CM Lavallee, NS Culos-Reed and MG Trudeau [199] | *N* = 524- Canada  Mean age: 62  100% ♀  41% completed graduate education  Type of cancer:  Breast | Correlational-  retrospective | **Recall period**:  Last months-  a) Before diagnosis  b) During treatment  c) After treatment  **Asking for**:  Frequency and duration | **Scoring algorithm**:  Frequency × duration of mild + frequency × duration of moderate + frequency × duration of strenuous;  Frequency × duration of mild; moderate; and strenuous  Meeting PA recommendations:  *Active*: ≥ 30 minutes of moderate LTPA for ≥ 5 days/week or ≥ 20 minutes of moderate LTPA for ≥ 3 days/week;  *Insufficiently active*: not meeting the above guidelines  **Measurement unit**:  Minutes/week  Proportion (%) of *active* and *insufficiently active* individuals | Assessment of PA patterns during cancer experience  Examine the association between PA and health-related outcomes | Relative ranking  Classification | Fatigue  Quality of life  Time |
| JKH Vallance, KS Courneya, LW Jones and T Reiman [200] | *N* = 438- Canada  Mean age: 61  48% ♀  45% completed graduate education  Type of cancer:  Non-Hodgkin’s lymphoma | Correlational-  retrospective | **Recall period**:  Last months-  a) Before diagnosis  b) During treatment  c) After treatment  **Asking for**:  Frequency and duration | **Scoring algorithm**:  Frequency × duration of mild + frequency × duration of moderate + frequency × duration of strenuous;  Frequency × duration of mild; moderate; and strenuous  Meeting PA recommendations:  *Active*: ≥ 150 minutes of MVPA/week;  *Insufficiently active*: < 150 minutes of MVPA/week  **Measurement unit**:  Minutes/week  Proportion (%) of *active* and *insufficiently active* individuals | Assessment of PA patterns during cancer experience  Examine the association between PA and health-related outcomes | Relative ranking  Classification | Fatigue  Quality of life  Time |
| JKH Vallance, KS Courneya, LW Jones and T Reiman [201] | *N* = 438- Canada  Mean age: 61  48% ♀  45% completed graduate education  Type of cancer:  Non-Hodgkin’s lymphoma | Correlational-  transversal | **Recall period**:  Last month  **Asking for**:  Frequency and duration | **Scoring algorithm**:  Frequency of mild; moderate; and strenuous  Frequency × duration of mild; moderate; and strenuous  Meeting PA recommendations:  *Active*: ≥ 150 minutes of MVPA/week;  *Insufficiently active*: < 150 minutes of MVPA/week  **Measurement unit**:  Number of LTPA cessions (frequency)/week  Minutes/week  Proportion (%) of *active* and *insufficiently active* individuals | Investigate exercise programming and counselling preferences | Relative ranking  Classification | Exercise programming and counselling preferences |
| JKH Vallance, KS Courneya, RC Plotnikoff and JR Mackey [202] | *N* = 377- Canada  Mean age: 58  100% ♀  30% complete graduate education  Type of cancer:  Breast | Truly experimental | **Recall period**:  Typical week  **Asking for**:  Frequency and duration | **Scoring algorithm**:  Frequency × duration of moderate + frequency × duration of strenuous  **Measurement unit**:  Minutes/week | Outcome of an intervention  Identify correlates (mediators) of PA | Relative ranking | Experimental conditions    Theory of planned behavior constructs |
| JKH Vallance, KS Courneya, RC Plotnikoff, Y Yasui and JR Mackey [203] | *N* = 377- Canada  Mean age: 58  100% ♀  30% complete graduate education  Type of cancer:  Breast | Truly experimental | **Recall period**:  Last month  **Asking for**:  Frequency and duration | **Scoring algorithm**:  Frequency × duration of moderate + frequency × duration of strenuous  **Measurement unit**:  Minutes/week | Assessment of baseline equivalence of the treatment and comparison groups  Outcome of an intervention | Relative ranking | Experimental conditions |
| CG Valle, DF Tate, DK Mayer, M Allicock and J Cai [204] | *N* = 86- USA  Mean age: 55  100% ♀  36% complete graduate education  Type of cancer:  All types of cancer (excluding nonmelanoma skin cancer) | Truly experimental | **Recall period**:  Last week  **Asking for**:  Frequency and duration | **Scoring algorithm**:  Frequency × duration of moderate + frequency × duration of strenuous  Frequency × duration of mild + frequency × duration of moderate + frequency × duration of strenuous  **Measurement unit**:  Minutes/week | Outcome of an intervention | Relative ranking | Experimental conditions |
| V von Gruenigen, H Frasure, MB Kavanagh, J Janata, S Waggoner, P Rose, E Lerner and KS Courneya [205] | *N* = 75- USA  Mean age: 58  100% ♀  40% complete graduate education  Type of cancer:  Endometrial | Truly experimental | **Recall period**:  Typical week  **Asking for**:  Frequency and duration | **Scoring algorithm**:  Frequency of mild × 3 + frequency of moderate × 5 + frequency of strenuous × 9  Frequency × duration of moderate + frequency × duration of strenuous × 2  **Measurement unit**:  LSI (arbitrary units)  Minutes/week | Outcome of an intervention | Relative ranking | Experimental conditions |
| VE von Gruenigen, KS Courneya, HE Gibbons, MB Kavanagh, SE Waggoner and E Lerner [206] | *N* = 45- USA  Mean age: 55  100% ♀  36% complete graduate education  Type of cancer:  Endometrial | Truly experimental | **Recall period**:  Typical week  **Asking for**:  Frequency | **Scoring algorithm**:  Frequency of mild × 3 + frequency of moderate × 5 + frequency of strenuous × 9  **Measurement unit**:  LSI (arbitrary units) | Outcome of an intervention | Relative ranking | Experimental conditions |
| VE von Gruenigen, HE Frasure, MB Kavanagh, E Lerner, SE Waggoner and KS Courneya [207] | *N* = 27- USA  Mean age: 60  100% ♀  33% complete graduate education  Type of cancer:  Ovarian | Pre-experimental  (prospective) | **Recall period**:  Last month-  a) Baseline  b) During treatment  c) After treatment  **Asking for**:  Frequency and duration | **Scoring algorithm**:  Frequency × duration of moderate + frequency × duration of strenuous × 2  **Measurement unit**:  Minutes/week | Examine the association between PA and health-related outcomes  Assessment of PA patterns during cancer experience  Outcome of an intervention | Relative ranking | Experimental conditions  Quality of life  Time |
| VE von Gruenigen, SE Waggoner, HE Frasure, MB Kavanagh, JW Janata, PG Rose, KS Courneya and E Lerner [208] | *N* = 120- USA  Mean age: 57  100% ♀  38% complete graduate education  Type of cancer:  Endometrial | Correlational-  transversal | **Recall period**:  Typical week  **Asking for**:  Frequency and duration | **Scoring algorithm**:  Meeting PA recommendations:  *Active*: ≥ 150 minutes of MVPA/week;  *Insufficiently active*: < 150 minutes of MVPA/week    **Measurement unit**:  Proportion (%) of *active* and *insufficiently active* individuals | Examine the association between PA and health-related outcomes | Classification | Quality of life |
| Y-J Wang, M Boehmke, Y-WB Wu, SS Dickerson and N Fisher [209] | *N* = 72- Taiwan  Mean age: 50  100% ♀  46% complete graduate education  Type of cancer:  Breast | Truly experimental | **Recall period**:  Last week  **Asking for**:  Frequency | **Scoring algorithm**:  Frequency of mild × 3 + frequency of moderate × 5 + frequency of strenuous × 9  **Measurement unit**:  LSI (arbitrary units) | Outcome of an intervention | Relative ranking | Experimental conditions |
| M Wright, A Bryans, K Gray, L Skinner and A Verhoeve [210] | *N* = 48- Canada  Mean age: 16  40% ♀  Type of cancer:  Leukemia, CNS tumor, lymphoma | Case-control | **Recall period**:  Typical week  **Asking for**:  Frequency | **Scoring algorithm**:  Frequency of mild × 3 + frequency of moderate × 5 + frequency of strenuous × 9  **Measurement unit**:  LSI (arbitrary units) | Identify correlates of PA  Examine the association between PA and health-related outcomes | Relative ranking | Barriers  Social support  Socio-demographic variables  The case and control groups |
| C Wrosch and CM Sabiston [211] | *N* = 177- Canada  Mean age: 55  100% ♀  Type of cancer:  Breast | Correlational-  longitudinal | **Recall period**:  Typical week  **Asking for**:  Frequency and duration | **Scoring algorithm**:  Frequency × duration of moderate + frequency × duration of strenuous  **Measurement unit**:  Minutes/week | Identify correlates of PA  Examine the association between PA and health-related outcomes | Relative ranking | Goal adjustment constructs  Emotional well-being  Physical health |
| A Wurz, C Chamorro-Vina, GMT Guilcher, F Schulte and SN Culos-Reed [212] | *N* = 8- Canada  Mean age: 12  50% ♀  Type of cancer:  Central nervous system tumors, leukemia, lymphoma, and osteosarcoma | Pre-experimental | **Recall period**:  Not specified  **Asking for**:  Frequency and duration | **Scoring algorithm**:  Frequency × duration of mild × 3 + frequency × duration of moderate × 5 + frequency × duration of strenuous × 9  Frequency of mild; moderate; and strenuous  Frequency × duration of mild; moderate; and strenuous  Frequency × duration of mild × 3;  Frequency × duration of moderate × 5;  Frequency × duration of strenuous × 9  **Measurement unit**:  Frequency/week  Minutes/week  METs hour/week | Outcome of an intervention | Relative ranking | Experimental condition/time |
| ANR Zahavich, JA Robinson, D Paskevich and SN Culos-Reed [213] | *N* = 15- Canada  Mean age: 55  0% ♀  80% completed college education  Type of cancer:  Prostate | Pre-experimental | **Recall period**:  Not specified  **Asking for**:  Frequency and duration | **Scoring algorithm**:  Frequency of mild + frequency of moderate + frequency of strenuous  Frequency of mild × 3 + frequency of moderate × 5 + frequency of strenuous × 9  Frequency × duration of mild + frequency × duration of moderate + frequency × duration of strenuous  **Measurement unit**:  Number of LTPA cessions (frequency)/week  LSI (arbitrary units)  Minutes/week | Outcome of an intervention | Relative ranking | Experimental conditions |

*Note*.^1^Only variables linked with LTPA measured with the GSLTPAQ were identified. ACS: American Cancer Society. ACSM: American College of Sports and Medicine. BMI: body mass index. CNS: Central nervous system. LSI: leisure score index. PSA: prostate-specific antigen. MET: Metabolic equivalent of task. RCT: randomized controlled trial. SCT: social cognitive theory. TPB: theory of planned behavior. Information between brackets “[ ]” were obtained from another studies.

**References**

*Article excluded from the systematic review

1. Alibhai SMH, O'Neill S, Fisher-Schlombs K, Breunis H, Brandwein JM, Timilshina N, Tomlinson GA, Klepin HD, Culos-Reed SN: **A clinical trial of supervised exercise for adult inpatients with acute myeloid leukemia (AML) undergoing induction chemotherapy**. *Leuk Res* 2012, **36**:1255-1261.

2. Alibhai SMH, O'Neill S, Fisher-Schlombs K, Breunis H, Timilshina N, Brandwein JM, Minden MD, Tomlinson GA, Culos-Reed SN: **A pilot phase II RCT of a home-based exercise intervention for survivors of AML**. *Support Care Cancer* 2014, **22**:881-889.

3. Andrykowski MA, Beacham AO, Jacobsen PB: **Prospective, longitudinal study of leisure-time exercise in women with early-stage breast cancer**. *Cancer Epidemiol Biomarkers Prev* 2007, **16**:430-438.

4. Badr H, Chandra J, Paxton RJ, Ater JL, Urbauer D, Cruz CS, Demark-Wahnefried W: **Health-related quality of life, lifestyle behaviors, and intervention preferences of survivors of childhood cancer**. *J Cancer Surviv* 2013, **7**:523-534.

5. Badr H, Paxton RJ, Ater JL, Urbauer D, Demark-Wahnefried W: **Health behaviors and weight status of childhood cancer survivors and their parents: Similarities and opportunities for joint interventions**. *J Am Diet Assoc* 2011, **111**:1917-1923.

6. Baldwin MK, Courneya KS: **Exercise and self-esteem in breast cancer survivors: An application of the exercise and self-esteem model**. *J Sport Exerc Psychol* 1997, **19**:347-358.

7. Bélanger LJ, Plotnikoff RC, Clark A, Courneya KS: **Physical activity and health-related quality of life in young adult cancer survivors: a Canadian provincial survey**. *J Cancer Surviv* 2011, **5**:44-53.

8. Bélanger LJ, Plotnikoff RC, Clark AM, Courneya KS: **Determinants of physical activity in young adult cancer survivors**. *Am J Health Behav* 2012, **36**:483-494.

9. Belanger LJ, Mummery WK, Clark AM, Courneya KS: **Effects of Targeted Print Materials on Physical Activity and Quality of Life in Young Adult Cancer Survivors During and After Treatment: An Exploratory Randomized Controlled Trial**. *J Adolesc Young Adult Oncol* 2014, **3**:83-91.

10. Bellury L, Ellington L, Beck SL, Pett MA, Clark J, Stein K: **Older breast cancer survivors: can interaction analyses identify vulnerable subgroups? A report from the American Cancer Society Studies of Cancer Survivors**. *Oncol Nurs Forum* 2013, **40**:325-336.

11. Blanchard CM, Courneya KS, Rodgers WM, Murnaghan DM: **Determinants of exercise intention and behavior in survivors of breast and prostate cancer: An application of the theory of planned behavior**. *Cancer Nurs* 2002, **25**:88-95.

12. Blanchard CM, Courneya KS, Stein K: **Cancer survivors' adherence to lifestyle behavior recommendations and associations with health-related quality of life: Results from the American Cancer Society's SCS-II**. *J Clin Oncol* 2008, **26**:2198-2204.

13. Blanchard CM, Stein K, Courneya KS: **Body mass index, physical activity, and health-related quality of life in cancer survivors**. *Med Sci Sports Exerc* 2010, **42**:665-671.

14. Blaney JM, Lowe-Strong A, Rankin-Watt J, Campbell A, Gracey JH: **Cancer survivors' exercise barriers, facilitators and preferences in the context of fatigue, quality of life and physical activity participation: a questionnaire-survey**. *Psycho-Oncology* 2013, **22**:186-194.

15. Bolam K, Beck B, Adlard K, Skinner T, Cormie P, Galvão D, Spry N, Newton R, Taaffe D: **The relationship between BPAQ-derived physical activity and bone density of middle-aged and older men**. *Osteoporosis Int* 2014, **25**:2663-2668.

16. Bourke L, Doll H, Crank H, Daley A, Rosario D, Saxton JM: **Lifestyle intervention in men with advanced prostate cancer receiving androgen suppression therapy: a feasibility study**. *Cancer Epidemiol Biomarkers Prev* 2011, **20**:647-657.

17. Bourke L, Thompson G, Gibson DJ, Daley A, Crank H, Adam I, Shorthouse A, Saxton J: **Pragmatic lifestyle intervention in patients recovering from colon cancer: a randomized controlled pilot study**. *Arch Phys Med Rehabil* 2011, **92**:749-755.

18. Bourke L, Gilbert S, Hooper R, Steed LA, Joshi M, Catto JWF, Saxton JM, Rosario DJ: **Lifestyle Changes for Improving Disease-specific Quality of Life in Sedentary Men on Long-term Androgen-Deprivation Therapy for Advanced Prostate Cancer: A Randomised Controlled Trial**. *Eur Urol* 2014, **65**:865-872.

19. Broderick JM, Guinan E, Kennedy MJ, Hollywood D, Courneya KS, Culos-Reed SN, Bennett K, DM OD, Hussey J: **Feasibility and efficacy of a supervised exercise intervention in de-conditioned cancer survivors during the early survivorship phase: the PEACH trial**. *J Cancer Surviv* 2013, **7**:551-562.

20. Brunet J, Sabiston CM: **Self-presentation and physical activity in breast cancer survivors: The moderating effect of social cognitive constructs**. *J Sport Exerc Psychol* 2011, **33**:759-778.

21. Brunet J, Amireault S, Chaiton M, Sabiston CM: **Identification and prediction of physical activity trajectories in women treated for breast cancer**. *Ann Epidemiol* 2014, **24**:837-842.

22. Brunet J, Love C, Ramphal R, Sabiston CM: **Stress and physical activity in young adults treated for cancer: The moderating role of social support**. *Support Care Cancer* 2014, **22**:689-695.

23. Brunet J, Sabiston CM, Gaudreau P: **A prospective investigation of the relationships between self-presentation processes and physical activity in women treated for breast cancer**. *Health Psychol* 2014, **33**:205-213.

24. Burton AJ, Martin RM, Donovan JL, Lane JA, Davis M, Hamdy FC, Neal DE, Tilling K: **Associations of lifestyle factors and anthropometric measures with repeat PSA Levels during active surveillance/monitoring**. *Cancer Epidemiol Biomarkers Prev* 2012, **21**:1877-1885.

25. Casla S, Hojman P, Cubedo R, Calvo I, Sampedro J, Barakat R: **Integrative exercise and lifestyle intervention increases leisure-time activity in breast cancer patients**. *Integr Cancer Ther* 2014, **13**:493-501.

26. Clark MM, Vickers KS, Hathaway JC, Smith M, Looker SA, Petersen LR, Pinto BM, Rummans TA, Loprinzi CL: **Physical activity in patients with advanced-stage cancer actively receiving chemotherapy**. *J Support Oncol* 2007, **5**:487-493.

27. Cooley ME, Finn KT, Wang Q, Roper K, Morones S, Shi L, Litrownik D, Marcoux JP, Zaner K, Hayman LL: **Health behaviors, readiness to change, and interest in health promotion programs among smokers with lung cancer and their family members: A pilot study**. *Cancer Nurs* 2013, **36**:145-154.

28. Cormie P, Newton RU, Spry N, Joseph D, Taaffe DR, Galvao DA: **Safety and efficacy of resistance exercise in prostate cancer patients with bone metastases**. *Prostate Cancer Prostatic Dis* 2013, **16**:328-335.

29. Cormie P, Spry N, Jasas K, Johansson M, Yusoff IF, Newton RU, Galvão DA: **Exercise as medicine in the management of pancreatic cancer: A case study**. *Med Sci Sports Exerc* 2014, **46**:664-670.

30. Coups EJ, Park BJ, Feinstein MB, Steingart RM, Egleston BL, Wilson DJ, Ostroff JS: **Physical activity among lung cancer survivors: Changes across the cancer trajectory and associations with quality of life**. *Cancer Epidemiol Biomarkers Prev* 2009, **18**:664-672.

31. Coups EJ, Park BJ, Feinstein MB, Steingart RM, Egleston BL, Wilson DJ, Ostroff JS: **Correlates of physical activity among lung cancer survivors**. *Psycho-Oncology* 2009, **18**:395-404.

32. Courneya KS, Blanchard CM, Laing DM: **Exercise adherence in breast cancer survivors training for a dragon boat race competition: A preliminary investigation**. *Psycho-Oncology* 2001, **10**:444-452.

33. Courneya KS, Friedenreich CM: **Determinants of exercise during colorectal cancer treatment: an application of the theory of planned behavior**. *Oncol Nurs Forum* 1997, **24**:1715-1723.

34. Courneya KS, Friedenreich CM: **Relationship between exercise during treatment and current quality of life among survivors of breast cancer**. *J Psychosoc Oncol* 1997, **15**:35-57.

35. Courneya KS, Friedenreich CM: **Relationship between exercise pattern across the cancer experience and current quality of life in colorectal cancer survivors**. *J Altern Complem Med* 1997, **3**:215-226.

36. Courneya KS, Friedenreich CM: **Utility of the theory of planned behavior for understanding exercise during breast cancer treatment**. *Psycho-Oncology* 1999, **8**:112-122.

37. Courneya KS, Friedenreich CM, Arthur K, Bobick TM: **Physical exercise and quality of life in postsurgical colorectal cancer patients**. *Psychol Health Med* 1999, **4**:181-187.

38. Courneya KS, Friedenreich CM, Arthur K, Bobick TM: **Understanding exercise motivation in colorectal cancer patients: A prospective study using the theory of planned behavior**. *Rehabil Psychol* 1999, **44**:68-84.

39. Courneya KS, Friedenreich CM, Quinney HA, Fields ALA, Jones LW, Fairey AS: **A randomized trial of exercise and quality of life in colorectal cancer survivors**. *Eur J Cancer Care* 2003, **12**:347-357.

40. Courneya KS, Friedenreich CM, Quinney HA, Fields ALA, Jones LW, Fairey AS: **Predictors of adherence and contamination in a randomized trial of exercise in colorectal cancer survivors**. *Psycho-Oncology* 2004, **13**:857-866.

41. Courneya KS, Friedenreich CM, Quinney HA, Fields ALA, Jones LW, Vallance JKH, Fairey AS: **A longitudinal study of exercise barriers in colorectal cancer survivors participating in a randomized controlled trial**. *Ann Behav Med* 2005, **29**:147-153.

42. Courneya KS, Friedenreich CM, Reid RD, Gelmon K, Mackey JR, Ladha AB, Proulx C, Vallance JK, Segal RJ: **Predictors of follow-up exercise behavior 6 months after a randomized trial of exercise training during breast cancer chemotherapy**. *Breast Cancer Res Treat* 2009, **114**:179-187.

43. Courneya KS, Friedenreich CM, Sela RA, Quinney HA, Rhodes RE: **Correlates of adherence and contamination in a randomized controlled trial of exercise in cancer survivors: An application of the theory of planned behavior and the five factor model of personality**. *Ann Behav Med* 2002, **24**:257-268.

44. Courneya KS, Friedenreich CM, Sela RA, Quinney HA, Rhodes RE, Handman M: **The group psychotherapy and home-based physical exercise (group-hope) trial in cancer survivors: Physical fitness and quality of life outcomes**. *Psycho-Oncology* 2003, **12**:357-374.

45. Courneya KS, Friedenreich CM, Sela RA, Quinney HA, Rhodes RE, Jones LW: **Exercise motivation and adherence in cancer survivors after participation in a randomized controlled trial: An attribution theory perspective**. *Int J Behav Med* 2004, **11**:8-17.

46. Courneya KS, Jones LW, Peddle CJ, Sellar CM, Reiman T, Joy AA, Chua N, Tkachuk L, Mackey JR: **Effects of aerobic exercise training in anemic cancer patients receiving darbepoetin alfa: A randomized controlled trial**. *Oncologist* 2008, **13**:1012-1020.

47. Courneya KS, Karvinen KH, Campbell KL, Pearcey RG, Dundas G, Capstick V, Tonkin KS: **Associations among exercise, body weight, and quality of life in a population-based sample of endometrial cancer survivors**. *Gynecol Oncol* 2005, **97**:422-430.

48. Courneya KS, Keats MR, Turner AR: **Physical exercise and quality of life in cancer patients following high dose chemotherapy and autologous bone marrow transplantation**. *Psycho-Oncology* 2000, **9**:127-136.

49. Courneya KS, Keats MR, Turner AR: **Social cognitive determinants of hospital-based exercise in cancer patients following high-dose chemotherapy and bone marrow transplantation**. *Int J Behav Med* 2000, **7**:189-203.

50. Courneya KS, Mackey JR, Bell GJ, Jones LW, Field CJ, Fairey AS: **Randomized controlled trial of exercise training in postmenopausal breast cancer survivors: Cardiopulmonary and quality of life outcomes**. *J Clin Oncol* 2003, **21**:1660-1668.

51. Courneya KS, McKenzie DC, Reid RD, Mackey JR, Gelmon K, Friedenreich CM, Ladha AB, Proulx C, Lane K, Vallance JK *et al*: **Barriers to supervised exercise training in a randomized controlled trial of breast cancer patients receiving chemotherapy**. *Ann Behav Med* 2008, **35**:116-122.

52. Courneya KS, Reid RD, Friedenreich CM, Gelmon K, Proulx C, Vallance JK, McKenzie DC, Segal RJ: **Understanding breast cancer patients' preference for two types of exercise training during chemotherapy in an unblinded randomized controlled trial**. *Int J Behav Nutr Phys Act* 2008, **5**.

53. Courneya KS, Segal RJ, Gelmon K, Reid RD, Mackey JR, Friedenreich CM, Proulx C, Lane K, Ladha AB, Vallance JK *et al*: **Six-month follow-up of patient-rated outcomes in a randomized controlled trial of exercise training during breast cancer chemotherapy**. *Cancer Epidemiol Biomarkers Prev* 2007, **16**:2572-2578.

54. Courneya KS, Segal RJ, Gelmon K, Reid RD, Mackey JR, Friedenreich CM, Proulx C, Lane K, Ladha AB, Vallance JK *et al*: **Predictors of supervised exercise adherence during breast cancer chemotherapy**. *Med Sci Sports Exerc* 2008, **40**:1180-1187.

55. Courneya KS, Segal RJ, Mackey JR, Gelmon K, Reid RD, Friedenreich CM, Ladha AB, Proulx C, Vallance JKH, Lane K *et al*: **Effects of aerobic and resistance exercise in breast cancer patients receiving adjuvant chemotherapy: A multicenter randomized controlled trial**. *J Clin Oncol* 2007, **25**:4396-4404.

56. Courneya KS, Segal RJ, Reid RD, Jones LW, Malone SC, Venner PM, Parliament MB, Scott CG, Quinney HA, Wells GA: **Three independent factors predicted adherence in a randomized controlled trial of resistance exercise training among prostate cancer survivors**. *J Clin Epidemiol* 2004, **57**:571-579.

57. Courneya KS, Sellar CM, Stevinson C, McNeely ML, Peddle CJ, Friedenreich CM, Tankel K, Basi S, Chua N, Mazurek A *et al*: **Randomized controlled trial of the effects of aerobic exercise on physical functioning and quality of life in lymphoma patients**. *J Clin Oncol* 2009, **27**:4605-4612.

58. Courneya KS, Sellar CM, Trinh L, Forbes CC, Stevinson C, McNeely ML, Peddle-McIntyre CJ, Friedenreich CM, Reiman T: **A randomized trial of aerobic exercise and sleep quality in lymphoma patients receiving chemotherapy or no treatments**. *Cancer Epidemiol Biomarkers Prev* 2012, **21**:887-894.

59. Courneya KS, Stevinson C, McNeely ML, Sellar CM, Friedenreich CM, Peddle-Mcintyre CJ, Chua N, Reiman T: **Effects of supervised exercise on motivational outcomes and longer-term behavior**. *Med Sci Sports Exerc* 2012, **44**:542-549.

60. Courneya KS, Stevinson C, McNeely ML, Sellar CM, Friedenreich CM, Peddle-McIntyre CJ, Chua N, Reiman T: **Predictors of follow-up exercise behavior 6 months after a randomized trial of supervised exercise training in lymphoma patients**. *Psycho-Oncology* 2012, **21**:1124-1131.

61. Courneya KS, Stevinson C, McNeely ML, Sellar CM, Peddle CJ, Friedenreich CM, Mazurek A, Chua N, Tankel K, Basi S *et al*: **Predictors of adherence to supervised exercise in lymphoma patients participating in a randomized controlled trial**. *Ann Behav Med* 2010, **40**:30-39.

62. Courneya KS, Vallance JKH, Jones LW, Reiman T: **Correlates of exercise intentions in non-Hodgkin's lymphoma survivors: An application of the theory of planned behavior**. *J Sport Exerc Psychol* 2005, **27**:335-349.

63. Craike M, Hose K, Livingston PM: **Physical activity participation and barriers for people with multiple myeloma**. *Support Care Cancer* 2013, **21**:927-934.

64. Craike MJ, Hose K, Courneya KS, Harrison SJ, Livingston PM: **Perceived benefits and barriers to exercise for recently treated patients with multiple myeloma: A qualitative study**. *BMC Cancer* 2013, **13:319**.

65. Crosswell AD, Lockwood KG, Ganz PA, Bower JE: **Low heart rate variability and cancer-related fatigue in breast cancer survivors**. *Psychoneuroendocrinology* 2014, **45**:58-66.

66. Crowgey T, Peters KB, Hornsby WE, Lane A, McSherry F, Herndon JE, 2nd, West MJ, Williams CL, Jones LW: **Relationship between exercise behavior, cardiorespiratory fitness, and cognitive function in early breast cancer patients treated with doxorubicin-containing chemotherapy: a pilot study**. *Appl Physiol Nutr Metab* 2014, **39**:724-729.

67. Culos-Reed SN, Carlson LE, Daroux LM, Hately-Aldous S: **A pilot study of yoga for breast cancer survivors: Physical and psychological benefits**. *Psycho-Oncology* 2006, **15**:891-897.

68. Culos-Reed SN, Robinson JL, Lau H, O'Connor K, Keats MR: **Benefits of a physical activity intervention for men with prostate cancer**. *J Sport Exerc Psychol* 2007, **29**:118-127.

69. Culos-Reed SN, Robinson JW, Lau H, Stephenson L, Keats M, Norris S, Kline G, Faris P: **Physical activity for men receiving androgen deprivation therapy for prostate cancer: benefits from a 16-week intervention**. *Support Care Cancer* 2010, **18**:591-599.

70. Culos-Reed SN, Shields C, Brawley LR: **Breast cancer survivors involved in vigorous team physical activity: Psychosocial correlates of maintenance participation**. *Psycho-Oncology* 2005, **14**:594-605.

71. Demark-Wahnefried W, Werner C, Clipp EC, Guill AB, Bonner M, Jones LW, Rosoff PM: **Survivors of childhood cancer and their guardians**. *Cancer* 2005, **103**:2171-2180.

72. Demark-Wahnefried W, Jones LW, Snyder DC, Sloane RJ, Kimmick GG, Hughes DC, Badr HJ, Miller PE, Burke LE, Lipkus IM: **Daughters and Mothers Against Breast Cancer (DAMES): Main Outcomes of a Randomized Controlled Trial of Weight Loss in Overweight Mothers With Breast Cancer and Their Overweight Daughters**. *Cancer* 2014, **120**:2522-2534.

73. Donovan KA, Small BJ, Andrykowski MA, Munster P, Jacobsen PB: **Utility of a cognitive-behavioral model to predict fatigue following breast cancer treatment**. *Health Psychol* 2007, **26**:464-472.

74. Fairey AS, Courneya KS, Field CJ, Bell GJ, Jones LW, Mackey JR: **Effects of exercise training on fasting insulin, insulin resistance, insulin-like growth factors, and insulin-like growth factor binding proteins in postmenopausal breast cancer survivors: A randomized controlled trial**. *Cancer Epidemiol Biomarkers Prev* 2003, **12**:721-727.

75. Fairey AS, Courneya KS, Field CJ, Bell GJ, Jones LW, Mackey JR: **Randomized controlled trial of exercise and blood immune function in postmenopausal breast cancer survivors**. *J Appl Physiol* 2005, **98**:1534-1540.

76. Faul LA, Jim HS, Minton S, Fishman M, Tanvetyanon T, Jacobsen PB: **Relationship of exercise to quality of life in cancer patients beginning chemotherapy**. *J Pain Symptom Manage* 2011, **41**:859-869.

77. Feinstein MB, Krebs P, Coups EJ, Park BJ, Steingart RM, Burkhalter J, Logue A, Ostroff JS: **Current dyspnea among long-term survivors of early-stage non-small cell lung cancer**. *J Thorac Oncol* 2010, **5**:1221-1226.

78. Forbes CC, Blanchard CM, Mummery WK, Courneya KS: **A comparison of physical activity correlates across breast, prostate and colorectal cancer survivors in Nova Scotia, Canada**. *Support Care Cancer* 2014, **22**:891-903.

79. Galvão DA, Spry N, Denham J, Taaffe DR, Cormie P, Joseph D, Lamb DS, Chambers SK, Newton RU: **A multicentre year-long randomised controlled trial of exercise training targeting physical functioning in men with prostate cancer previously treated with androgen suppression and radiation from TROG 03.04 radar**. *Eur Urol* 2014, **65**:856-864.

80. Gilbert SE, Tew GA, Bourke L, Winter EM, Rosario DJ: **Assessment of endothelial dysfunction by flow-mediated dilatation in men on long-term androgen deprivation therapy for prostate cancer**. *Exp Physiol* 2013, **98**:1401-1410.

81. Gilliam MB, Madan-Swain A, Whelan K, Tucker DC, Demark-Wahnefried W, Schwebel DC: **Social, demographic, and medical influences on physical activity in child and adolescent cancer survivors**. *J Pediatr Psychol* 2012, **37**:198-208.

82. Gilliam MB, Madan-Swain A, Whelan K, Tucker DC, Demark-Wahnefried W, Schwebel DC: **Cognitive influences as mediators of family and peer support for pediatric cancer survivors' physical activity**. *Psycho-Oncology* 2013, **22**:1361-1368.

83. Gjerset GM, Fosså SD, Courneya KS, Skovlund E, Jacobsen AB, Thorsen L: **Interest and preferences for exercise counselling and programming among Norwegian cancer survivors**. *Eur J Cancer Care* 2011, **20**:96-105.

84. Gjerset GM, Fosså SD, Courneya KS, Skovlund E, Thorsen L: **Exercise behavior in cancer survivors and associated factors**. *J Cancer Surviv* 2011, **5**:35-43.

85. Gjerset GM, Fosså SD, Dahl AA, Loge JH, Ensby T, Thorsen L: **Effects of a 1-week inpatient course including information, physical activity, and group sessions for prostate cancer patients**. *J Cancer Educ* 2011, **26**:754-760.

86. Grimmett C, Bridgewater J, Steptoe A, Wardle J: **Lifestyle and quality of life in colorectal cancer survivors**. *Qual Life Res* 2011, **20**:1237-1245.

87. Grossman P, Deuring G, Garland SN, Campbell TS, Carlson LE: **Patterns of objective physical functioning and perception of mood and fatigue in posttreatment breast cancer patients and healthy controls: an ambulatory psychophysiological investigation**. *Psychosom Med* 2008, **70**:819-828.

88. Guinan E, Hussey J, Broderick JM, Lithander FE, O'Donnell D, Kennedy MJ, Connolly EM: **The effect of aerobic exercise on metabolic and inflammatory markers in breast cancer survivors - A pilot study**. *Support Care Cancer* 2013, **21**:1983-1992.

89. Harrington JM, Schwenke DC, Epstein DR: **Exercise Preferences Among Men With Prostate Cancer Receiving Androgen-Deprivation Therapy**. *Oncol Nurs Forum* 2013, **40**:E358-E367.

90. Hawkes AL, Chambers SK, Pakenham KI, Patrao TA, Baade PD, Lynch BM, Aitken JF, Meng X, Courneya KS: **Effects of a telephone-delivered multiple health behavior change intervention (CanChange) on health and behavioral outcomes in survivors of colorectal cancer: A randomized controlled trial**. *J Clin Oncol* 2013, **31**:2313-2321.

91. Hocking MC, Schwartz LA, Hobbie WL, Derosa BW, Ittenbach RF, Mao JJ, Ginsberg JP, Kazak AE: **Prospectively examining physical activity in young adult survivors of childhood cancer and healthy controls**. *Pediatr Blood Cancer* 2013, **60**:309-315.

92. Hornsby WE, Douglas PS, West MJ, Kenjale AA, Lane AR, Schwitzer ER, Ray KA, Herndon JE, Coan A, Gutierrez A *et al*: **Safety and efficacy of aerobic training in operable breast cancer patients receiving neoadjuvant chemotherapy: A phase II randomized trial**. *Acta Oncol* 2014, **53**:65-74.

93. Humpel N, Iverson DC: **Depression and quality of life in cancer survivors: is there a relationship with physical activity?** *Int J Behav Nutr Phys Act* 2007, **4**.

94. Humpel N, Iverson DC: **Sleep quality, fatigue and physical activity following a cancer diagnosis**. *Eur J Cancer Care* 2010, **19**:761-768.

95. Hunt-Shanks TT, Blanchard CM, Baker F, Hann D, Roberts CS, McDonald J, Livingston M, Witt C, Ruiterman J, Ampela R, Kaw OCK: **Exercise use as complementary therapy among breast and prostate cancer survivors receiving active treatment: Examination of exercise intention**. *Integr Cancer Ther* 2006, **5**:109-116.

96. Jacobsen PB, Phillips KM, Jim HS, Small BJ, Faul LA, Meade CD, Thompson L, Williams CC, Jr., Loftus LS, Fishman M, Wilson RW: **Effects of self-directed stress management training and home-based exercise on quality of life in cancer patients receiving chemotherapy: a randomized controlled trial**. *Psycho-Oncology* 2013, **22**:1229-1235.

97. Jaremka LM, Andridge RR, Fagundes CP, Alfano CM, Povoski SP, Lipari AM, Agnese DM, Arnold MW, Farrar WB, Yee LD, Carson IIIWE, Bekaii-Saab T, Martin, JrEW, Schmidt CR, Kiecolt-Glaser JK: **Pain, Depression, and Fatigue: Loneliness as a Longitudinal Risk Factor**. *Health Psychol* 2014, **33**:948-957.

98. Jeffreys M, McKenzie F, Firestone R, Gray M, Cheng S, Moala A, Pearce N, Ellison-Loschmann L: **A multi-ethnic breast cancer case-control study in New Zealand: evidence of differential risk patterns**. *Cancer Causes Control* 2013, **24**:135-152.

99. Jones LW, Cohen RR, Mabe SK, West MJ, Desjardins A, Vredenburgh JJ, Friedman AH, Reardon DA, Waner E, Friedman HS: **Assessment of physical functioning in recurrent glioma: Preliminary comparison of performance status to functional capacity testing**. *J Neurooncol* 2009, **94**:79-85.

100. Jones LW, Courneya KS: **Exercise counseling and programming preferences of cancer survivors**. *Cancer Pract* 2002, **10**:208-215.

101. Jones LW, Courneya KS: **Exercise discussions during cancer treatment consultations**. *Cancer Pract* 2002, **10**:66-74.

102. Jones LW, Courneya KS, Fairey AS, Mackey JR: **Effects of an oncologist's recommendation to exercise on self-reported exercise behavior in newly diagnosed breast cancer survivors: A single-blind, randomized controlled trial**. *Ann Behav Med* 2004, **28**:105-113.

103. Jones LW, Courneya KS, Fairey AS, Mackey JR: **Does the theory of planned behavior mediate the effects of an oncologist's recommendation to exercise in newly diagnosed breast cancer survivors? Results from a randomized controlled trial**. *Health Psychol* 2005, **24**:189-197.

104. Jones LW, Courneya KS, Mackey JR, Muss HB, Pituskin EN, Scott JM, Hornsby WE, Coan AD, Herndon JE, II, Douglas PS, Haykowsky M: **Cardiopulmonary function and age-related decline across the breast cancer survivorship continuum**. *J Clin Oncol* 2012, **30**:2530-2537.

105. Jones LW, Courneya KS, Vallance JKH, Ladha AB, Mant MJ, Belch AR, Reiman T: **Understanding the determinants of exercise intentions in multiple myeloma cancer survivors: An application of the theory of planned behavior**. *Cancer Nurs* 2006, **29**:167-175.

106. Jones LW, Courneya KS, Vallance JKH, Ladha AB, Mant MJ, Belch AR, Stewart DA, Reiman T: **Association between exercise and quality of life in multiple myeloma cancer survivors**. *Support Care Cancer* 2004, **12**:780-788.

107. Jones LW, Eves ND, Mackey JR, Peddle CJ, Haykowsky M, Joy AA, Courneya KS, Tankel K, Spratlin J, Reiman T: **Safety and feasibility of cardiopulmonary exercise testing in patients with advanced cancer**. *Lung Cancer* 2007, **55**:225-232.

108. Jones LW, Friedman AH, West MJ, Mabe SK, Fraser J, Kraus WE, Friedman HS, Tresch MI, Major N, Reardon DA: **Quantitative assessment of cardiorespiratory fitness, skeletal muscle function, and body composition in adults with primary malignant glioma**. *Cancer* 2010, **116**:695-704.

109. Jones LW, Guill B, Keir ST, Carter K, Friedman HS, Bigner DD, Reardon DA: **Patterns of exercise across the cancer trajectory in brain tumor patients**. *Cancer* 2006, **106**:2224-2232.

110. Jones LW, Guill B, Keir ST, Carter K, Friedman HS, Bigner DD, Reardon DA: **Exercise interest and preferences among patients diagnosed with primary brain cancer**. *Support Care Cancer* 2007, **15**:47-55.

111. Jones LW, Guill B, Keir ST, Carter K, Friedman HS, Bigner DD, Reardon DA: **Using the theory of planned behavior to understand the determinants of exercise intention in patients diagnosed with primary brain cancer**. *Psycho-Oncology* 2007, **16**:232-240.

112. Jones LW, Hornsby WE, Goetzinger A, Forbes LM, Sherrard EL, Quist M, Lane AT, West M, Eves ND, Gradison M, Coan A, Herndon JE, Abernethy AP: **Prognostic significance of functional capacity and exercise behavior in patients with metastatic non-small cell lung cancer**. *Lung Cancer* 2012, **76**:248-252.

113. Jones LW, Mourtzakis M, Peters KB, Friedman AH, West MJ, Mabe SK, Kraus WE, Friedman HS, Reardon DA: **Changes in functional performance measures in adults undergoing chemoradiation for primary malignant glioma: a feasibility study**. *Oncologist* 2010, **15**:636-647.

114. Jones LW, Peddle CJ, Eves ND, Haykowsky MJ, Courneya KS, Mackey JR, Joy AA, Kumar V, Winton TW, Reiman T: **Effects of presurgical exercise training on cardiorespiratory fitness among patients undergoing thoracic surgery for malignant lung lesions**. *Cancer* 2007, **110**:590-598.

115. Karvinen KH, Courneya KS, Campbell KL, Pearcey RG, Dundas G, Capstick V, Tonkin KS: **Exercise preferences of endometrial cancer survivors: A population-based study**. *Cancer Nurs* 2006, **29**:259-265.

116. Karvinen KH, Courneya KS, Campbell KL, Pearcey RG, Dundas G, Capstick V, Tonkin KS: **Correlates of exercise motivation and behavior in a population-based sample of endometrial cancer survivors: an application of the Theory of Planned Behavior**. *Int J Behav Nutr Phys Act* 2007, **4**:21.

117. Karvinen KH, Courneya KS, North S, Venner P: **Associations between exercise and quality of life in bladder cancer survivors: A population-based study**. *Cancer Epidemiol Biomarkers Prev* 2007, **16**:984-990.

118. Karvinen KH, Courneya KS, Plotnikoff RC, Spence JC, Venner PM, North S: **A prospective study of the determinants of exercise in bladder cancer survivors using the Theory of Planned Behavior**. *Support Care Cancer* 2009, **17**:171-179.

119. Karvinen KH, Courneya KS, Venner P, North S: **Exercise programming and counseling preferences in bladder cancer survivors: a population-based study**. *J Cancer Surviv* 2007, **1**:27-34.

120. Karvinen KH, Raedeke TD, Arastu H, Allison RR: **Exercise programming and counseling preferences of breast cancer survivors during or after radiation therapy**. *Oncol Nurs Forum* 2011, **38**:E326-E334.

121. Karvinen KH, Esposito D, Raedeke TD, Vick J, Walker PR: **Effect of an exercise training intervention with resistance bands on blood cell counts during chemotherapy for lung cancer: A pilot randomized controlled trial**. *SpringerPlus* 2014, **3**.

122. Keats MR, Courneya KS, Danielsen S, Whitsett SF: **Leisure-time physical activity and psychosocial well-being in adolescents after cancer diagnosis**. *J Pediatr Oncol Nurs* 1999, **16**:180-188.

123. Keats MR, Culos-Reed N: **A theory-driven approach to encourage physical activity in pediatric cancer survivors: A pilot study**. *J Sport Exerc Psychol* 2009, **31**:267-283.

124. Keats MR, Culos-Reed SN: **A community-based physical activity program for adolescents with cancer (Project TREK) - Program feasibility and preliminary findings**. *J Pediatr Hematol Oncol* 2008, **30**:272-280.

125. Keats MR, Culos-Reed SN, Courneya KS, McBride M: **An examination of physical activity behaviors in a sample of adolescent cancer survivors**. *J Pediatr Oncol Nurs* 2006, **23**:135-142.

126. Keats MR, Culos-Reed SN, Courneya KS, McBride M: **Understanding physical activity in adolescent cancer survivors: An application of the theory of planned behavior**. *Psycho-Oncology* 2007, **16**:448-457.

127. Kenjale AA, Hornsby WE, Crowgey T, Thomas S, Herndon Ii JE, Khouri MG, Lane AR, Bishop CE, Eves ND, Peppercorn J *et al*: **Pre-Exercise Participation Cardiovascular Screening in a Heterogeneous Cohort of Adult Cancer Patients**. *Oncologist* 2014, **19**:999-1005.

128. Krebs P, Coups EJ, Feinstein MB, Burkhalter JE, Steingart RM, Logue A, Park BJ, Ostroff JS: **Health behaviors of early-stage non-small cell lung cancer survivors**. *J Cancer Surviv* 2012, **6**:37-44.

129. Ladha AB, Courneya KS, Bell GJ, Field CJ, Grundy P: **Effects of acute exercise on neutrophils in pediatric acute lymphoblastic leukemia survivors: a pilot study**. *J Pediatr Hematol Oncol* 2006, **28**:671-677.

130. Lin YY, Wu YC, Rau KM, Lin CC: **Effects of physical activity on the quality of life in taiwanese lung cancer patients receiving active treatment or off treatment**. *Cancer Nurs* 2013, **36**:E35-E41.

131. Lin YY, Liu MF, Tzeng JI, Lin CC: **Effects of Walking on Quality of Life Among Lung Cancer Patients: A Longitudinal Study**. *Cancer Nurs,* in press.

132. Love C, Sabiston CM: **Exploring the links between physical activity and posttraumatic growth in young adult cancer survivors**. *Psycho-Oncology* 2011, **20**:278-286.

133. Mack DE, Meldrum LS, Wilson PM, Sabiston CM: **Physical activity and psychological health in breast cancer survivors: an application of basic psychological needs theory**. *Appl Psychol Health Well Being* 2013, **5**:369-388.

134. Mackenzie MJ, Carlson LE, Ekkekakis P, Paskevich DM, Culos-Reed SN: **Affect and mindfulness as predictors of change in mood disturbance, stress symptoms, and quality of life in a community-based yoga program for cancer survivors**. *Evidence-based Complementary and Alternative Medicine* 2013, in press.

135. Mackenzie MJ, Carlson LE, Paskevich DM, Ekkekakis P, Wurz AJ, Wytsma K, Krenz KA, McAuley E, Culos-Reed SN: **Associations between attention, affect and cardiac activity in a single yoga session for female cancer survivors: An enactive neurophenomenology-based approach**. *Conscious Cogn* 2014, **27**:129-146.

136. Maddocks M, Armstrong S, Wilcock A: **Exercise as a supportive therapy in incurable cancer: Exploring patient preferences**. *Psycho-Oncology* 2011, **20**:173-178.

137. McAuley E, White SM, Rogers LQ, Motl RW, Courneya KS: **Physical activity and fatigue in breast cancer and multiple sclerosis: Psychosocial mechanisms**. *Psychosom Med* 2010, **72**:88-96.

138. McGowan EL, North S, Courneya KS: **Randomized controlled trial of a behavior change intervention to increase physical activity and quality of life in prostate cancer survivors**. *Ann Behav Med* 2013, **46**:382-393.

139. McGowan EL, Speed-Andrews AE, Blanchard CM, Rhodes RE, Friedenreich CM, Cubs-Reed SN, Courneya KS: **Physical activity preferences among a population-based sample of colorectal cancer survivors**. *Oncol Nurs Forum* 2013, **40**:44-52.

140. McKenzie F, Ellison-Loschmann L, Jeffreys M, Firestone R, Pearce N, Romieu I: **Cigarette smoking and risk of breast cancer in a New Zealand multi-ethnic case-control study**. *PloS One* 2013, **8**:e63132.

141. McKenzie F, Ellison-Loschmann L, Jeffreys M, Firestone R, Pearce N, Romieu I: **Healthy lifestyle and risk of breast cancer for indigenous and non-indigenous women in New Zealand: A case control study**. *BMC Cancer* 2014, **14**.

142. McNeely ML, Parliament M, Courneya KS, Seikaly H, Jha N, Scrimger R, Hanson J: **A pilot study of a randomized controlled trial to evaluate the effects of progressive resistance exercise training on shoulder dysfunction caused by spinal accessory neurapraxia/neurectomy in head and neck cancer survivors**. *Head Neck* 2004, **26**:518-530.

143. Mesa RA, Niblack J, Wadleigh M, Verstovsek S, Camoriano J, Barnes S, Tan AD, Atherton PJ, Sloan JA, Tefferi A: **The burden of fatigue and quality of life in myeloproliferative disorders (MPDs): An international Internet-based survey of 1179 MPD patients**. *Cancer* 2007, **109**:68-76.

144. Milne HM, Gordon S, Guilfoyle A, Wallman KE, Courneya KS: **Association between physical activity and quality of life among Western Australian breast cancer survivors**. *Psycho-Oncology* 2007, **16**:1059-1068.

145. Milne HM, Wallman KE, Guilfoyle A, Gordon S, Courneya KS: **Self-determination theory and physical activity among breast cancer survivors**. *J Sport Exerc Psychol* 2008, **30**:23-38.

146. Mina DS, Connor MK, Alibhai SM, Toren P, Guglietti C, Matthew AG, Trachtenberg J, Ritvo P: **Exercise effects on adipokines and the IGF axis in men with prostate cancer treated with androgen deprivation: A randomized study**. *Can Urol Assoc J* 2013, **7**:E692-E698.

147. Musanti R: **A study of exercise modality and physical self-esteem in breast cancer survivors**. *Med Sci Sports Exerc* 2012, **44**:352-361.

148. Norris JM, Moules NJ, Pelletier G, Culos-Reed SN: **Families of young pediatric cancer survivors: A cross-sectional survey examining physical activity behavior and health-related quality of life**. *J Pediatr Oncol Nurs* 2010, **27**:196-208.

149. O'Carroll Bantum E, Albright CL, White KK, Berenberg JL, Layi G, Ritter PL, Laurent D, Plant K, Lorig K: **Surviving and thriving with cancer using a web-based health behavior change intervention: Randomized controlled trial**. *J Med Internet Res* 2014, **16**.

150. Olson EA, Mullen SP, Rogers LQ, Courneya KS, Verhulst S, McAuley E: **Meeting physical activity guidelines in rural breast cancer survivors**. *Am J Health Behav* 2014, **38**:890-899.

151. Packel LB, Prehn AW, Anderson CL, Fisher PL: **Factors Influencing Physical Activity Behaviors in Colorectal Cancer Survivors**. *Am J Health Promot* 2014, in press.

152. Paxton RJ, Jones LW, Rosoff PM, Bonner M, Ater JL, Demark-Wahnefried W: **Associations between leisure-time physical activity and health-related quality of life among adolescent and adult survivors of childhood cancers**. *Psycho-oncology* 2010, **19**:997-1003.

153. Peddle CJ, Au HJ, Courneya KS: **Associations between exercise, quality of life, and fatigue in colorectal cancer survivors**. *Dis Colon Rectum* 2008, **51**:1242-1248.

154. Peddle CJ, Plotnikoff RC, Wild TC, Au H-J, Courneya KS: **Medical, demographic, and psychosocial correlates of exercise in colorectal cancer survivors: an application of self-determination theory**. *Support Care Cancer* 2008, **16**:9-17.

155. Peeters C, Stewart A, Segal R, Wouterloot E, Scott CG, Aubry T: **Evaluation of a cancer exercise program: patient and physician beliefs**. *Psycho-Oncology* 2009, **18**:898-902.

156. Perkins HY, Waters AJ, Baum GP, Basen-Engquist KM: **Outcome expectations, expectancy accessibility, and exercise in endometrial cancer survivors**. *J Sport Exerc Psychol* 2009, **31**:776-785.

157. Perna FM, Craft L, Freund KM, Skrinar G, Stone M, Kachnic L, Youren C, Battaglia TA: **The effect of a cognitive behavioral exercise intervention on clinical depression in a multiethnic sample of women with breast cancer: A randomized controlled trial**. *Int J Sport Exerc Psychol* 2010, **8**:36-47.

158. Philip EJ, Coups EJ, Feinstein MB, Park BJ, Wilson DJ, Ostroff JS: **Physical activity preferences of early-stage lung cancer survivors**. *Support Care Cancer* 2014, **22**:495-502.

159. Phillips KM, Jim HS, Donovan KA, Pinder-Schenck MC, Jacobsen PB: **Characteristics and correlates of sleep disturbances in cancer patients**. *Supportive Care Cancer* 2012, **20**:357-365.

160. Phillips SM, McAuley E: **Physical activity and fatigue in breast cancer survivors: A panel model examining the role of self-efficacy and depression**. *Cancer Epidemiol Biomarkers Prev* 2013, **22**:773-781.

161. Phillips SM, McAuley E: **Social cognitive influences on physical activity participation in long-term breast cancer survivors**. *Psycho-Oncology* 2013, **22**:783-791.

162. Phillips SM, McAuley E: **Physical activity and quality of life in breast cancer survivors: The role of self-efficacy and health status**. *Psycho-Oncology* 2014, **23**:27-34.

163. Rogers LQ, Courneya KS, Robbins KT, Malone J, Seiz A, Koch L, Rao K: **Physical activity correlates and barriers in head and neck cancer patients**. *Support Care Cancer* 2008, **16**:19-27.

164. Rogers LQ, Courneya KS, Robbins KT, Malone J, Seiz A, Koch L, Rao K, Nagarkar M: **Physical activity and quality of life in head and neck cancer survivors**. *Support Care Cancer* 2006, **14**:1012-1019.

165. Rogers LQ, Courneya KS, Robbins KT, Rao K, Malone J, Seiz A, Reminger S, Markwell SJ, Burra V: **Factors associated with fatigue, sleep, and cognitive function among patients with head and neck cancer**. *Head Neck* 2008, **30**:1310-1317.

166. Rogers LQ, Courneya KS, Verhulst S, Markwell SJ, McAuley E: **Factors associated with exercise counseling and program preferences among breast cancer survivors**. *J Phys Act Health* 2008, **5**:688-705.

167. Rogers LQ, Hopkins-Price P, Vicari S, Pamenter R, Courneya KS, Markwell S, Verhulst S, Hoelzer K, Naritoku C, Jones L, Dunnington G, Lanzotti V, Wynstra J, Shah L, Edson B, Graff A, Lowy M: **A randomized trial to increase physical activity in breast cancer survivors**. *Med Sci Sports Exerc* 2009, **41**:935-946.

168. Rogers LQ, Malone J, Rao K, Courneya KS, Fogleman A, Tippey A, Markwell SJ, Robbins KT: **Exercise preferences among patients with head and neck cancer: Prevalence and associations with quality of life, symptom severity, depression, and rural residence**. *Head Neck* 2009, **31**:994-1005.

*169. Rogers LQ, Markwell SJ, Courneya KS, McAuley E, Verhulst S: **Exercise preference patterns, resources, and environment among rural breast cancer survivors**. *J Rural Health* 2009, **25**(4):388-391.

170. Rogers LQ, Markwell SJ, Verhulst S, McAuley E, Courneya KS: **Rural breast cancer survivors: exercise preferences and their determinants**. *Psycho-Oncology* 2009, **18**:412-421.

171. Rogers LQ, McAuley E, Courneya KS, Verhulst SJ: **Correlates of physical activity self-efficacy among breast cancer survivors**. *Am J Health Behav* 2008, **32**:594-603.

172. Ruden E, Reardon DA, Coan AD, Herndon Ii JE, Hornsby WE, West M, Fels DR, Desjardins A, Vredenburgh JJ, Waner E, Friedman AH, Friedman HS, Peters KB, Jones LW: **Exercise behavior, functional capacity, and survival in adults with malignant recurrent glioma**. *J Clin Oncol* 2011, **29**:2918-2923.

173. Santa Mina D, Alibhai SM, Matthew AG, Guglietti CL, Pirbaglou M, Trachtenberg J, Ritvo P: **A randomized trial of aerobic versus resistance exercise in prostate cancer survivors**. *J Aging Phys Act* 2013, **21**:455-478.

174. Santa Mina D, Guglietti CL, Alibhai SMH, Matthew AG, Kalnin R, Ahmad N, Lindner U, Trachtenberg J: **The effect of meeting physical activity guidelines for cancer survivors on quality of life following radical prostatectomy for prostate cancer**. *J Cancer Surviv* 2014, **8**:190-198.

175. Santa Mina D, Guglietti CL, de Jesus DR, Azargive S, Matthew AG, Alibhai SMH, Trachtenberg J, Daskalakis JZ, Ritvo P: **The acute effects of exercise on cortical excitation and psychosocial outcomes in men treated for prostate cancer: A randomized controlled trial**. *Front Aging Neurosci* 2014, in press.

176. Serdà BC, Monreal P, Del Valle A: **Physical exercise as complementary treatment in prostate cancer**. *Apunts Med Esport* 2010, **45**:81-93.

177. Short CE, James EL, Vandelanotte C, Courneya KS, Duncan MJ, Rebar A, Plotnikoff RC: **Correlates of resistance training in post-treatment breast cancer survivors**. *Support Care Cancer* 2014, **22**:2757-2766.

178. Speed-Andrews AE, Rhodes RE, Blanchard CM, Culos-Reed SN, Friedenreich CM, Belanger LJ, Courneya KS: **Medical, demographic and social cognitive correlates of physical activity in a population‐based sample of colorectal cancer survivors**. *Eur J Cancer Care* 2012, **21**:187-196.

179. Speed-Andrews AE, Stevinson C, Belanger LJ, Mirus JJ, Courneya KS: **Pilot evaluation of an Iyengar yoga program for breast cancer survivors**. *Cancer Nurs* 2010, **33**:369-381.

180. Steensma DP, Heptinstall KV, Johnson VM, Novotny PJ, Sloan JA, Camoriano JK, Niblack J, Bennett JM, Mesa RA: **Common troublesome symptoms and their impact on quality of life in patients with myelodysplastic syndromes (MDS): Results of a large internet-based survey**. *Leuk Res* 2008, **32**:691-698.

181. Stephenson LE, Bebb DG, Reimer RA, Culos-Reed SN: **Physical activity and diet behaviour in colorectal cancer patients receiving chemotherapy: associations with quality of life**. *BMC Gastroenterology* 2009, **9**.

182. Stevinson C, Capstick V, Schepansky A, Tonkin K, Vallance JK, Ladha AB, Steed H, Faught W, Courneya KS: **Physical activity preferences of ovarian cancer survivors**. *Psycho-Oncology* 2009, **18**:422-428.

183. Stevinson C, Faught W, Steed H, Tonkin K, Ladha AB, Vallance JK, Capstick V, Schepansky A, Courneya KS: **Associations between physical activity and quality of life in ovarian cancer survivors**. *Gynecol Oncol* 2007, **106**:244-250.

184. Stevinson C, Steed H, Faught W, Tonkin K, Vallance JK, Ladha AB, Schepansky A, Capstick V, Courneya KS: **Physical activity in ovarian cancer survivors: associations with fatigue, sleep, and psychosocial functioning**. *Int J Gynecol Cancer* 2009, **19**:73-78.

185. Stevinson C, Tonkin K, Capstick V, Schepansky A, Ladha AB, Vallance JK, Faught W, Steed H, Courneya KS: **A Population-based study of the determinants of physical activity in ovarian cancer survivors**. *J Phys Act Health* 2009, **6**:339-346.

186. Stevinson C, Lydon A, Amir Z: **Adherence to physical activity guidelines among cancer support group participants**. *Eur J Cancer Care* 2014, **23**:199-205.

187. Tillmann V, Darlington AS, Eiser C, Bishop NJ, Davies HA: **Male sex and low physical activity are associated with reduced spine bone mineral density in survivors of childhood acute lymphoblastic leukemia**. *J Bone Miner Res* 2002, **17**:1073-1080.

188. Trinh L, Plotnikoff RC, Rhodes RE, North S, Courneya KS: **Associations between physical activity and quality of life in a population-based sample of kidney cancer survivors**. *Cancer Epidemiol Biomarkers Prev* 2011, **20**:859-868.

189. Trinh L, Plotnikoff RC, Rhodes RE, North S, Courneya KS: **Correlates of physical activity in a population-based sample of kidney cancer survivors: an application of the theory of planned behavior**. *Int J Behav Nutr Phys Act* 2012, **9**.

190. Trinh L, Plotnikoff RC, Rhodes RE, North S, Courneya KS: **Physical activity preferences in a population-based sample of kidney cancer survivors**. *Support Care Cancer* 2012, **20**:1709-1717.

191. Trinh L, Plotnikoff RC, Rhodes RE, North S, Courneya KS: **Associations between sitting time and quality of life in a population-based sample of kidney cancer survivors**. *Ment Health Phy Act* 2013, **6**:16-23.

192. Trinh L, Plotnikoff RC, Rhodes RE, North S, Courneya KS: **Feasibility and preliminary efficacy of adding behavioral counseling to supervised physical activity in kidney cancer survivors: A randomized controlled trial**. *Cancer Nurs* 2014, **37**:E8-E22.

193. Tyrrell A, Keats M, Blanchard CM: **The Physical Activity Preferences of Gynecologic Cancer Survivors**. *Oncology Nurs Forum* 2014, **41**:461-469.

194. Valenti M, Porzio G, Aielli F, Verna L, Cannita K, Manno R, Masedu F, Marchetti P, Ficorella C: **Physical exercise and quality of life in breast cancer survivors**. *Int J Med Sci* 2008, **5**:24-28.

195. Vallance J, Lavallee C, Culos-Reed N, Trudeau M: **Rural and small town breast cancer survivors' preferences for physical activity**. *Int J Behav Med* 2013, **20**:522-528.

196. Vallance J, Plotnikoff RC, Karvinen KH, Mackey JR, Courneya KS: **Understanding physical activity maintenance in breast cancer survivors**. *Am J Health Behav* 2010, **34**:225-236.

197. Vallance JK, Courneya KS, Plotnikoff RC, Dinu I, Mackey JR: **Maintenance of physical activity in breast cancer survivors after a randomized trial**. *Med Sci Sports Exerc* 2008, **40**:173-180.

198. Vallance JK, Lavallee C, Culos-Reed NS, Trudeau MG: **Predictors of physical activity among rural and small town breast cancer survivors: An application of the theory of planned behaviour**. *Psychol Health Med* 2012, **17**:685-697.

199. Vallance JK, Lavallee CM, Culos-Reed NS, Trudeau MG: **Physical activity is associated with clinically important differences in health-related quality of life among rural and small-town breast cancer survivors**. *Support Care Cancer* 2012, **20**:1079-1087.

200. Vallance JKH, Courneya KS, Jones LW, Reiman T: **Differences in quality of life between non-Hodgkin's lymphoma survivors meeting and not meeting public health exercise guidelines**. *Psycho-Oncology* 2005, **14**:979-991.

201. Vallance JKH, Courneya KS, Jones LW, Reiman T: **Exercise preferences among a population-based sample of non-Hodgkin's lymphoma survivors**. *Eur J Cancer Care* 2006, **15**:34-43.

202. Vallance JKH, Courneya KS, Plotnikoff RC, Mackey JR: **Analyzing theoretical mechanisms of physical activity behavior change in breast cancer survivors: Results from the Activity Promotion (ACTION) trial**. *Ann Behav Med* 2008, **35**:150-158.

203. Vallance JKH, Courneya KS, Plotnikoff RC, Yasui Y, Mackey JR: **Randomized controlled trial of the effects of print materials and step pedometers on physical activity and quality of life in breast cancer survivors**. *J Clinical Oncol* 2007, **25**:2352-2359.

204. Valle CG, Tate DF, Mayer DK, Allicock M, Cai J: **A randomized trial of a Facebook-based physical activity intervention for young adult cancer survivors**. *J Cancer Surviv* 2013, **7**:355-368.

205. von Gruenigen V, Frasure H, Kavanagh MB, Janata J, Waggoner S, Rose P, Lerner E, Courneya KS: **Survivors of uterine cancer empowered by exercise and healthy diet (SUCCEED): a randomized controlled trial**. *Gynecol Oncol* 2012, **125**:699-704.

206. von Gruenigen VE, Courneya KS, Gibbons HE, Kavanagh MB, Waggoner SE, Lerner E: **Feasibility and effectiveness of a lifestyle intervention program in obese endometrial cancer patients: a randomized trial**. *Gynecol Oncol* 2008, **109**:19-26.

207. von Gruenigen VE, Frasure HE, Kavanagh MB, Lerner E, Waggoner SE, Courneya KS: **Feasibility of a lifestyle intervention for ovarian cancer patients receiving adjuvant chemotherapy**. *Gynecol Oncol* 2011, **122**:328-333.

208. von Gruenigen VE, Waggoner SE, Frasure HE, Kavanagh MB, Janata JW, Rose PG, Courneya KS, Lerner E: **Lifestyle challenges in endometrial cancer survivorship**. *Obstet Gynecol* 2011, **117**:93-100.

209. Wang Y-J, Boehmke M, Wu Y-WB, Dickerson SS, Fisher N: **Effects of a 6-week walking program on taiwanese women newly diagnosed with early-stage breast cancer**. *Cancer Nurs* 2011, **34**:E1-E13.

210. Wright M, Bryans A, Gray K, Skinner L, Verhoeve A: **Physical Activity in Adolescents following Treatment for Cancer: Influencing Factors**. *Leuk Res Treatment* 2013, **2013**:7.

211. Wrosch C, Sabiston CM: **Goal adjustment, physical and sedentary activity, and well-being and health among breast cancer survivors**. *Psycho-Oncology* 2013, **22**:581-589.

212. Wurz A, Chamorro-Vina C, Guilcher GMT, Schulte F, Culos-Reed SN: **The feasibility and benefits of a 12-week yoga intervention for pediatric cancer out-patients**. *Pediatr Blood Cancer* 2014, **61**:1828-1834.

213. Zahavich ANR, Robinson JA, Paskevich D, Culos-Reed SN: **Examining a therapeutic yoga program for prostate cancer survivors**. *Integr Cancer Ther* 2013, **12**:113-125.
